# Self-Oscillatory Neuron-like Devices for Unconventional Computing Applications

**DOI:** 10.1021/acs.chemrev.5c00878

**Published:** 2026-05-13

**Authors:** Gonzalo Rivera-Sierra, Juan Bisquert, Roberto Fenollosa

**Affiliations:** † 83167 Instituto de Tecnología Química (ITQ), Consejo Superior de Investigaciones Científicas-Universitat Politècnica de València, 46022 València, Spain

## Abstract

Self-sustained oscillators are emerging as key physical elements for neuromorphic electronics, providing a hardware route to emulate the spiking dynamics of biological neurons. As conventional computing architectures struggle with power dissipation and parallel processing limitations, oscillatory devices offer a means to reproduce the brain’s remarkable efficiency–performing adaptive and nonlinear tasks with minimal energy consumption. This review provides a unified synthesis of the diverse families of self-oscillating systems developed across physics, chemistry, and electronic engineering. We classify oscillators according to their operational mechanisms, distinguishing those driven by negative differential resistance (NDR) instabilities from those sustained by active-feedback amplifiers. Their common behavior is described within a nonlinear dynamical framework that links materials, electronic response, and the emergence of limit cycles in phase space. We discuss how these devices–ranging from electrochemical and memristive oscillators to transistor-based and hybrid architectures–can be modeled, measured, and coupled to form complex networks. Particular attention is given to the experimental identification of active elements and impedance signatures that reveal self-oscillation. By bridging device physics, nonlinear dynamics, and neuromorphic computing, this review outlines a coherent foundation for designing scalable, energy-efficient oscillatory systems that connect the physical principles of chemical and electronic oscillators with the computational logic of the brain.

## Graphical abstract

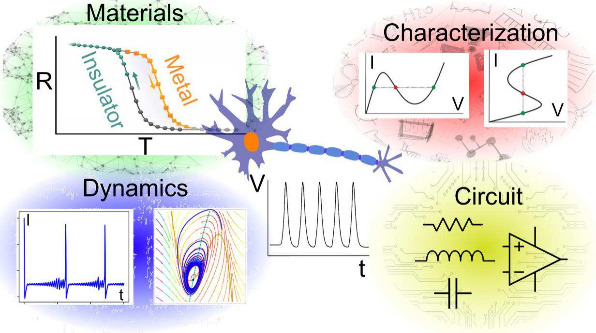

## 1 Introduction

The growing demand for energy-efficient and adaptive computing has spurred interest in neuromorphic systems, where hardware is inspired by the architecture and function of the human brain.^1–6^ While modern supercomputers offer immense processing power and storage capacity, they remain orders of magnitude less efficient than the brain, consuming vastly more energy and space to perform comparable tasks. As artificial intelligence (AI) applications continue to scale, conventional von Neumann architectures struggle to keep up with the energy, scalability, and latency demands of real-time data processing.

In contrast to conventional computing systems, the human brain operates with extraordinary efficiency, consuming only about 20 W of power while performing highly complex tasks such as perception, learning, pattern recognition, and decision-making. This exceptional balance of energy efficiency and computational power has inspired the exploration of alternative computing models that move beyond traditional architectures. Among these, neuromorphic computing has emerged as a particularly promising paradigm. By emulating the brain’s structure and dynamics, neuromorphic systems aim to replicate key cognitive functions such as learning from experience, adapting to new information, and processing data in real time–all while maintaining ultralow power consumption.

Unlike conventional processors, which separate memory and computation, neuromorphic architectures integrate these functions in a distributed and parallel fashion, closely resembling how neurons and synapses operate in the brain. This design enables fast, event-driven responses and significant reductions in energy usage, making neuromorphic systems highly suitable for edge computing applications. When combined with sensors, these systems can process data locally–without the need to communicate constantly with centralized cloud servers–resulting in low-latency, autonomous decision-making capabilities. Such characteristics make neuromorphic technology an attractive solution for a wide range of applications, including robotics, wearable health monitors, environmental sensing, and other scenarios where compact form factors and energy efficiency are critical.^7–9^

We can gain valuable insights for neuromorphic computing by studying and embracing the unique neurobiological structures and computational patterns found in animals with exceptional sensory abilities, as the example shown in Figure 1. The barn owl’s brainstem, particularly notable for its specialized auditory processing capabilities, support the owl’s exceptional ability to localize sounds.^10^ Over millions of years, the neural organization and synaptic connections in these natural “super sensors” have evolved to process complex sensory information with remarkable precision and efficiency, even in harsh, resource-limited environments. These biological systems achieve powerful sensory performance while consuming very little energy. By mimicking these advanced neural architectures and the algorithms they support, we can explore a promising new path: developing energy-efficient neuromorphic computing systems using innovative devices and circuits inspired by nature.

**Figure 1:**
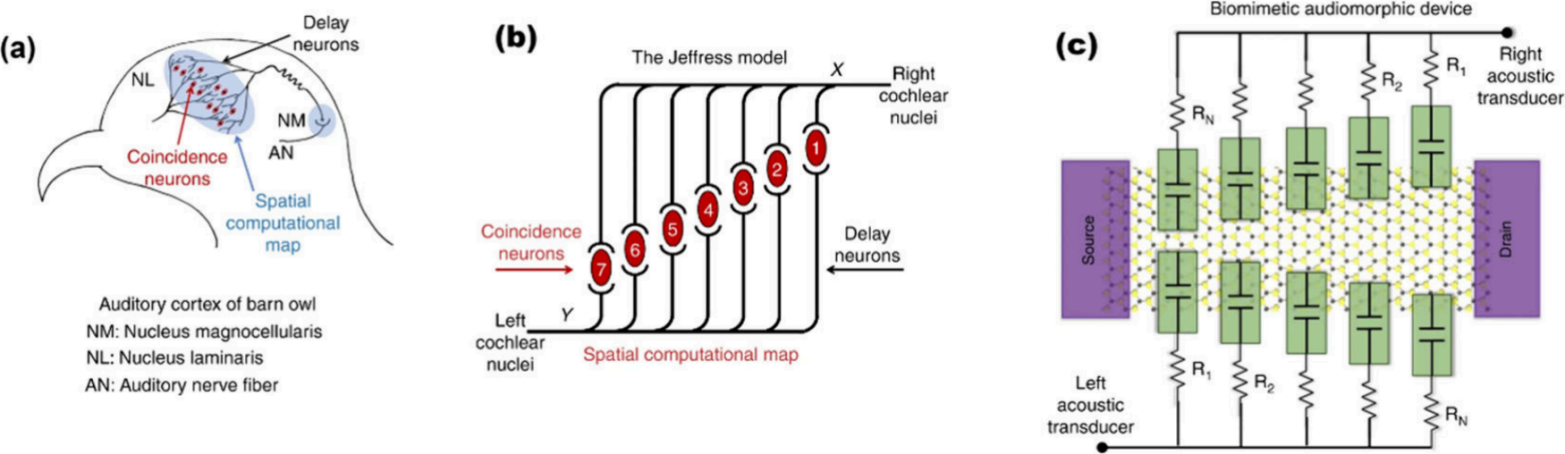
(a) An anatomical drawing of barn owl’s brainstem. Axons from the nucleus magnocellularis (NM) provide the delay lines, whereas, binaural tertiary nerve fibers serve as coincidence detectors in the nucleus laminaris (NL). (b) The Jeffress model showing the neural computational map in the auditory cortex for transforming temporal coding into spatial coding by deploying two key neural elements–the time delay neurons and the coincidence detector neurons. The coincidence neurons fire only when spikes arrive concurrently at the corresponding delay neurons. The model shows remarkable similarity with the owl’s brainstem. (c) A fully integrated biomimetic audiomorphic device emulating the neural computational map involving multiple split-gates with different widths of the ungated region on a single MoS_2_ channel. Each split-gate is connected to a delay line resistor with the resistance value designed in accordance with the spatial location of the corresponding split-gate. Reproduced from ref 10. Available under a CC-BY 4.0 license. Copyright 2019 The Author(s). Published by Springer Nature.

At the foundation of these advancements lies the neuron, the brain’s fundamental unit for processing and transmitting information.^11^ Neural control of behavior follows a three-step process: sensory neurons convert environmental stimuli into neural signals; these signals are processed in the brain or central nervous system; and, based on this processing, motor commands are generated to produce coordinated muscle activity, resulting in behavior.^12^ Biological neurons communicate through spiking signals, a process in which a periodic voltage signal forms a nonsinusoidal oscillation governed by voltage-gated ion channels within the neuronal membrane. The voltage difference across the membrane is maintained by concentration differences of Na^+^ and K^+^ ions, as shown in Figure 2. An incoming voltage perturbation process launches the opening and closing of the channels, enabling the corresponding ion fluxes, which cause the action potential shape shown in Figure 2b. A repetitive pattern caused by the constant perturbation is the spiking behavior shown in Figure 2c. This behavior is well described by the Hodgkin-Huxley (HH) model, which accurately captures the timing and waveform of action potentials observed in living neurons.^13–15^

**Figure 2:**
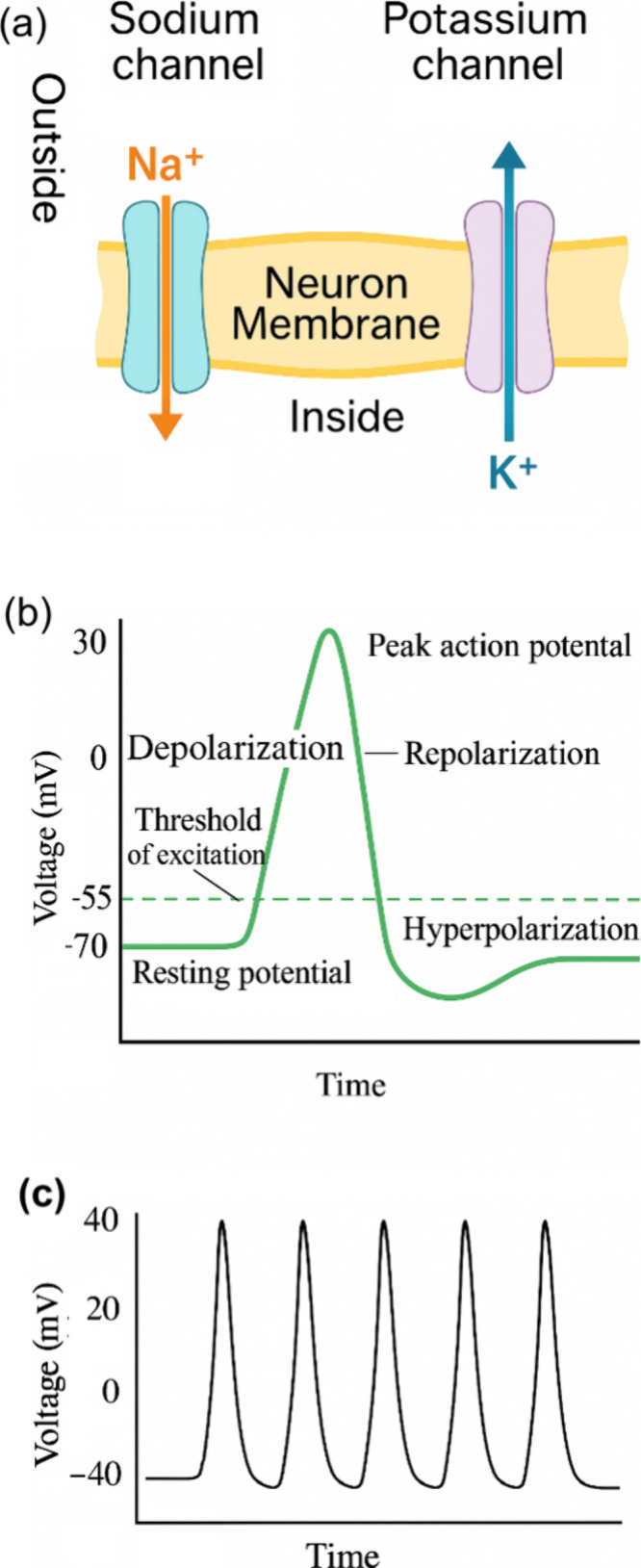
Basic mechanism of neuron spiking. (a) Ion fluxes across the ion channels in the neuron membrane. (b) The action potential is a rapid and transient change in the membrane potential of a neuron that allows it to transmit electrical signals along its axon. At the resting state (approximately 70 mV), the neuron maintains a stable negative internal environment. This is due to a higher concentration of potassium ions (K^+^) inside the cell and sodium ions (Na^+^) outside, maintained by the Na^+^/K^+^ pump and leaky K^+^ channels. When a sufficient stimulus depolarizes the membrane to the threshold of excitation (around 55 mV), voltage-gated Na^+^ channels open. During the depolarization phase, Na^+^ ions rapidly enter the cell, causing the membrane potential to rise steeply and reverse polarity, reaching a peak action potential of about +30 mV. At the peak, Na^+^ channels inactivate, and voltage-gated K^+^ channels open, initiating repolarization. K^+^ ions exit the neuron, driving the membrane potential back toward negative values. This outflow continues during the hyperpolarization phase, where the membrane potential becomes more negative than the resting potential (around 80 mV) due to the delayed closure of K^+^ channels. Finally, during the return to resting potential, ion channels reset and the Na^+^/K^+^ pump gradually restores the original ion distribution, bringing the membrane back to approximately 70 mV and readying the neuron for another action potential. (c) Repetitive action potentials form neuron spiking regime.

This review focuses on a particular kind of electrical device known as a self-oscillating system, that enables to emulate the spiking properties of natural neurons.^16,17^ In nature, there are many examples where oscillations (repeating back-and-forth motions) are sustained by a steady input from the outside.^18^ In this type of system, a periodic (repeating) signal is generated and maintained using a constant, nonperiodic source of energy, that is normally produced by a constant voltage or current source. In other words, even though the power source itself does not fluctuate or vary in a regular pattern, the device somehow organizes that steady input into a repeating output.

What makes this phenomenon particularly compelling is that the oscillation is not merely the result of an externally imposed rhythm. Rather, the system harnesses its own internal dynamics to regulate the interaction with its energy source. In this framework, the oscillation effectively governs its own timing, determining the phase and duration during which energy is absorbed or released. This self-organized control enables the system to synchronize energy intake with its functional needs, enhancing both efficiency and adaptability.

This concept reflects Arthur Winfree’s foundational work on biological oscillators,^19,20^ which revealed how systems like circadian rhythms and neural patterns can self-regulate and synchronize through nonlinear internal dynamics. Crucially, they respond to external cues without losing internal coherence. In neuromorphic and bioinspired systems, similarly, the oscillation phase serves as a control signal-enabling robust, context-sensitive regulation of energy flow. This phase-based control supports efficient, autonomous operation, making it ideal for low-power, adaptive devices in resource-constrained environments. Figure 3(a,b) shows an example in which two oscillators synchronize in or out-of-phase, depending on the strength of the interaction through a resistor.^21^ Unlike analog non-volatile memory elements that rely primarily on static nonlinear input-output characteristics, self-sustained oscillators enable a fundamentally dynamical mode of computation. In oscillator-based systems, information is encoded and processed through temporal variables such as oscillation phase, frequency, and synchronization, rather than through steady-state signal amplitudes. This time-domain computational paradigm provides a direct conceptual link between the oscillator circuits shown in Figure 3, the neuron-inspired dynamical model illustrated in Figure 2, and biological sensory systems such as the barn owl auditory pathway in Figure 1, where precise timing, coincidence detection, and collective oscillatory activity play a central role in information processing.

**Figure 3:**
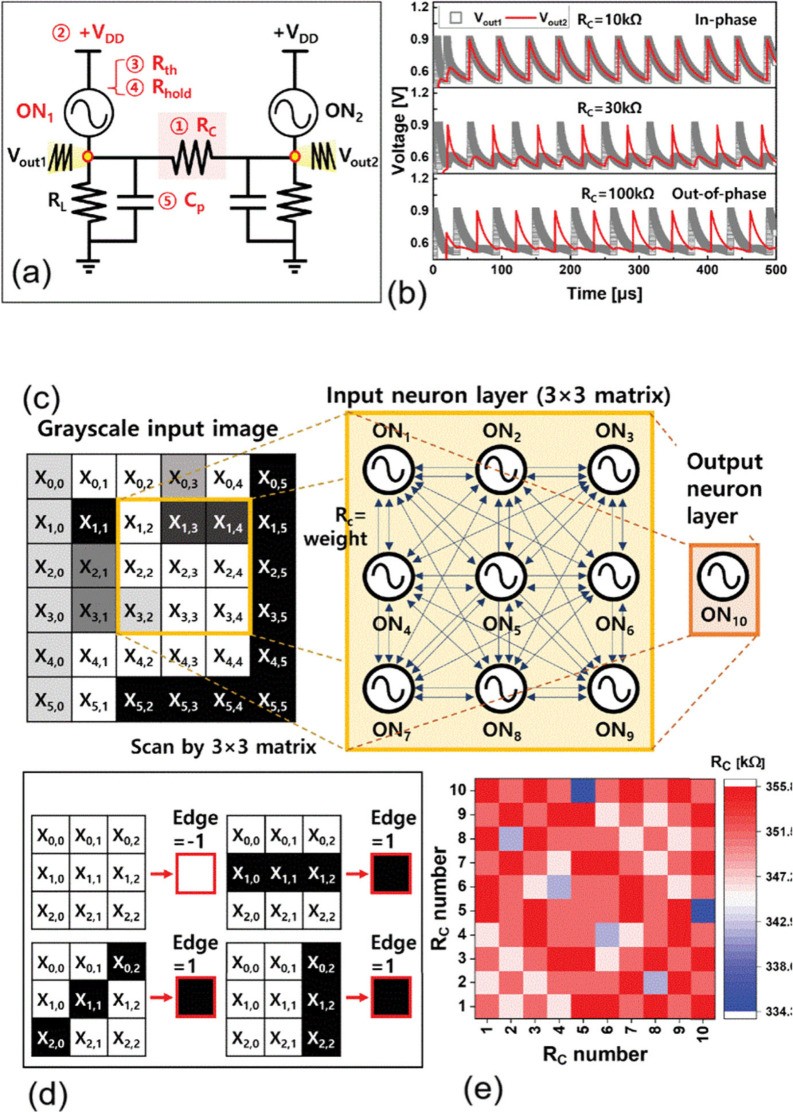
(a) Configuration of the coupled-oscillatory neuron system. Each neuron is an oscillator consisting on 20 nm-thick NbO_*x*_ layer formed via a reactive sputtering process. (b) In-phase synchronization became out-of-phase when R_c_ was increased to 100 kΩ in the HSPICE simulation. (c) Constructed ONN system comprising nine neurons and one neuron for input neuron and output neuron, respectively, to detect the edge of the given image. (d) Patterns were trained based on Hebbian learning rule. (e) Weight mapping in the form of Rc in the ONN systems to infer the patterns. Reproduced with permission from ref 21. Copyright 2023 IEEE.

The review aims at a survey of neuron-like self-sustained oscillators, examining their fundamental properties and categorizing the physical and material requirements necessary for compact oscillatory elements. We will emphasize the description of oscillators by a set of differential equations, aiming to understand the physicochemical basis for self-oscillators, and the practical application of this knowledge in real material oscillators of different kinds.^22–27^ Both in natural neurons and in artificial neuron oscillators, the repetitive potential spikes are obtained by a *bifurcation* that occurs when a parameter value causes a stable equilibrium point to become unstable, leading to the emergence of a stable periodic orbit–a limit cycle.^13–15,28–30^ For example, a compact self-sustained oscillator is obtained by binary oxide memristors as NbO_2_ ^31–35^ and VO_2_,^36,37^ as shown in Figure 3, widely employed in computation with coupled oscillators.^38–40^

This general class of oscillators is different from systems like resonant circuits, for example an LC (inductor-capacitor) circuit. In those systems, a periodic output can also be produced, but only if the input power already has a regular, periodic character. The LC circuit responds to and reflects the rhythm of the external signal; it does not create its own timing from a nonperiodic input.

In this context, it is useful to distinguish between passive and active circuit elements. Passive circuit elements do not supply energy; they only consume or store it. Examples are the resistor, capacitor and inductor, all of which have a linear property. On the other hand, active circuit elements can supply energy and often control current or voltage. These are nonlinear elements such as transistors, operational amplifiers (Op-Amps) and batteries (DC sources). They can amplify signals, perform switching, and are necessary for sustained oscillations or gain. While a forced resonator is a passive device, self-sustained oscillators contain a negative damping term that draws energy from its surroundings. Hence an active element is necessary to generate and maintain a regular periodicity without requiring a similar external periodicity to drive it.^18,41^ A negative differential resistance (NDR) element will be a protagonist in our review. It always acts as an active element because it supplies energy and requires external power or active circuitry to function.^42^

Self-sustained oscillators have been studied for two centuries.^18^ In 1830, G. B. Airy published a brief paper that is considered the first mathematical treatment of self-oscillation. In 1908, Henri Poincaré introduced the concept of a “limit cycle” and in 1919 Blondel introduced the term “self-sustained oscillations”. Building upon these foundations, Balthasar van der Pol introduced the concept of “relaxation oscillations” in 1926. Thereafter occurred the explosion of research in several areas: electronic oscillators,^41,43^ the mathematics of nonlinear dynamical systems and bifurcation,^44^ and developments in many branches of chemistry,^23^ physics,^18^ and electrochemistry.^27,45–47^ An important cornerstone is the biological neuron oscillator in neuroscience^48,49^ that provides the main inspiration for neuromorphic systems.^50^ The seminal work of Carver Mead^50^ laid the foundation for neuromorphic engineering by demonstrating how the principles of analog very large scale integration (VLSI) circuits could be used to mimic the behavior of biological neurons, enabling the development of hardware that processes information in a brain-like, energy-efficient manner. The work of Winfree^19,20^ and Kuramoto^51^ launched an understanding of the critical effects of synchronization in large networks of oscillators.

This review extends the analysis of neuron-like oscillators^30,42,52^ by adopting a broader perspective, integrating diverse yet often disconnected areas: classical electronic oscillators, including feedback-based oscillators, engineering control theory, bifurcation theory in nonlinear dynamical systems, and the physicochemical mechanisms underlying self-sustained oscillations in material device systems. We outline the operational conditions that define the parametric regimes in which oscillations occur, offering insight into the material-chemical characteristics of the devices and in the external RC components that enable both oscillatory behavior and coupling of the transmitted signal between oscillators in a network.

In addition, we analyze the amplification requirements of the active feedback stage, as found in conventional feedback oscillators without emergent device components, in order to generalize the criteria for self-sustained oscillation across both classes of systems and later mix them and be capable of describing all types of self-oscillators in a unified framework.

Out of the vast and multidisciplinary nature of the subject, here we reference only key sources that offer deeper technical detail. Since this paper is intended to be a useful tool for chemists and material scientists interested in new types of oscillators, we provide a presentation that illustrates very basic mechanisms of oscillations, and we merge the different approaches used in the broad variety of fields where oscillators have been developed. Our aim is not to provide an exhaustive treatment, but rather to present a cohesive overview of the key factors that define oscillator classes and their principles of operation.

One major objective of this study is to classify the primary types of emergent self-oscillatory devices and identify the key properties that determine their modes of operation, drawing on principles derived from natural neurons–exemplified by the HH model–and related tools from computational neuroscience. The description of an oscillator in terms of a set of first-order differential equations is emphasized through the review. Current knowledge enables us to formulate numerous properties in mathematical terms. Therefore, we explore nonlinear dynamical modeling as a particularly suitable and effective framework for simulating the functioning of various classes of oscillators. The methods for nonlinear dynamics relevant to neural system analysis have been thoroughly covered in numerous reviews and books.^12,17,23–25,49,53^

In classical electronics, oscillators are circuits that create a periodic output waveform. A source of regular oscillations, independent of external timing, is necessary in any instrument that initiates measurements of processes, and in devices where sustained oscillation or signal generation is required. Hence there is a large variety of electronic oscillators.^41,43^ These self-oscillatory devices and configurations are essential in a wide range of applications including radio frequency systems, where they enable stable carrier signal generation; signal generation circuits, where accurate and tunable frequency sources are required; wireless communication systems, where they facilitate the modulation and transmission of data over electromagnetic waves; and switch-mode power supplies, where controlled oscillations are used to efficiently convert and regulate electrical power. In each of these applications, the ability to sustain precise and continuous oscillation is critical for ensuring system reliability, performance, and efficiency. These standard systems provide valuable classification guidelines.

Since neuromorphic computation aims to obtain hardware oriented by brain properties, using intrinsic material properties and device physics to reproduce computational functionalities is a central strategy to replicate the bioneurological phenomena in artificial systems. Recently, a variety of emergent oscillatory technologies including threshold switching (TS) memristors and organic transistors, have significantly broadened the landscape for developing oscillators optimized for unconventional computing methods. The electrochemical oscillators have been amply studied over many years.^27,45–47,54^ S-type resistance in binary oxides memristors as VO_2_ and Nb_2_O_5_ associated with the metal-insulator transition (MIT)^32,34,55–58^ have been broadly researched for parallel computation.^1,33,36,37,59–66^ TS memristors^67–71^ offer fast, reversible switching behavior with low energy consumption, making them ideal for compact and programmable oscillator circuits. Similarly, organic transistors^72–78^ bring the advantages of mechanical flexibility, low-temperature processing, and material tunability, which open the door to unconventional form factors and customizable circuit behavior.

Here we adopt a general perspective of self-oscillators based on nonlinear dynamical systems. Rather than relying on alternative methods (Sec. [Sec sec4.5]) of local tests or transfer-function analyses at specific operating points,^35,79^ we focus on the direct formulation and analysis of the governing differential equations. This approach allows us to identify, a priori, the parametric regimes associated with stable equilibria, bifurcations, and self-sustained oscillations across a broad range of physical systems. By expressing classical oscillation concepts within a unified dynamical framework, we aim to provide intuitive and practical tools for researchers in materials science, chemistry, physics, and electronics to design and control neuron-like oscillatory devices.

By carefully tuning the parameters of a system of self-sustained oscillators, researchers can design neuromorphic circuits that not only mimic the spiking patterns observed in biological systems but also adapt to changing inputs in real time. A short introduction to spiking neural networks (SNN), oscillatory neural networks (ONN) and neural patterns in human cognitive activity is presented in Sec. [Sec sec2], to establish the context of the applications of self-sustained oscillators in unconventional computing paradigms. By mimicking the way the brain processes information, through sparse and event-driven activity, these systems excel at complex tasks such as pattern recognition, adaptive learning, and real-time decision-making.^4^ One example of coupled oscillators network for image edges detection is shown in Figure 3 (c, d, e).^21^ These systems are well-suited for applications in artificial intelligence, robotics, and edge computing, where low-power, high-performance solutions are essential.^80^

Additionally, we mention in Sec. [Sec sec2] oscillator-related topics that constitute active areas of research, as chemical and biological networks, and quantum and probabilistic computing. However, in this paper we do not address computational methods such as network training and computing algorithms, which is a large separate subject. Nevertheless, an important related topic, which is that of the memristor equations for the synapse applications,^81^ has been summarized in Appendix 1.

The general principles of self-sustained oscillators are presented in Sec. [Sec sec3]. Thereafter, two main methods to make oscillatory devices will be treated separately, following a standard approach.^82,83^ Secs. [Sec sec4]–[Sec sec6] focus on the class of oscillators based on a NDR. Sec. [Sec sec4] introduces two-equation models that show the basic structure of bifurcation and the main types of oscillations, and establishes the main oscillatory and bifurcation properties. Basic ingredients of these models are discussed in Appendix 1 and Appendix 2, and the analytical methods of the Hopf bifurcation are shown in Appendix 3. Sec. [Sec sec5] explains more complex oscillatory systems, involving a larger number of internal degrees of freedom, including the HH model for biological neurons. Sec. [Sec sec6] shows general methodologies for the analysis of oscillator properties. Another approach, presented in Sec. [Sec sec7], contrasts with the NDR-based models of the previous sections by focusing on oscillators constructed through explicit feedback loops involving active gain elements such as Op-Amps or transistors.^84^ This section classifies and analyses the most representative classical electronic feedback-based oscillators, and concludes by addressing hybrid architectures in which feedback is implemented electronically but sustained through the physical and chemical properties of functional device materials, as introduced in earlier sections.^43^ Together, these technologies enable the creation of tunable, scalable, and energy-efficient oscillators that are well-suited for integration into next-generation neuromorphic systems.

## 2 Oscillator Applications in Unconventional Computing

In this section, we provide a summary of different unconventional computing approaches that use neurons and oscillators as a key computational element, and we also discuss some elements of natural cognitive systems. Secs. [Sec sec2.5] and 2.6 present a brief perspective on the exciting possibilities in the field of quantum information science and probabilistic computation,^85^ although the rest of the paper will treat only classical systems. Secs. [Sec sec2.7] to 2.9 then address several key issues that must be carefully considered if ONNs are to become part of practical, real-world applications.

### 2.1 Spiking Neural Networks

An essential feature for encoding information in neuromorphic circuits is neuron spiking, which mimics the way biological neurons communicate through discrete action potentials. Neurons receive complex patterns of synaptic input and communicate by converting these inputs into sequences of electrical spikes. The reason for using spikes lies in their consistent shape.^12^ Since individual spike waveforms are largely uniform, the meaningful information is carried not in the shape of the spikes but in the timing between them, known as interspike intervals. This strategy of encoding information through timing, rather than waveform details, enhances the reliability and reproducibility of communication between neurons. Even though neural signals may undergo dispersion or attenuation, which can alter the shape of the spikes during transmission, the critical timing information remains intact, ensuring accurate signal interpretation across neurons.

In artificial neural networks (ANNs), neurons serve as the basic computational units that integrate inputs from various sources. SNNs are a type of neural network that more closely mimic the way the human brain processes information.^86^ Unlike traditional ANNs, which typically operate on continuous-valued signals and compute neuron outputs through weighted sums followed by static activation functions, SNNs encode and process information using discrete electrical pulses (spikes) distributed in time. Importantly, both ANNs and SNNs rely on weighted summation of inputs; however, in SNNs computation depends not only on synaptic weights but also on the precise timing of spike arrivals and the neuron’s temporal dynamics.

In SNNs, neurons remain inactive until the input they receive crosses a certain threshold, at which point they “fire” a spiking signal. This event-driven behavior reflects how biological neurons operate, enabling SNNs to capture both the timing and frequency of signals, which are crucial aspects of brain-like computation. The temporal coding makes SNNs inherently more energy-efficient and potentially faster for certain tasks, especially in hardware designed to exploit their asynchronous and sparse activity patterns. However, training SNNs is more complex due to the nondifferentiable nature of spike events, and they often require specialized learning rules or conversion from pretrained ANNs.

Currently, most neural networks are constructed using circuits based on CMOS (complementary metal-oxide-semiconductor) technology,^6,87–89^ which necessitates complex architectures and a large number of components to simulate spiking computational behavior, as shown in Figure 4b.^89–92^ The neurons in these networks rely on relatively large circuits composed of dozens of transistors and sizable integrating capacitors. As a result, replicating the behavior of biological neurons with current design and manufacturing technologies is challenging, leading to high power consumption and poor performance in emulating the human brain.

**Figure 4:**
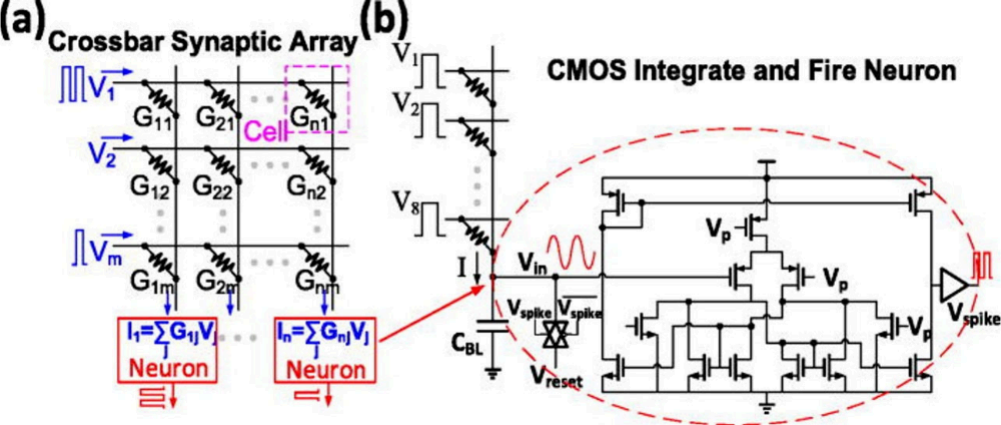
(a) The resistive crossbar array implements the synaptic network, and the neuron node at the end of the column integrates the weighted sum current from the array. (b) One example of the CMOS integrate-and-fire neuron design. The membrane voltage integrates and discharges after triggering the output spike. Reproduced with permission from ref 33. Copyright 2017 AIP Publishing.

The dominant neuron type in large-scale computational systems^93,94^ is the integrate-and-fire (IF) neuron that produces a spike when an accumulated voltage exceeds a threshold.^6^ We remark that IF methods do not involve a self-sustained oscillation, since they require a periodic input or an update mechanism for reset after firing, and they will not be discussed in this review.

The signal between neurons in ANNs is modulated by the weight of the synapse that interconnects the neurons, as outlined in Appendix 1. Resistive memory (RRAM) and memristors can emulate synaptic behavior by modulating conductance values, which correspond to synaptic weights.^95^ A practical approach to implementing these systems involves using synaptic memristors arranged in a crossbar chip architecture.^91,96^ This arrangement consists of perpendicular rows (word lines) and columns (bit lines), with memristive or RRAM devices located at their intersections. Each device at a cross-point stores a specific conductance value, as shown in Figure 4a, effectively representing the weight of a synapse in an artificial neural network.

The primary advantage of the crossbar structure lies in its ability to perform parallel vector-matrix multiplications directly in hardware, significantly enhancing computational efficiency and reducing energy consumption. Thus, crossbar arrays are particularly well-suited for implementing dense neural networks in edge computing and neuromorphic systems, where power and space are critical constraints. By enabling parallel processing and integrating memory with computation, it reduces data movement and enhances efficiency, making it ideal for large-scale neural network applications.

As shown in Figure 4a, when an input voltage vector is applied to the crossbar array, the resulting current through each synapse device (proportional to its conductance) sums at the end of each column.^33^ To represent synapses in neural network applications, memristors must have high-precision programmability and many distinct conductance levels to ensure consistent and accurate performance across both lab-made and factory-produced devices.^97,98^ The weighted sum current is integrated at the neuron node, which converts the analog signal into discrete spikes using a CMOS IF circuit. The spike count is directly related to the magnitude of the input current, serving as an analog-to-digital conversion. Even though conventional CMOS neuron circuits typically require dozens of transistors and occupy significant chip area, which exceeds the compact spacing of the crossbar array,^99^ such neuromorphic designs demonstrate efficient implementations of vector-matrix multiplication–an operation critical to neural network computation–while enabling compact and energy-efficient pattern recognition tasks. The operation of a 2 × 2 SNN is presented in Figure 5.^100^

**Figure 5:**
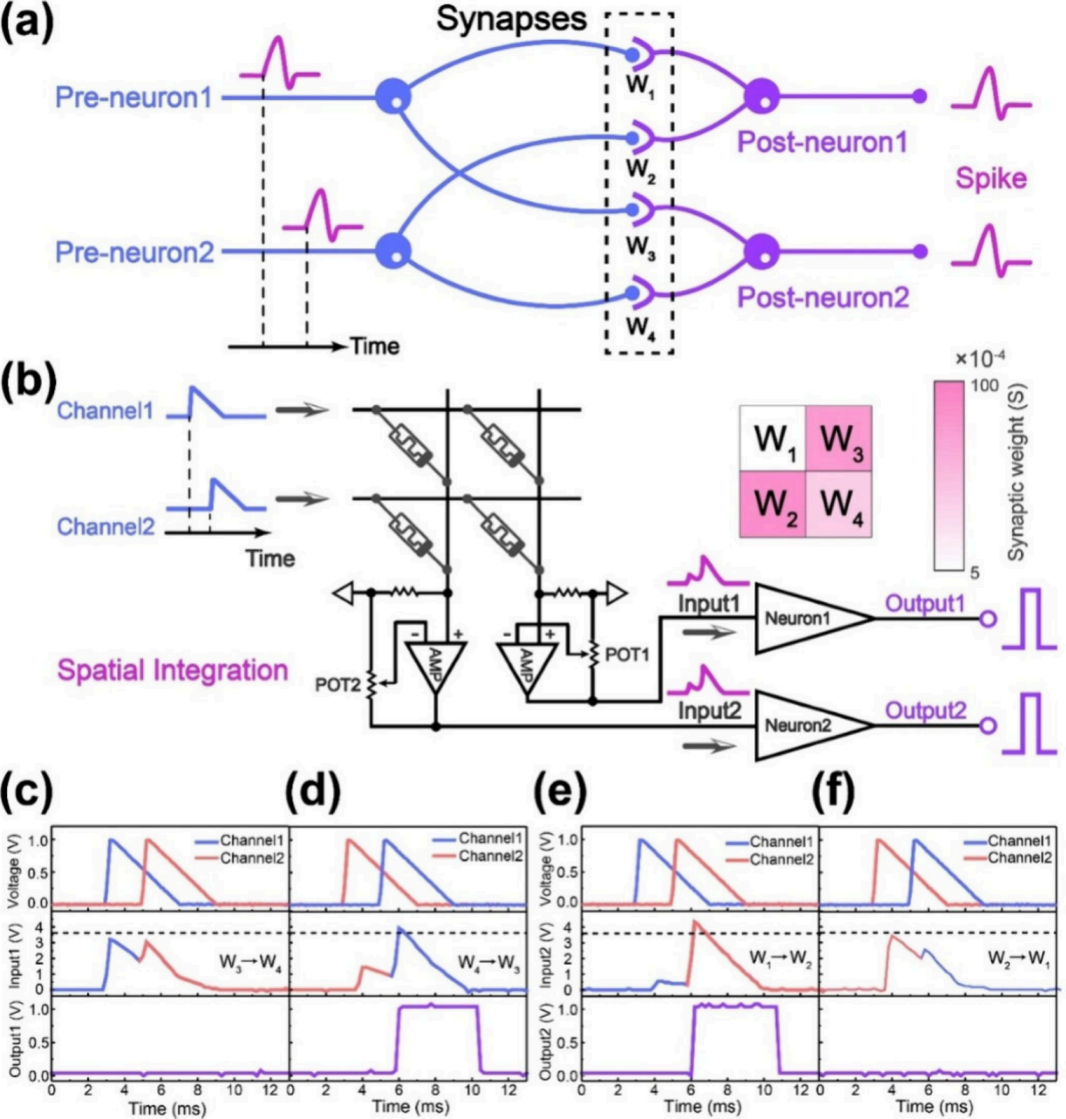
(a) Conceptual scheme of a spatiotemporal network which is composed of 2 Preneurons and 2 Postneurons. The Pre- and Postneurons connect to each other through 4 synapses, framing a simple spiking neural network (SNN). (b) Schematic diagram of the circuit realization of the spatiotemporal network in (a), and synaptic weights maps of the synaptic array. Neuron1 and Neuron2 are the simplified schematic diagrams of the LIF model. The stimulus signals (voltage signals come from Channel1 and Channel2) are integrated on the synaptic array. Then the currents flowing through the synaptic array are amplified by the amplifiers and converted into voltages to stimulate the neurons. (c) Output of Neuron1 in an input sequence of Channel1 - Channel2. (d) Output of Neuron1 in an input sequence of Channel2 - Channel1. (e) Output of Neuron2 in an input sequence of Channel1 - Channel2. (f) Output of Neuron2 in an input sequence of Channel2 - Channel1. Reproduced with permission from ref 100. Copyright 2020 Elsevier.

S-type NDR in binary oxide memristors as VO_2_ and Nb_2_O_5_ associated with the MIT^32,34,55–58^ have been applied for parallel computation, as shown in Figure 3.^1,33,36,37,59–66^ A MIT oscillator neuron can be integrated with RRAM arrays. This device is intended to replace the more complex and larger CMOS-based neuron circuits traditionally used in neuromorphic systems. Circuit-level simulations have shown that this design has clear advantages in terms of smaller area, lower latency, reduced energy consumption, and less leakage power.

Figure 6 shows the switching characteristics of a niobium oxide (NbO_*x*_) neuron device. This material shows oscillatory properties, as mentioned and discussed in detail in Sec. [Sec sec4.7]. Importantly, the frequency of these oscillations is related to the strength of the input current, which represents the weighted sum of signals in the neural network. This allows the MIT device to perform the same function as a spiking neuron in a much more efficient and compact way.^33^

**Figure 6:**
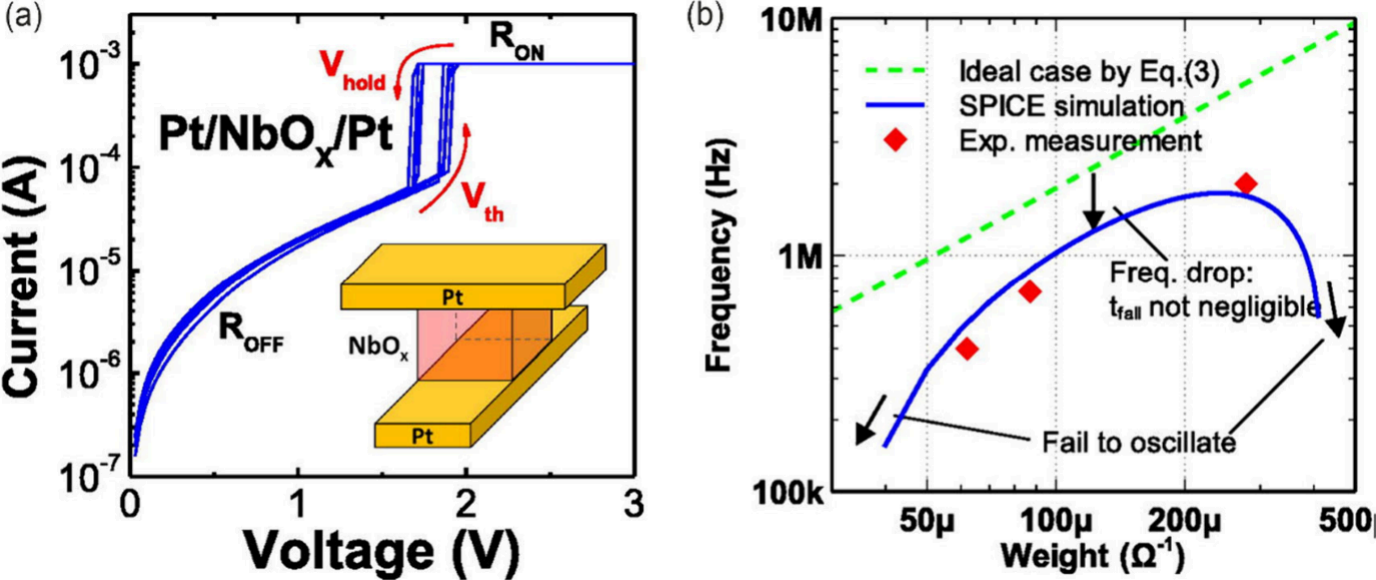
(a) Measured current-voltage TS characteristics of the Pt/NbO_*x*_/Pt device. The inset shows the schematic of the fabricated Pt/NbO_*x*_/Pt device. (b) Oscillation frequency as a function of synaptic weight (1/R_L_). The SPICE result shows good consistency with the measured frequencies. Oscillation failure occurs when the weight is too large or too small. Reproduced with permission from ref 33. Copyright 2017 AIP Publishing.

### 2.2 Oscillatory Neural Networks

A recent approach to solving difficult problems involves using a network of electrical oscillators. Oscillator-based computation (OBC), which leverages self-sustained oscillators for processing information, is gaining widespread attention due to its potential to offer energy-efficient, parallel, and neuromorphic alternatives to traditional computing architectures.^39,40,101^ Information is processed through the phase relationships between oscillators, as illustrated in Figure 3, allowing ONNs to perform analog computation in the phase domain–an approach well-suited to tasks such as pattern recognition and clustering,^1,36,37,59–66^ as shown in Figure 7.^31^ In particular, Figure 7(a,b) illustrates ONNs in which all oscillators are mutually coupled, analogous to a Hopfield network. However, such all-to-all connectivity is not a general requirement. The manner in which oscillators are interconnected—whether through reciprocal or nonreciprocal couplings—defines the network topology, which exerts a profound influence on the resulting dynamics and, consequently, on the computational process.

**Figure 7:**
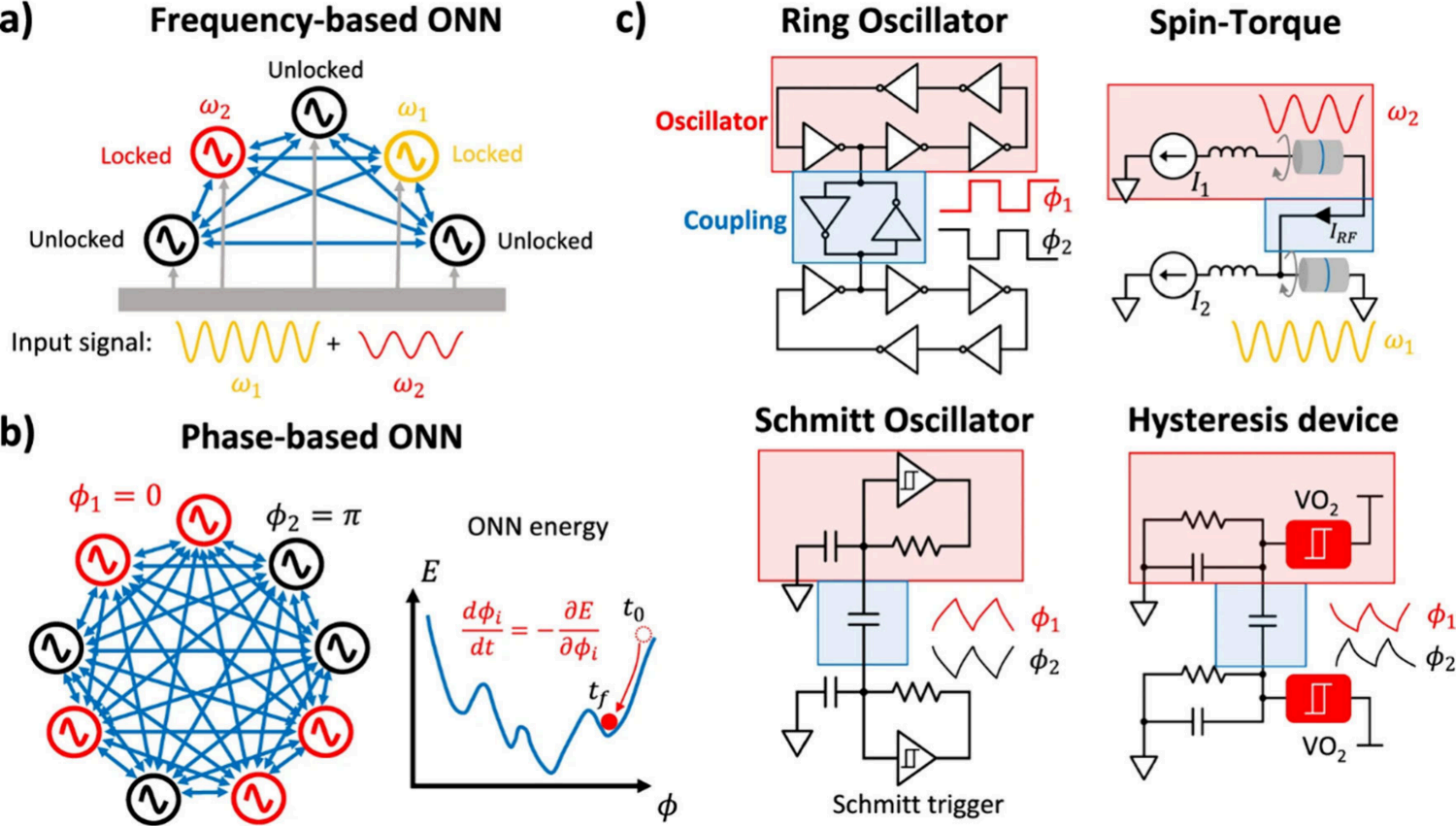
(a) Illustration of a frequency-based ONN. The input consists of frequency-dependent signals fed into the ONN. The latter can have oscillators locking to a common frequency and providing the computational result. Labels of locked and unlocked are shown next to the oscillators. (b) Schematic of a phase-based ONN. The oscillators have a uniform frequency and evolve in the phase domain, minimizing a kind of Hopfield energy. (c) Two coupled oscillators implemented with various technologies. Ring oscillators can be coupled by back-to-back inverters or using transmission gates. Relaxation oscillators consist of a hysteresis device that charges/discharges a load capacitor, producing analog oscillations. The hysteresis component can be implemented by a Schmitt trigger or beyond-CMOS devices that have a NDR region in their I-V characteristic. Reproduced from ref 39. Available under a CC-BY 4.0 license. Copyright 2024 The Author(s). Published by Springer Nature.

At this point, it is important to emphasize that the focus on stable oscillatory regimes in this review reflects a deliberate design choice rather than a fundamental limitation of ONNs. While many ONN implementations exploit well-defined phase-locked or frequency-locked states for clarity and controllability, self-sustained oscillatory devices and their networks can access a much richer dynamical landscape arising from device physics and collective interactions.^24,102^ These include multistability, complex basin structures, and high-dimensional or spatiotemporal dynamics. A detailed treatment of such phenomena—including chaotic behavior and fractal basin boundaries—is beyond the scope of the present review; however, these features may become computationally beneficial if appropriate control, feedback stabilization, and topology-aware design strategies are developed. We therefore highlight these aspects as important directions for future research.

A key idea in this context is the use of an energy function (also called a Lyapunov function, see Appendix 2) to describe how the system behaves over time. It was introduced by Hopfield in 1982,^103^ for developing models of associative memory. According to this concept, the neural network is seen as a dynamical system with many stable states, or attractors, each representing a stored memory. The concept involves setting up a memory network during the learning phase so that it forms a set of attractors (stable states) each linked to a specific output. In this system, when a particular input is given, it places the network in a starting state within the “basin of attraction” of one of these attractors. The network then naturally evolves toward that attractor, resulting in the correct, predefined output. This physical process allows the system to efficiently explore solution spaces and converge on optimal or near-optimal solutions without exhaustive computation. In this regard it is important to emphasize that the oscillatory systems considered in this review are inherently non-Hamiltonian. In purely Hamiltonian systems, energy is conserved and phase-space trajectories do not converge toward minima of an energy function, as no attractors exist.^24^ As a result, Hamiltonian dynamics alone cannot support relaxation toward stable states or energy-minimizing solutions. Nevertheless, many practical oscillatory circuits can be viewed as perturbations of underlying Hamiltonian systems, which naturally support oscillatory motion but lack amplitude stabilization. The introduction of weak nonlinear dissipation or active driving breaks energy conservation and can lead, via bifurcations, to self-sustained oscillations with well-defined amplitude and tunable frequency. While the present review focuses on dissipative oscillators operating in such regimes, the Hamiltonian viewpoint provides an important conceptual foundation.

ONNs have shown significant promise in addressing a range of challenging computational tasks.^38–40^ One of their standout capabilities lies in solving complex combinatorial optimization problems.^104–106^ ONNs are also well-suited for pattern recognition and associative memory applications.^21,107^ They have been successfully used in tasks involving image and signal processing, where the network can identify, recall, and reconstruct patterns based on partial or noisy inputs–functioning much like a content-addressable memory. In addition to their computational versatility, ONNs are highly power-efficient and operate at impressive speeds. They can reach stable solutions in as few as 25 oscillation cycles, significantly outperforming conventional CMOS-based systems in both energy consumption and latency. These features make them particularly attractive for deployment in edge computing and real-time applications, where resources are limited and quick responses are critical.

### 2.3 Neural Patterns for Biological Information Processing

As shown in Figure 2 the basic process a neuron utilizes for communicating with their counterparts is the spike, which is a brief but essential electrical impulse also known as action potential. Natural neuron spiking exhibits several distinct characteristics that contribute to the complexity and efficiency of biological information processing.^49^ When a neuron fires, it generates an action potential that lasts only a few milliseconds and is followed by a refractory period, which prevents immediate reactivation. Another key characteristic is bursting, consisting of the generation of multiple spikes in rapid succession. This phenomenon is crucial for sensory perception, motor control, and cognitive functions, often emerging from intrinsic ion channel dynamics or network-level interactions.^108^ In contrast, spike-frequency adaptation describes how a neuron’s firing rate decreases over time in response to a sustained stimulus.^109^ All these phenomena contribute to the complexity and efficiency of biological information processing.^49^

Particularly, the mind dynamics, or human cognitive activity, can be understood as the interaction of sequential mental processes organized hierarchically, where metastable states–transient, structured activity patterns–form the foundation of perception, decision-making, and behavior.^110^ The concept of sequential order in mental function that was first introduced in the context of behavior, has since become a foundational idea in neuroscience,^111^ providing insight into how the brain generates coherent streams of thought and action. This dynamic organization mirrors the hierarchical structure of the brain’s functional networks,^112^ where lower-level sensory processes feed into higher-order integrative networks.

Oscillatory neural circuits, particularly central pattern generators, rely on both intrinsic rhythmogenic mechanisms and pattern-forming processes. A fundamental question in the study of these networks is how the intrinsic activity of individual neurons is influenced by synaptic dynamics and overall network organization, and how coordinated output emerges from the collective activity of network components.^113^ These questions are further discussed in Sec. [Sec sec6.3].

Over the past three decades, two main mechanisms for generating low-frequency oscillations have been identified. The first involves pacemaker-driven rhythmogenesis, where a single neuron or a group of synchronized neurons generates bursting activity due to their intrinsic cellular properties.^113^ The rest of the network organizes the timing and phase relationships among principal neurons or groups to produce a coherent spatiotemporal output. The second mechanism, network-based rhythmogenesis, does not rely on neurons with intrinsic rhythmicity. Instead, rhythmic activity arises from the dynamic interactions between tonically spiking neurons connected through excitatory and inhibitory synapses. These interactions can lead to rhythmic bursting through synapse-mediated modulational instabilities. However, when both intrinsic bursting and network-based mechanisms are present within a single system, the principles governing their interaction remain largely unclear, particularly regarding how such hybrid systems yield robust, flexible outputs^114,115^ and highlighting a key area for further research in understanding complex oscillatory dynamics in neural networks.

Neuromorphic architectures that integrate intrinsic rhythmogenic and synaptic mechanisms, such as bursting, can more faithfully simulate the hierarchical and sequential nature of mental processes. These advanced systems are not only powerful tools for modeling brain function but also foundational for the development of adaptive, intelligent technologies capable of performing cognitive tasks in a brain-like manner.^7,116^ Replicating such complex spiking behaviors in artificial systems is a central goal of neuromorphic engineering, as we discuss in Sec. [Sec sec5.1].

### 2.4 Chemical and Biochemical Networks

Interestingly, OBC can be extended to nonelectrical, chemical and biological systems. Chemical oscillators, such as the Belousov-Zhabotinsky (BZ) reaction, exhibit periodic or nonperiodic changes in chemical concentration.^117–120^ Their behavior is governed by nonlinear reaction kinetics and diffusion, often modeled by reaction-diffusion equations or systems of ordinary differential equations.^23^ These systems can be harnessed to perform logical operations, memory storage, and pattern recognition by encoding it in oscillation amplitudes, phases, frequencies, or patterns of chemical concentration waves.^121–123^ Many vital physiological processes, such as the regulation of the p53 protein, cell cycles, and circadian rhythms, rely on oscillatory signals.^124^ These processes are controlled by complex circuits that allow organisms to fine-tune parameters like period, amplitude, and phase. However, synthetic oscillations in engineered networks are harder to control with the same precision.^125,126^ Achieving stable and accurate control of oscillation is a significant challenge.^127,128^ Importantly, synthetic circuits that independently control amplitude and period have wide-ranging applications, including frequency analysis, signal processing, and the design of advanced gene circuits, such as biosensors and therapeutic molecule delivery systems.

### 2.5 Quantum Oscillators for Information Science

Quantum states define the behavior of the microscopic world, embodying the fundamental wave-particle duality at the heart of quantum theory. Quantum computing represents a contemporary evolution of foundational scientific principles established over a century ago. Of particular interest is how the probabilistic nature of quantum states gives rise to interference effects and other exotic correlations, which manifest in measurement outcomes. The principles of quantum mechanics are characterized by two key phenomena, superposition and entanglement, that form the cornerstone of quantum computation.^129^ Superposition refers to the ability of a quantum particle to exist in multiple states simultaneously, while entanglement describes a unique correlation between particles, such that the state of one instantly affects the other, regardless of the distance separating them. These general concepts give rise to different tools: the qubits, the fundamental units of quantum information, and quantum gates and circuits.^130^

Quantum computing offers several transformative advantages that address future technological and scientific challenges. Its most significant benefit lies in its ability to process and analyze vast amounts of data exponentially faster than classical computers, thanks to principles like superposition and entanglement. This enhanced computational power has the potential to revolutionize fields such as cryptography, by breaking traditional encryption schemes and enabling new, quantum-secure protocols. In materials science and chemistry, quantum computers can simulate complex molecular interactions with high accuracy, accelerating drug discovery and the development of new materials. Additionally, quantum algorithms hold promise for optimizing complex systems in logistics, finance, and AI. As these capabilities mature, quantum computing is expected to become a critical tool in solving problems that are currently intractable for classical machines.

In quantum computing, several types of physical oscillators are employed to create and manipulate qubits, with the most common being superconducting resonators, trapped ions, and optical or microwave cavities. These oscillators serve as the fundamental building blocks for encoding quantum information and enabling controlled interactions. Superconducting resonators, used in platforms like transmon qubits, are based on microwave-frequency circuits that exhibit quantized energy levels, Figure 8.^131,132^ Trapped ions use the quantized vibrational modes of ions confined in electromagnetic fields, allowing precise control over their quantum states.^133^ Optical and microwave cavities, often used in cavity quantum electrodynamics, enhance the interaction between qubits and photons, facilitating quantum state manipulation and readout.^134,135^ In all cases, these oscillators play a crucial role in initializing, controlling, and measuring qubit states with high fidelity, making them essential components of quantum hardware.

**Figure 8:**
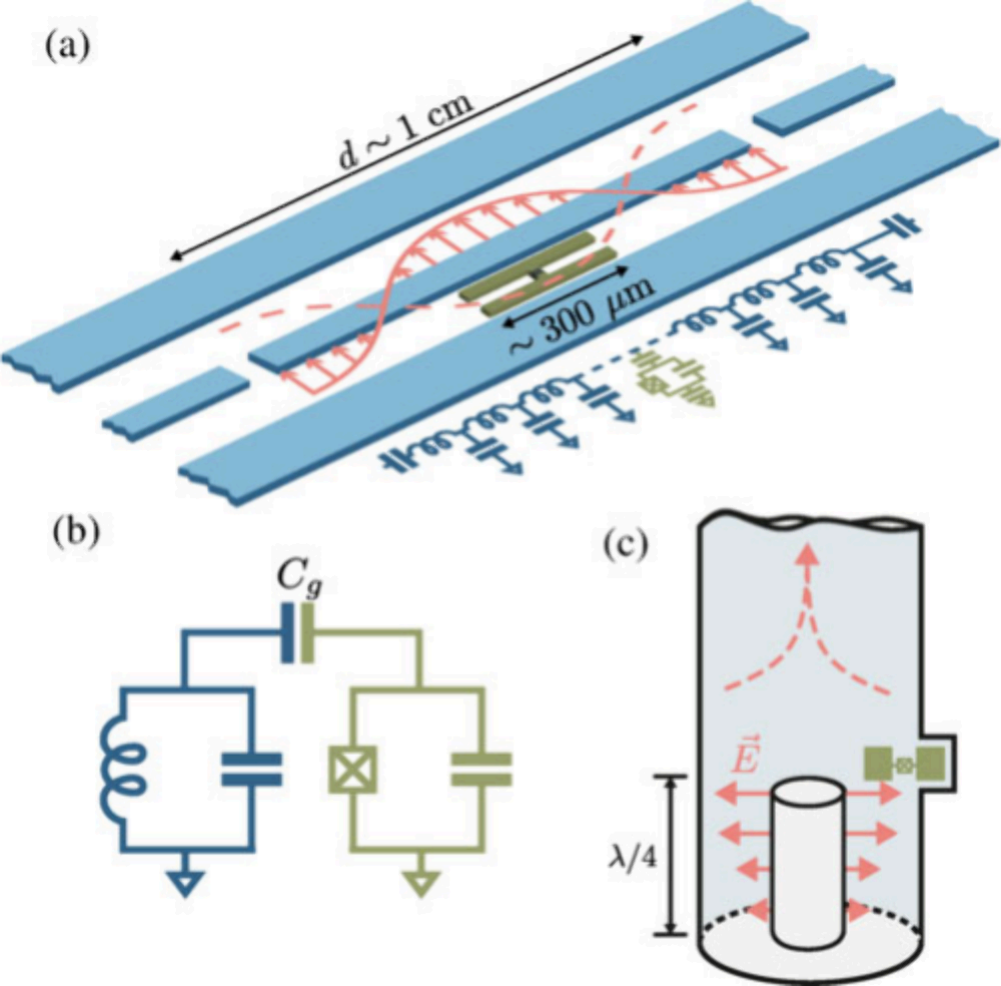
Schematic representation of a transmon qubit (green) coupled to (a) a 1D transmission-line resonator, (b) a lumped-element LC circuit, and (c) a 3D coaxial cavity. (a) Adapted with permission from ref 135. Copyright 2004 American Physical Society. (b) Reproduced with permission from ref 140. Copyright 2021 American Physical Society. (c) Adapted with permission from ref 132. Copyright 2016 American Physical Society.

Building on the role of oscillators in quantum systems, circuit realizations of topological physics have emerged as a promising approach for simulating and exploring exotic quantum phenomena in a controlled setting.^136^ In this context, electrical and microwave circuits are designed to mimic the behavior of topological phases of matter, such as topological insulators and superconductors.^137^ These circuits use arrays of resonators or inductors and capacitors arranged in specific geometries to emulate the Hamiltonians of topological systems.^138,139^ By carefully engineering coupling strengths and boundary conditions, researchers can observe protected edge modes and topological invariants analogous to those found in condensed matter systems.^140^ This platform provides a versatile and highly tunable environment for investigating the robustness of topological states against disorder and perturbations, which is essential for future applications in fault-tolerant quantum computing. Circuit realizations not only offer insight into fundamental physics but also pave the way toward the development of topologically protected qubits, which promise enhanced stability and error resistance in quantum processors.

### 2.6 Quantum-like Systems and Probabilistic Computing

A well-known obstacle for the achievement of mainstream quantum computing is the inherent fragility of quantum states, especially the most intriguing ones, which are easily disrupted by environmental noise and fluctuations, a process known as decoherence. Probabilistic computing using p-bits is an emerging computational paradigm that bridges the gap between classical and quantum computing by leveraging randomness in a controlled and tunable manner.^141^ A p-bit (probabilistic bit) is a classical unit that fluctuates between binary states (0 and 1) with a probability that can be biased by an input signal. Unlike deterministic bits in classical computers or qubits in quantum systems, p-bits are inherently stochastic, yet operate at room temperature and are much easier to implement using conventional technologies such as CMOS, spintronics, or magnetic tunnel junctions.^142^

P-bits are particularly suited for solving optimization problems, probabilistic inference, and sampling tasks commonly encountered in fields like machine learning, neural networks, and combinatorial optimization. They can implement Boltzmann machines, Ising models, and Bayesian networks, offering a hardware-friendly approach to these otherwise computationally expensive algorithms.^142,143^

Oscillators play a crucial role in probabilistic p-bit computing, primarily as stochastic signal generators and timing/control elements. In this context, oscillators–often implemented using thermal noise sources, ring oscillators, or magnetic dynamics–are used to produce fluctuating signals that drive p-bits to randomly switch between 0 and 1 states in a controlled probabilistic manner.^144^

Binary oxide oscillators provide a convenient framework for establishing p-bits computational networks. To realize compact p-bit elements, TS devices based on NbO_*x*_ have been explored, leveraging the material’s MIT behavior.^66,146^ These devices exhibit a sharp, reversible change in resistance when a threshold voltage is applied, enabling stochastic switching characteristics suitable for probabilistic computing. The inherent nonlinearity and volatility of NbO_*x*_-based TS devices make them promising candidates for energy-efficient, room-temperature p-bit implementations. P-bit operation was experimentally demonstrated by inducing instability in SiO_*x*_-based TS, as shown in Figure 9.^145^ While SiO_*x*_ normally shows stable TS and voltage oscillations, the introduction of a Ti scavenging layer generated oxygen vacancies, leading to probabilistic oscillations suitable for p-bit behavior.^147^

**Figure 9:**
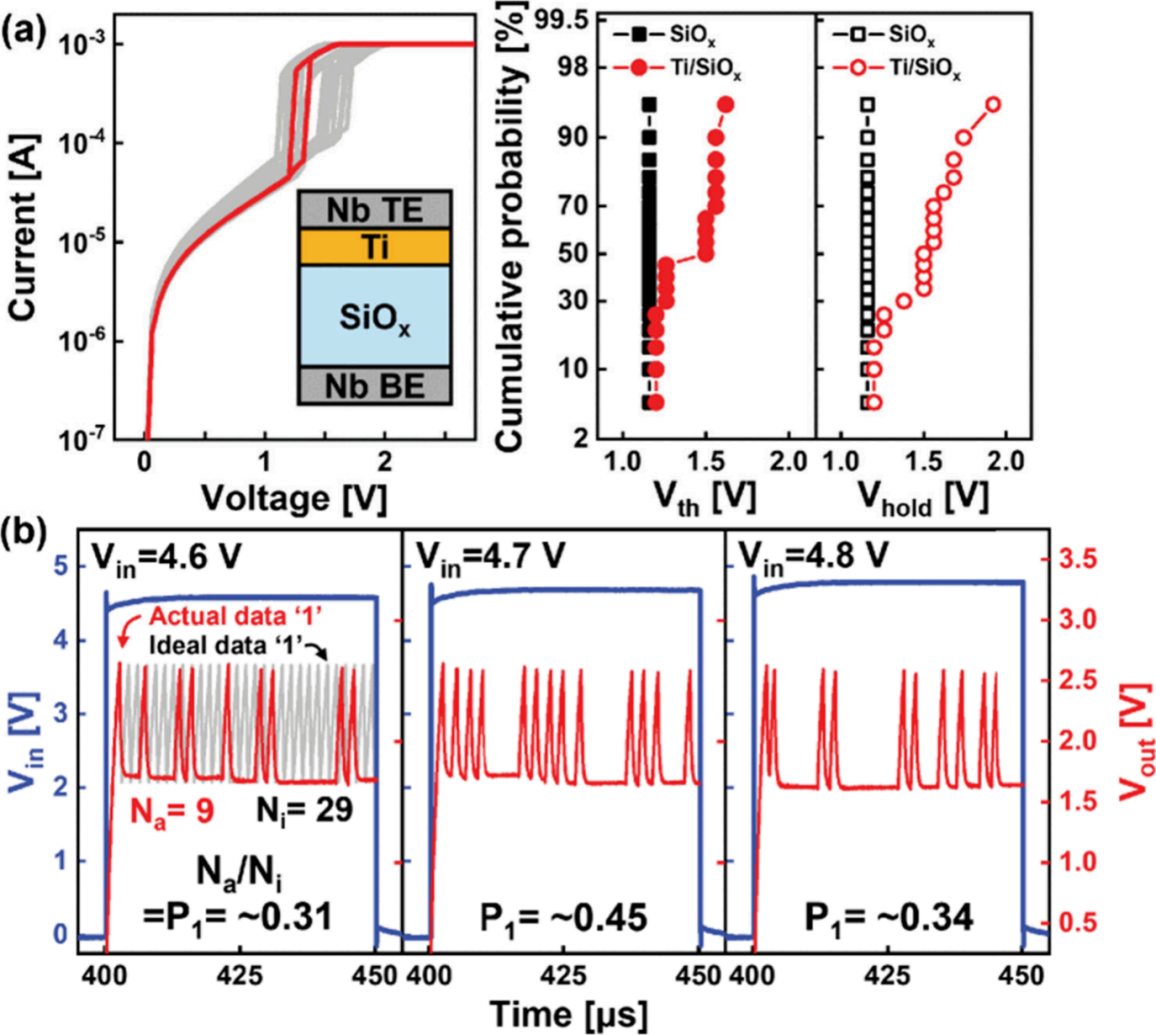
(a) Current-voltage characteristics and cycle-to-cycle distribution of switching voltages in the Nb/Ti_2_/SiO_*x*_/Nb stack. (b) Probabilistic V_out_ oscillation was achieved at the given V_in_. Reproduced with permission from ref 145. Copyright 2024 IEEE.

It has been remarked that quantum-like probability laws, including interference effects, can manifest in classical systems.^148^ Special networks of coupled oscillators can be constructed using topologies based on expander graphs–sparse yet highly connected structures known for their efficient information propagation and robustness. In such networks, the collective dynamics of the oscillators give rise to discrete emergent states, even though each individual oscillator evolves continuously over time. A particularly interesting property of these systems is their ability to generate an emergent state that represents a superposition of two distinct states, each corresponding to a different configuration of coupled oscillator subnetworks.^149^

Recent studies have demonstrated that classical electric circuits, due to the mathematical correspondence between the circuit Laplacian and Schrödinger’s equation, can effectively simulate topological physics and quantum systems.^150,151^ These circuits have also been used to implement quantum-inspired information processing, enabling exploration of quantum-like behaviors using classical hardware.^147^

### 2.7 Energy Consumption and Efficiency in Oscillatory Neuromorphic Computing

Energy efficiency is a central motivation for neuromorphic computing, yet its quantification in oscillatory systems requires careful consideration. Unlike conventional digital circuits, where energy is typically associated with discrete logic operations, oscillatory neuromorphic elements operate in continuous time, and their computational function emerges from dynamical processes such as spiking, phase evolution, and synchronization. As a result, energy consumption must be evaluated in terms of physical events—such as oscillation cycles, spikes, or coupling interactions—rather than Boolean operations.

In biological neurons, the energetic cost of information processing is often estimated through metabolic expenditure, with early studies suggesting that the generation of a single action potential consumes on the order of several picojoules of energy. While such estimates vary and are difficult to associate with specific cognitive functions in vivo, they provide a useful reference scale for artificial systems that aim to emulate neuronal dynamics.^152,153^ In neuromorphic hardware, by contrast, circuit-level models allow energy consumption to be estimated more directly from electrical variables, offering a clearer link between device physics and computational cost.

For a single self-sustained oscillator, the average power consumption can be expressed as, *P* = ⟨*V*(*t*)*I*(*t*)⟩, where the brackets denote a time average over one or multiple oscillation periods. The energy associated with a computational event, such as a spike or oscillation cycle, is then given by *E* = *P*/*f*, where *f* is the oscillation frequency. This formulation naturally accommodates different operational modes, including relaxation oscillations, spiking regimes, and quasi-harmonic oscillations, and provides a common metric for comparing diverse oscillator implementations.

Within this framework, different classes of oscillatory computational devices exhibit distinct energy-function trade-offs. TS and NDR-based oscillators often rely on localized high-field or thermal processes, which can increase instantaneous power dissipation but may still yield low energy per cycle when operated at modest frequencies or near bifurcation thresholds. Feedback-based oscillator circuits, including transistor-based implementations, typically offer greater control over operating point and frequency, at the expense of additional static power associated with active gain elements. Organic electrochemical oscillators, by contrast, often operate at lower voltages and currents, benefiting from ionic-electronic coupling, though their slower dynamics can lead to higher energy per computational event depending on the application.

Importantly, energy efficiency in oscillatory computational systems is strongly task-dependent.^154–156^ Computation based on synchronization or phase locking can occur over many cycles with minimal incremental energy cost, while transient events such as phase transitions or spike generation may dominate consumption. At the network level, collective dynamics can further reduce energy expenditure by distributing computation across coupled oscillators, rather than requiring repeated switching of individual elements.

Rather than providing a single figure of merit, this perspective emphasizes that meaningful energy comparisons must account for operating regime, function, and time scale. By linking energy consumption directly to oscillatory dynamics, neuromorphic circuit models enable rational estimation of the energetic cost of encoding, transmitting, and processing information, offering a pathway to compare emerging technologies on physically grounded terms.

### 2.8 Programmability and Functional Reconfiguration of Self-Sustained Oscillators

Unlike conventional digital circuits, oscillatory neuromorphic elements do not perform computation through sequential logic or arithmetic operations. Instead, computation emerges from the collective dynamics of interacting oscillators. Consider, for example, a fully connected network of oscillators—analogous to a Hopfield network—operating as an associative memory. When an input is applied, for instance in the form of a set of voltage amplitudes assigned to individual oscillators, the system is initialized in a specific configuration characterized by defined phase differences among the oscillators. As the network evolves, its dynamics drive it toward the nearest minimum of an effective energy landscape, corresponding to a new distribution of phase relationships. At this point, the system can be said to have completed the computation. For this process to occur meaningfully, the network must be appropriately trained by encoding the desired energy minima. This encoding is achieved by tuning the intrinsic parameters of the oscillators or those of the coupling elements, which may include resistors, capacitors, or combinations thereof.

If a test pattern sufficiently resembles one of the stored patterns, the oscillators collectively evolve toward a synchronized state, either in phase (phase-shift keying) or in frequency (frequency-shift keying), which is interpreted as successful recognition. Conversely, the absence of synchronization indicates a failure of recognition.^157^

To date, such encoding has often been implemented using off-chip passive components, for example through programmable resistors or external capacitors.^157^ However, achieving oscillatory neural networks with intrinsic functional reconfiguration and programmability—without reliance on external elements—is crucial for realizing compact, scalable, and energy-efficient neuromorphic hardware.

From a physical perspective, it is useful to distinguish between short-term (volatile) and long-term (non-volatile or quasi-non-volatile) modes of programmability. Short-term modulation typically arises from reversible changes in operating conditions, including bias voltage or current, load resistance or capacitance, temperature, and transient ionic or electronic redistribution. Such mechanisms allow continuous tuning of oscillatory characteristics and enable rapid reconfiguration of spiking behavior, for example by adjusting oscillation frequency or phase response. These modes are particularly relevant for adaptive signal processing and real-time computation, where fast and reversible control is required.

Long-term programmability, by contrast, originates from persistent changes in the internal state of the active medium. In many self-sustained oscillators, especially those based on correlated oxides, electrochemical systems, or mixed ionic-electronic conductors, electrical stimulation can induce lasting modifications such as ionic migration, redox state changes, defect redistribution, or local structural transformations. These processes alter key parameters governing oscillation—such as threshold voltage, NDR strength, or effective capacitance—thereby enabling stable shifts between dynamical regimes. In this way, oscillators can be programmed to operate at different frequencies, spike patterns, or stability conditions over extended time scales.

Representative examples include oxide-based oscillators in which pulsed electrical stressing modifies oxygen vacancy profiles, leading to reproducible and long-lived changes in oscillation characteristics. Recent work on vanadium oxide electrochemical memory systems has demonstrated how controlled ionic motion can simultaneously enable reconfigurability and long-term retention, allowing oscillatory behavior to be programmed and maintained without continuous power input.^158^ Similar principles could be applied to organic electrochemical neurons, where electrochemical doping levels act as slow internal variables that tune excitability and spiking dynamics. Other approaches use emerging ReRAM as coupling elements between the oscillators.^159^

Beyond the tuning of individual oscillator and coupling parameters, network topology plays a central role in determining how information is encoded and processed in oscillatory neural networks.^160,161^ The connectivity pattern—whether fully connected, sparse, modular, or hierarchical—directly shapes the effective energy landscape and constrains the set of accessible collective states and their stability. Consequently, different topologies give rise to distinct synchronization regimes and convergence properties,^162,163^ ranging from global synchronization and associative memory behavior to clustered, partial, or metastable phase states.

Importantly, coupling topology constitutes an additional degree of freedom for programmability. By tailoring the connectivity—either through circuit design or reconfigurable coupling elements—the same oscillatory units can implement different encoding schemes and computational functions. Encoding in ONNs therefore emerges from the combined interplay of oscillator parameters, coupling strengths, and network topology, rather than from any single element in isolation.

### 2.9 Long-Term Stability and Reliability of Oscillatory Neuromorphic Devices

Long-term stability and reliability represent critical challenges for any neuromorphic hardware platform, particularly when nonlinear and far-from-equilibrium phenomena are exploited. In biological systems, sustained functionality is ensured through continuous repair, redundancy, and adaptive regulation across multiple spatial and temporal scales. Artificial oscillatory systems lack direct analogues of cellular regeneration; however, they can achieve robustness through dynamical, material, and architectural mechanisms that differ fundamentally from those used in conventional digital electronics.

At the device level, many self-sustained oscillators operate close to the onset of instability, such as near a Hopf bifurcation or a TS condition. However, stable oscillations correspond to limit cycles, which are inherently self-limiting dynamical states.^24^ Unlike uncontrolled runaway processes, limit cycles constrain current, voltage, and temperature excursions through nonlinear feedback, thereby mitigating catastrophic failure modes associated with excessive Joule heating. Therefore, in TS memristors and S-type NDR devices, oscillatory operation can in principle be more stable than static DC biasing, which is known to promote thermal breakdown in conventional semiconductor devices.

Material-specific degradation mechanisms nonetheless remain relevant. In oxide-based TS devices, long-term operation may be affected by filament overgrowth, irreversible stoichiometric changes, or local thermal damage.^164^ In particularly VO_2_-based devices are especially susceptible to degradation due to the granular nature of the material, which can promote localized damage. To address these issues, several stabilization strategies have been explored, including alternative fabrication routes^165^ and the introduction of additional layers (such as HfO_2_) combined with tailored annealing treatments.^37^ Similar multilayer approaches have also been applied to NbO_*x*_-based devices.^166^ Despite these advances, further research is still required to fully mitigate long-term degradation.

An alternative strategy involves identifying materials that exhibit nonlinear switching behavior without undergoing permanent structural transformations. In this context, perovskite films based on TbMnO_3_ have been shown to provide stable oscillatory behavior without requiring an electroforming step.^167^ While these materials offer promising stability advantages, their energy consumption must still be reduced to meet the requirements of practical neuromorphic applications.

Similarly, organic electrochemical transistor (OECT) oscillators face challenges related to electrochemical side reactions, ion trapping, and material instability, particularly in n-type conductors. Recent materials strategies—including improved encapsulation, electrolyte engineering, redox-stable polymers, and molecular design to suppress parasitic reactions—have demonstrated significant improvements in operational stability.^168,169^ While such approaches may alter oscillation parameters such as frequency, amplitude, or response time, these changes can often be incorporated into the dynamical design of the oscillator rather than viewed as failure modes.

A distinctive advantage of oscillatory neuromorphic systems is the possibility of active feedback stabilization.^170,171^ Electrical monitoring of oscillation characteristics (e.g., frequency drift, amplitude decay, or phase noise) provides real-time indicators of device state and degradation. Adaptive biasing, duty-cycle control, or coupling modulation can then be employed to compensate for slow material evolution, effectively implementing a form of electronic homeostasis. This approach parallels biological regulation mechanisms, where function is preserved through feedback rather than static structural perfection.

Finally, long-term reliability must be considered at the network level, rather than solely at the level of individual devices. ONNs exhibit intrinsic robustness arising from redundancy, collective synchronization, and metastability. The loss or degradation of individual oscillators does not necessarily compromise system-level function, as computation is distributed across phase relationships and transient coordination patterns. Moreover, the stochastic failure of oscillatory cycles in SiO_*x*_ based devices is currently being explored as a computational resource for probabilistic computing paradigms.^147^ In this sense, oscillatory neuromorphic architectures more closely resemble biological neural systems than traditional deterministic circuits, where single-component failure can be catastrophic.

Taken together, these considerations suggest that the long-term viability of oscillatory neuromorphic devices will rely not only on advances in materials stability, but also on exploiting the self-regulating nature of nonlinear dynamics, feedback control, and network-level resilience. These features offer a pathway toward reliable neuromorphic hardware that aligns with the adaptive principles observed in biological intelligence.

## 3 Principles and Types of Classical Self-Sustained Electronic Oscillators

### 3.1 NDR and Feedback

The principles of self-sustained oscillators were established long ago using basic systems such as mechanical resonators, electrical circuits and vacuum tubes.^17,18^ These methods were expanded with the formalism of nonlinear dynamics in electronic and neuroscience applications. There are quite developed methods to establish oscillatory devices.^16,83^ We start our exploration of self-sustained oscillators taking the principles of the area of electronic oscillators.

The general structure of an oscillatory circuit is shown in Figure 10a. A main piece is the *LC* parallel connection called the “tank circuit”. For the ideal circuit with no losses (*R*_*L*_ = 0) the impedance of the tank at the angular frequency ω is

1 $$Z_{tc}\left(\omega \right)=\frac{i}{\frac{1}{L_{0}\omega }-C_{0}\omega }$$

**Figure 10:**
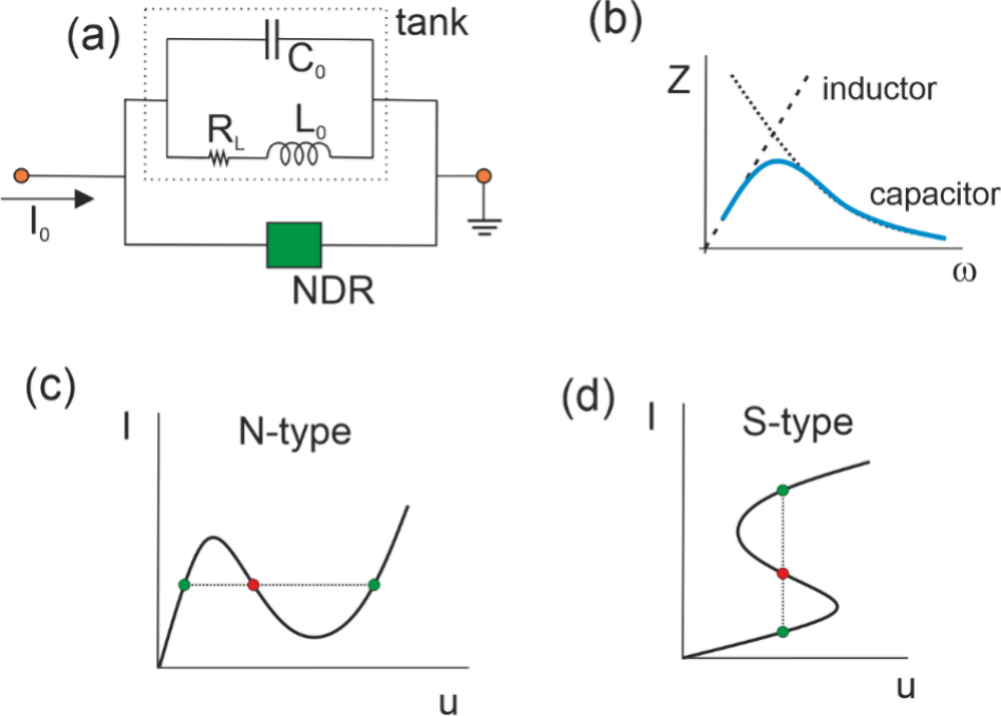
(a) Oscillatory circuit including the tank *LC* circuit with resistance loss *R*_*L*_, and a NDR element (green block), with an injected current *I*_0_. (b) Modulus of the impedance for a LC circuit as a function of frequency. (c, d) Types of NDR. The current-voltage curve of the green element is shown. Bistability is indicated by stable green points, and an unstable red point.

When the circuit is driven by an external AC source, a resonance occurs at the frequency

2 $$\omega_{0}=\frac{1}{\sqrt{L_{0}C_{0}}}$$

At resonance, a parallel LC circuit creates a periodic signal because the energy constantly oscillates between the capacitor’s electric field and the inductor’s magnetic field. This back-and-forth energy transfer happens at a constant frequency, resulting in a natural, smooth, and continuous oscillation–just like a sine wave. The inductor and capacitor draw equal and opposite currents because their reactances cancel each other out. The inductor generates a current that lags the voltage, while the capacitor generates a current that leads the voltage by the same amount. These two currents are perfectly out of phase and equal in magnitude, so they cancel each other. As a result, no net current flows into the circuit from the external source. From the perspective of the source, it is as if the circuit is not drawing any current at all–essentially behaving like an open circuit. This is why the impedance of the parallel LC circuit appears infinite at the resonant frequency.

The resonance is a useful picture, arising from the combination of inductor and capacitor, that explains some aspects of neuron properties.^54,172,173^ However, our target is to develop oscillator systems that provide a periodic response under a constant input. In the absence of an external periodic forcing, losses in the inductor, represented by a resistance *R*_*L*_ in Figure 10a, cause the energy to fade out, and the peak of the impedance is limited as shown in Figure 10b. The solution to hold stationary undamped oscillations is to add a device with a NDR (green block in Figure 10a) in parallel or series. A circuit providing a NDR can counteract the positive resistance (losses) in the tank circuit, thereby canceling out the damping, sustaining oscillations in the case of oscillators, or even causing amplification.

NDR does not mean “less than zero ohms” in a typical resistor sense. It means that when voltage increases, current decreases, i.e., it is a differential resistance where the device actually supplies power instead of dissipating it. NDR is a common feature in electronic oscillatory devices such as Esaki tunnel diodes, Gunn diodes and resonant tunneling diodes.^174,175^ The green block in Figure 10a is a nonlinear element, and the typical shapes of NDR elements are shown in Figure 10(c, d). The N-type is bistable in current, and the S-type is bistable in voltage. More complicated situations may emerge,^26^ but in the simple *I* - *u* diagrams we can appreciate an important point. The NDR element needs to be held at the red points, where the differential resistance is unstable. Meanwhile the internal voltage and current are not fixed since they will be oscillating around the fixed point. A main question that appears in the search for self-sustained oscillations is what will be the internal voltage when applying a constant external input of voltage or current. To answer this question, one needs to carry out a stability analysis and find the condition of bifurcation. This will be fully explained in Sec. [Sec sec4.5], to establish when the circuit will start to oscillate under control of the external voltage, current, or other material parameters.

Several electronic components and circuit configurations can exhibit NDR characteristics, playing a crucial role in sustaining oscillations across a variety of applications.^175^ Tunnel diodes are among the earliest devices known for this behavior. They utilize quantum tunnelling to produce a distinctive region where the current decreases as the voltage increases in their current-voltage curve. This NDR region enables their use in high-frequency oscillators and amplifiers. Gunn diodes, commonly employed in microwave oscillators, exploit bulk NDR arising from domain formation in certain semiconductors such as gallium arsenide (GaAs). Similarly, IMPATT diodes generate high-power microwave oscillations by combining avalanche breakdown with transit-time effects, both of them contributing to their NDR behavior. Although thyristors and silicon-controlled rectifiers are not inherently NDR devices, they can be arranged in relaxation oscillator circuits to mimic NDR under specific switching conditions.

Another class of NDR devices constitutes a main topic of this paper. Such devices are formed by materials that contain a mechanism of intrinsic instability, and operate at much lower frequencies. One well-known representative of this class is that of electrochemical oscillators,^27,45–47^ further explained in Sec. [Sec sec5.2]. Another important example, already mentioned in the introduction, is that of binary oxide memristors such as VO_2_ that yield an S-type resistance associated with a MIT that have been amply researched for OBCs.^1,36,37,59–66^

With regard to the other standard approach, i.e. the feedback-based oscillator circuits (see Sec. [Sec sec7]), we have focused initially on classical configurations such as Colpitts or Hartley oscillators, which do not rely on the intrinsic switching behavior of transistors. Instead, these circuits employ the tank resonator in combination with feedback loops and active gain components to compensate for energy losses and sustain stable oscillations. In these designs, the circuit also presents an effective NDR to the LC tank, which compensates for energy losses and maintains steady oscillation.^43^

Although these two methods will be treated separately some mixed situations and connections may exist. In fact, NDR acts like a hidden amplifier: It feeds energy back into the system, causing positive feedback to happen naturally. The introduction of hybrid oscillator architectures that incorporate physical resonant elements, such as devices with intrinsic NDR, alongside electronic feedback and amplification, will illustrate how both mechanisms can be jointly engineered to produce highly controlled self-sustained oscillations.

### 3.2 Limit Cycle Oscillators

Dynamical systems are characterized by a set of differential equations, variables and parameters. It is important to distinguish the difference between the last two ones. While variables are usually associated with a physical or chemical magnitude that changes in time, like an internal intensity or voltage, thus describing the dynamical behavior of the system, parameters are associated with magnitudes that remain unchanged during the system evolution, such as an input constant current or a capacitance. It is interesting to remark that in some situations a small change in the value of a parameter can produce a big change in the system’s behavior, say for example from a stable to an unstable or an oscillatory regime. In fact, this phenomenon constitutes one of our main topics here.

In this context, it arises the concept of phase space, which is a multidimensional space where each dimension corresponds to a system variable.^17,22^ Therefore, each point ***X*** in this space corresponds to a specific state of the system at a given time, and the solutions of the differential equations that define the system, for some given initial conditions, are trajectories in this phase space. In other words, such trajectories describe the evolution of the system with time, both forward and backward and they depend, of course, on the system’s parameters. Because self-sustained oscillations correspond to stable periodic changes of variables, they draw circular-like trajectories in the phase space that are termed limit cycles.

Figure 11a shows the structure of an oscillatory system that is a major focus of this work. It is composed of a nonlinear element (green square) characterized by an internal set of variables {*x*_*i*_}, and external elements similar to the tank circuit approach of Figure 10a. Preliminary ingredients of these models are reviewed in the Appendices: The models for memristors and chemical inductors without a NDR are revised in Appendix 1, and the simplest, one-equation dynamical model, indicating the transient behavior of a NDR, is discussed in Appendix 2.

**Figure 11:**
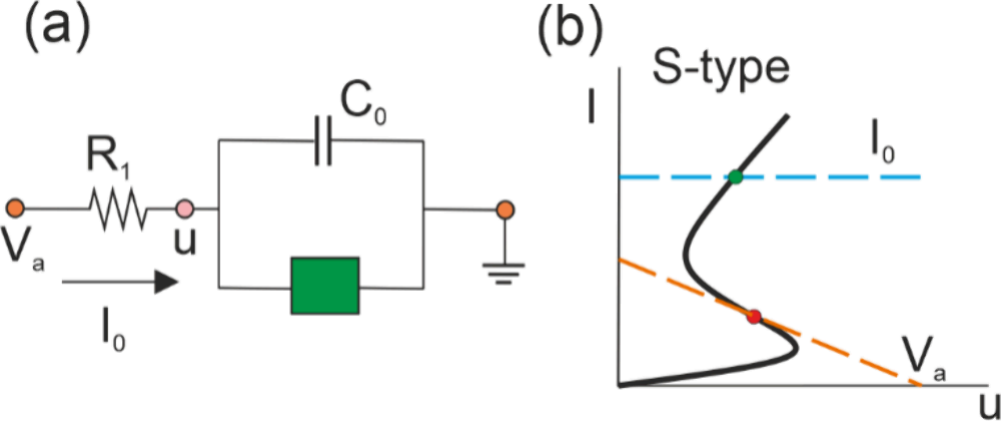
(a) Oscillatory circuit. (b) Current–voltage characteristic curve, indicating the load lines at constant current (blue) and constant voltage (orange).

In the limit cycle oscillator models associated with Figure 11a, one general equation is due to the coupling of the system’s overall resistance with an external capacitor, and this is usually described by an internal voltage *u*. An additional equation occurs for each of the internal state variable set {*x*_*i*_} that characterize the nonlinear element. These *x*_*i*_ variables are necessary to determine the future behavior of the system when the present state and the inputs are known, and they are system-dependent since they represent different internal properties of the nonlinear (active) element, as in memristor theory.^176^ Thus, we can have a minimum of two differential equations for a single *x*, when the phase space is ***X*** = (*u*, *x*), three differential equations for X = (*u*, *x*_1_, *x*_2_), and so on.

The set of variables {*x*_*i*_} can be an idealization of essential variables in a nonlinear system that may have multiple degrees of freedom, spatial inhomogeneities, etc. We will be content if the minimal chosen set is able to describe the main features of the oscillatory properties of the system. Further understanding can be obtained by realistic physical simulation of the system, but this has the disadvantages of intensive computation with a large number of parameters so that the bifurcation relations, that are explained below, are obscured with respect to the standard approach based on a few differential equations.

Our modeling approach is flexible enough to describe many different types of physical systems. Because of this, the behavior of the internal variables (represented as {*ẋ*_*i*_}) can vary widely depending on the specific system under consideration. To understand how these systems behave, we focus on analyzing how their dynamics change–especially when small changes in conditions cause a big shift in behavior, a concept known as bifurcation. One way to study this is by looking at how the system behaves near stable points, using linear local analysis of the differential equations. This kind of analysis is powerful and often tells us a lot about the system’s general behavior.^23–25^

However, when we deal with limit cycle oscillators (systems that naturally repeat their behavior over time), the equations describing the internal variables {*x*_*i*_} often take a special common form called relaxation equations, in which *ẋ*_*i*_ = *G*_*i*_ (*u*, *x*_1_, *x*_2_, . . .) where G is a nonlinear function. These equations, explained in Sec. [Sec sec4.2], describe how the system quickly returns to equilibrium after being disturbed and can create an inductor-like effect,^177^ a kind of memory or delay in the system’s response. One remarkable universal fact is that all limit-cycle oscillators behave in a similar manner close to the onset of the oscillations, despite great disparity of the oscillations from system to system.

Interestingly, this behavior is closely related to how memristors work (Appendix 1), and in Sec. [Sec sec4.3], we will discuss the connection between memristors, inductors and self-sustained oscillators in more detail.

We are going to explore the effects of various bifurcations that arise as system parameters change, highlighting the emerging complex oscillatory behaviors. This type of study is a central guidance to build and understand self-sustained oscillations, following the central example of the HH model in neuroscience. The higher the number of state variables, the more complex oscillatory phenomena can be obtained. Moreover, each of the equations *ẋ*_*i*_ = *G*_*i*_ introduces a time scale parameter *τ*_*i*_, and the relative value of these important time constants {*τ*_*i*_} is a central aspect of bifurcations, leading to distinct oscillatory patterns, as discussed in Sec. [Sec sec5].^178^

This approach is very well developed in electrochemical oscillators, for example, where dedicated experimentation has established detailed models using key experimental variables as internal voltage, concentrations, etc.^27,45–47^ The method finds great parallelism in many branches of science, e.g., chemical reactions,^23^ optics, biology, and biochemistry,^125,126^ for which ordinary differential equations models form a natural basis for mathematical analysis. The limit cycle oscillators are a basic component of wider classes of distributed problems where a global solution enlightens other computational problems,^39,40,101^ and may help clarifying self-organization phenomena and spatiotemporal complexity.^51^

## 4 Two-Equation Neuron Oscillators Based on an NDR

This section introduces mathematical models to formalize the dynamical mechanisms underlying self-sustained oscillations. While the equations provide a rigorous description of stability and bifurcation phenomena, the main physical insights can be followed through the accompanying qualitative discussion and figures. We have remarked that the occurrence of oscillations necessitates a NDR behavior. This condition ensures that small perturbations in the system can lead to sustained oscillations rather than simply returning to equilibrium. The discussion in Appendix 2 shows that one variable is normally not enough to make an oscillatory system, since the reactance of the capacitor is not counteracted. Therefore, the minimal limit cycle oscillator is composed of two first order differential equations, as we discuss in the next section. In Sec. [Sec sec4.2] we show this structure with the FitzHugh-Nagumo (FHN) neuron model.

### 4.1 Dynamical Systems Consisting of Two Equations

We start a general analysis of the system of Figure 11a, commented in Sec. [Sec sec3.2], where the green element has a current function *I*_*g*_(*u*, *x*) for a single state variable *x*. The dynamical system is described by the equations

3 $$I_{0}=\frac{V_{a}-u}{R_{1}}$$

4 $$\frac{du}{dt}=\frac{1}{C_{0}}\left[I_{0}-I_{g}\left(u,x\right)\right]$$

5 $$\frac{dx}{dt}=G\left(u,x\right)$$

More generally, let us consider a system of coupled differential nonlinear equations,

6 $$\frac{du}{dt}=F\left(u,x,I_{0}\right)$$

7 $$\frac{dx}{dt}=G\left(u,x\right)$$

Here *u* is the voltage in the device and *x* is an internal state variable. The phase space point is a vector ***X*** = (*u*, *x*). *F* and *G* are multivalued nonlinear functions that form the velocity vector in phase space,

8 $$V=\frac{dX}{dt}=\left(\frac{du}{dt},\frac{dx}{dt}\right)=\left(F,G\right)$$

Dynamical methods provide the asymptotic behavior of the trajectories in phase space by analyzing the steady state of the problem and its long-term performance.^24,25^ By solving the algebraic system

9 $$F\left(u,x,I_{0}\right)=0$$

10 G(u,x)=0

we obtain the steady-state solution of (6, 7) at a point ***Y***_0_ = (*u̅*, *x̅*, *I̅*_0_) that denotes both the phase space equilibrium point ***X***_0_ = (*u̅*, *x̅*) and the forcing parameter *I̅*_0_. Both curves describing eqs ([Disp-formula eq9], 10) are called nullclines. Nullclines represent the set of points in the system where a particular variable does not change over time, meaning its rate of change is zero. These points correspond to zero phase space velocity component of that variable.^179^ The steady state point ***Y***_0_ is at the intersection of the nullclines, where the equilibrium condition ***V*** = 0 is satisfied. Here, it should be clarified the fact that an equilibrium or steady point does not mean the system is stable there. If small disturbances away from it return the system to the same point, then the equilibrium is stable. Otherwise, if any small disturbance grows in time, then the equilibrium is unstable.

Many oscillator models are described by a system of two equations: one representing a fast, destabilizing variable and the other a slow, stabilizing variable, which together form what is commonly referred to as a slow-fast dynamical system.^180,181^ Such systems are characterized by the presence of distinct time scales, where the fast variable undergoes rapid changes while the slow variable evolves more gradually, providing a stabilizing influence. The interplay between the fast and slow variables, coupled with the NDR characteristic, enables the emergence of self-sustained oscillations. In the FHN model, explained in Sec. [Sec sec4.2], the fast-destabilizing value is *u*, and the inductor is the stabilizing element. In S-type oscillators, explained in Sec. [Sec sec4.7], we find the opposite situation.^42^ For systems composed of more than two dynamical equations, the time constants associated with the respective equations {*τ*_*i*_} can form different combinations of fast and slow variables, that lead to complex oscillatory phenomena.^118,178,182^ This will be analyzed in Sec. [Sec sec5].

In some areas, especially those inspired by biological or chemical systems, the two-variable oscillating dynamical system (like the FHN model) is often formulated in terms of an *activator* and an *inhibitor* that describe two interacting components. The activator is a variable or process that promotes its own increase through positive feedback, driving the system away from equilibrium and toward a more active state. In contrast, the inhibitor serves to counteract the activator’s influence, introducing negative feedback that suppresses or limits the system’s activity. The interplay between these two forces, namely, excitation by the activator and suppression by the inhibitor, creates a cyclical pattern in which the system repeatedly rises and falls in activity. This balance of delayed feedback loops ensures that the system does not stabilize at a constant value, but instead oscillates in a sustained and self-regulated manner over time. A paradigmatic example can be found in electrochemical oscillatory systems^27,45–47^ where the positive feedback variable is typically either the electrode potential (*E*) or the surface coverage of adsorbed species (θ), as further explained in Sec. [Sec sec5.2]. In the FHN model, the fast activator is *u* (representing membrane voltage) and *x* (representing recovery processes like ion channel kinetics) is the slow inhibitor.

Mathematical models constructed using two fundamental interacting variables that lead to self-sustained oscillatory behavior, such as the activator-inhibitor system, have found widespread applications across various scientific and engineering disciplines.^49,183,184^ These include neuroscience, where they describe rhythmic neuronal firing; biological systems, such as population dynamics and enzyme kinetics; electrical circuits exhibiting oscillatory behavior; and climate science, where they help model cyclical variations. The versatility of such models underscores their significance in understanding and predicting complex dynamical phenomena in both natural and artificial systems. For example, many VO_2_ oscillator devices can usually be described by only two differential equations.^185,186^

However, other systems require a larger number of memory variables, as will be reviewed in Sec. [Sec sec5]. For instance, some electrochemical oscillators are often composed of three memory variables giving four differential equations,^45,187^ the same number as those of the HH model. A combined list of the models discussed in the paper is shown in Table 1.

**Table 1:** Oscillatory Models

	Model	Equations	Application
	**2-variables**		
1	FitzHugh-Nagumo neuron	$$\begin{array}{l} \frac{du}{dt}=\frac{1}{C_{0}}\left(I_{0}-x\right)+\frac{1}{\tau_{0}}\left(u-\frac{u^{3}}{3}\right)\\ \frac{dx}{dt}=\frac{1}{\tau_{L}R_{a}}u-\frac{x}{\tau_{L}} \end{array}$$	Describes a N type-NDR oscillatory system. Figure 12.
2	Izhikevich	$$\begin{array}{l} \frac{du}{dt}=0.04^{2}+5u+140-x+I\\ \frac{dx}{dt}=a\left(bu-x\right) \end{array}$$	An IF model that reproduces most of the spiking regimes of the Hodkin- Huxley model. Figure 19.
		If *u* > 30 then u = c and x = x+d	
3	Rinzel^188^ and Wilson^14^ neuron	$$\begin{array}{l} \frac{du}{dt}=\frac{1}{C_{0}}\left[I_{0}-g_{Na}\left(u\right)\left(u-E_{Na}\right)-x\left(u-E_{K}\right)\right]\\ \frac{dx}{dt}=\frac{1}{\tau_{k}}\left[R\left(u\right)-x\right] \end{array}$$	Reproduces the essential dynamics of the HH model but in a simplified 2D version.
4	Morris-Lecar neuron^189^	$$\begin{array}{l} \frac{du}{dt}=\frac{1}{C_{0}}\left[I_{0}-g_{Na}m_{eq}\left(u\right)\left(u-E_{Na}\right)-g_{K}x\left(u-E_{K}\right)-g_{Leak}\left(u-E_{Leak}\right)\right]\\ \frac{dx}{dt}=\frac{1}{\tau_{k}\left(u\right)}\left[x_{eq}\left(u\right)-x\right] \end{array}$$	Describes the voltage oscillations in the barnacle giant muscle fiber. It can be considered as a simplified version of the HH model. Figure 23.
		*x*_*eq*_ and *τ*_*k*_ have shapes similar to *m*_*eq*_ and *τ*_*m*_ respectively in model 7	
5	S-type cubic	$$\begin{array}{l} \frac{du}{dt}=\frac{1}{C_{0}}\left(I_{0}-\frac{u}{a\left(x^{2}-3bx+3b^{2}\right)-c}\right)\\ \frac{dx}{dt}=\frac{1}{\tau_{k}}\left(\frac{u}{a\left(x^{2}-3bx+3b^{2}\right)-c}-x\right) \end{array}$$	Describes a S type-NDR oscillatory system. Figure 15.
	**3-variables**		
6	Koper^118^	$$\begin{array}{l} \frac{du}{dt}=\frac{v-u}{r}-mk\left(u\right)x\\ \frac{dx}{dt}=-k\left(u\right)x+\left(w-x\right)\\ \frac{dw}{dt}=\frac{19}{333}\left(16-25w+9x\right)\\ k\left(u\right)=k_{1}\theta^{2}+k_{2}\exp\left[\alpha n\left(u-u^{0}\right)\right]\\ \theta =\{\begin{array}{ll} 1 & u\leq u_{d}\\ \exp\left[-b{\left(u-u_{d}\right)}^{2}\right] & u>u_{d} \end{array} \end{array}$$	Describes the electrochemical polarographic reduction of In^3+^ to In^0^ in thiocyanate containing media, producing MMO. Figure 20.
	**4-variables**		
7	Hodgkin-Huxley	$$\begin{array}{l} I_{ext}=C_{m}\frac{du}{dt}+g_{Na}m^{3}h\left(u-E_{Na}\right)+g_{K}n^{4}\left(u-E_{K}\right)+g_{leak}\left(u-E_{leak}\right)\\ \tau_{m}\left(u\right)\frac{dm}{dt}=m_{eq}\left(u\right)-m\\ \tau_{h}\left(u\right)\frac{dh}{dt}=h_{eq}\left(u\right)-h\\ \tau_{n}\left(u\right)\frac{dn}{dt}=n_{eq}\left(u\right)-n\\ m_{eq}\left(u\right)=\frac{1}{1+e^{\left\{\left(V_{1/2}-u\right)/k\right\}}} \end{array}$$	Initially used to explain the behavior of the squid giant axon. It reproduces the dynamics of a neuron voltage membrane modulated by gated ion channels. Figure 21.
		*h*_*eq*_ and *n*_*eq*_ have a shape similar to *m*_*eq*_	
		$$\tau_{m}\left(u\right)=C_{base}+C_{amp}\exp\left(\frac{-{\left(V_{\max}-u\right)}^{2}}{\sigma^{2}}\right)$$	
		*τ*_*h*_ and *τ*_*n*_ have a shape similar to *τ*_*m*_	

### 4.2 FitzHugh-Nagumo Model

The FHN model is one of the simplest models of a limit cycle oscillator with nontrivial behavior.^190–194^ FHN model is a development of the seminal van der Pol oscillator and it is used in a multitude of neural oscillators.^30,52,195–199^ Both models are representatives of the wider class of N-type oscillators.

The model as first developed by FitzHugh^190^ reduces the complex dynamics of HH model of neuronal behavior into a two-variable system. As we will see, this is an elementary form of an oscillatory system with a Hopf bifurcation. Nagumo et al.^191^ showed that the model corresponds to the electrical circuit of Figure 12a. They used a tunnel diode for the nonlinear element with NDR. It can be observed that the circuit of Figure 12a is a literal representation of the general model of Figure 10a.

**Figure 12:**
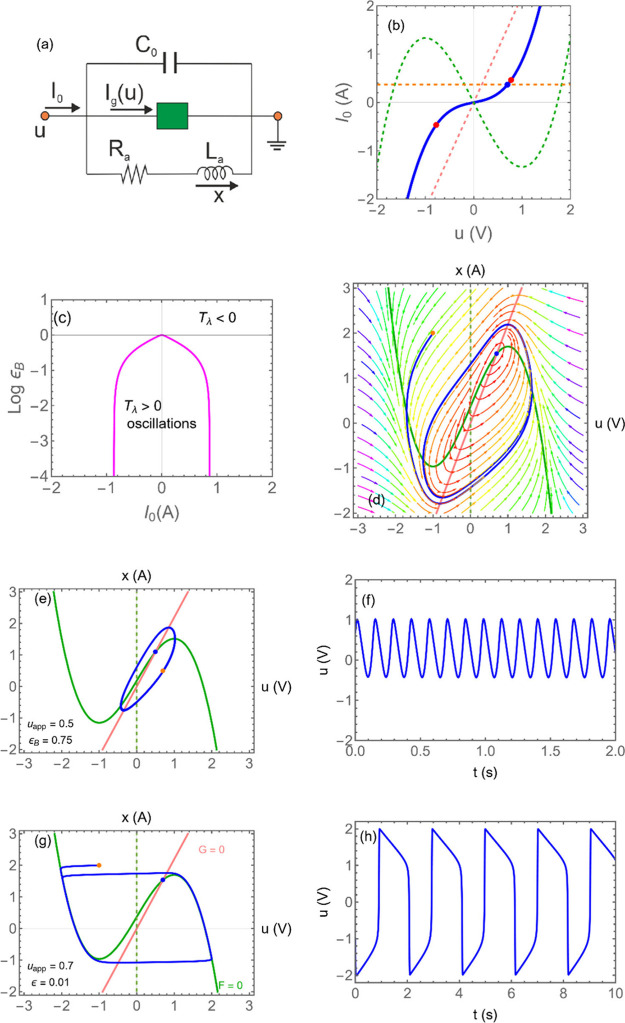
FHN model. (a) Electrical representation. *u* voltage; *I*_0_ input current; *x* = *i*_*L*_ current in the inductor; *I*_*g*_(*u*) variable resistor; *C*_0_ capacitance; *R*_*a*_ resistor; *L*_*a*_ inductor. (b) *u* - *I*_0_ plane. The green line is the fast component of the current, and the pink line is the slow one. When added they give the blue line that is the stationary *u* - *I*_0_ characteristics. The blue point is the steady-state point at the nominal potential (*u*_*app*_ = 0.7) and the orange line is the corresponding constant current *I*_0_ = 0.387 *A*. The red points are the Hopf bifurcations at *u*_*B*_ = 0.775 *V*. (c) Bifurcation diagram at fixed *r*_0*a*_ = *R*_0_/*R*_*a*_. (d) The green line is the nullcline *F* = 0, and the pink line is the nullcline *G* = 0. Trajectories and vector velocities at constant current *I*_0_ = 0.507 *A* are shown. The orange point is the starting condition. Color streamlines indicate the norm of the vector field. Parameters: *R*_0_ = 0.5 Ω ;*R*_*a*_ = 0.455 Ω ; τ_0_ = 0.01 *s*; ϵ = 0.4, where ϵ = τ_0/_ *τ*_*L*_ (e, g) Evolution and nullclines in *u* - *x* plane with ϵ and *u*_*app*_ as indicated. The blue point is the fixed point at the nominal potential. The orange point is the initial condition. (f, h) Voltage evolution with time.

The two variables are the membrane voltage *u*, and the internal recovery current *x* (the current in the inductor) that represents the changes in ion-channel conductance as a function of the voltage. The total transmembrane current *I*_0_ is a parameter. The circuit shown in Figure 12a is described by the equations^197,200^

11 $$C_{0}\frac{du}{dt}=I_{0}-I_{g}\left(u\right)-x$$

12 $$L_{a}\frac{dx}{dt}=u-R_{a}x$$

The FHN model is defined by the variable resistor of the form

13 $$I_{g}\left(u\right)=\frac{1}{R_{0}}\left(\frac{u^{3}}{3}-u\right)$$

where *R*_0_ is a constant resistor parameter. The stationary solution of (11, 12) is

14 $$I_{0}=\frac{1}{R_{0}}\left(\frac{u^{3}}{3}-u\right)+\frac{u}{R_{a}}$$

This is the blue line in Figure 12b. The total current has two components, the green curve is Eq. 13 and the pink curve is *I* = *u*/*R*_*a*_.

The FHN equations are given in Table 1.1 and we have the forms

15 $$F=\frac{1}{\tau_{0}}\left(u-\frac{u^{3}}{3}\right)+\frac{1}{C_{0}}\left(I_{0}-x\right)$$

16 $$G=\frac{1}{\tau_{L}R_{a}}u-\frac{x}{\tau_{L}}$$

Nullclines are indicated in Figure 12(e, g). They intersect at the blue point, and a close loop trajectory arises around it for any initial condition, which produces voltage oscillations as shown in Figure 12(f, h). This is the stable oscillatory solution called a limit cycle.

There are some requirements for a dynamical system to produce this oscillatory behavior, as we explain in the next sections. The system is characterized by the time constants for the voltage response time *R*_0_ *C*_0_

17 $$\tau_{0}=R_{0}C_{0}$$

and the recovery current response time of the inductive *R*_*a*_ *L*_*a*_ subcircuit

18 $$\tau_{L}=\frac{L_{a}}{R_{a}}$$

Further dynamical behaviors shown in Figure 12 are discussed in the next sections.

### 4.3 When the Inductor Comes for Free: No Coil Needed

We have emphasized before the NDR as a necessary nonlinear element for obtaining self-sustained oscillations. This central feature will be commented in Sec. [Sec sec4.5]. But before that, we would like to remark a significant difference between the engineering circuit of Figure 10a and our reference circuit of Figure 11a. The latter does not contain an inductor, which also is a necessary ingredient of oscillations! In the following, we are going to show that Eqs. ([Disp-formula eq4], 5) already provide an inductor, without the need to bring one in as it happens in the FHN model.

To derive this property, a small ac input signal, Δ*I*_0_, is introduced in the system, which is assumed to react in a linear way because of the small perturbation. Then, the resulting impedance is calculated at a stationary point ***Y***_0_ = (*u̅*, *x̅*, *I̅*_0_) on the characteristic curve. This linearization method is explained with simple examples in Appendix 2. The linearized equations for the phase space vector small variables Δ ***X*** = (Δ*u*, Δ*x*) are

19 $$\frac{d}{dt}\left[\begin{array}{l} \Delta u\\ \Delta x \end{array}\right]=J_{X}\left[\begin{array}{l} \Delta u\\ \Delta x \end{array}\right]+\frac{1}{C_{0}}\left[\begin{array}{l} \Delta I_{0}\\ 0 \end{array}\right]$$

The Jacobian matrix evaluated at ***Y***_0_ is

20 $$J_{X}=\left[\begin{array}{ll} F_{u}\left(Y_{0}\right) & F_{x}\left(Y_{0}\right)\\ G_{u}\left(Y_{0}\right) & G_{x}\left(Y_{0}\right) \end{array}\right]$$

Here the subscript denotes partial derivatives, defined by *F*_*u*_ = ∂*F*/∂*u*, from Eqs. ([Disp-formula eq4], 5), and removing the explicit indication to calculate at ***Y***_0_, we have

21 $$J_{X}=\left[\begin{array}{ll} -\frac{1}{R_{b}C_{0}} & -\frac{I_{gx}}{C_{0}}\\ G_{u} & G_{x} \end{array}\right]$$

where

22 $$R_{b}={\left(\frac{\partial I_{g}}{\partial u}\right)}^{-1}$$

and

23 $$I_{gx}=\frac{\partial I_{g}}{\partial x}$$

We make the change *d*/*dt* → *s* = *iω* in Eq. 19, where *s* is the Laplace variable, and obtain the equations

24 $$\Delta I_{0}=C_{0}s\Delta u+\frac{1}{R_{b}}\Delta u+I_{gx}\Delta x$$

25 $$s\Delta x=G_{u}\Delta u+G_{x}\Delta x$$

Solving the system for Δ*x*, we get the impedance

26 $$Z\left(s\right)=\frac{\Delta u}{\Delta I_{0}}={\left(\frac{1}{R_{b}}+C_{0}s+\frac{I_{gx}G_{u}}{s-G_{x}}\right)}^{-1}$$

The third term inside the parentheses can be transformed to the form

27 $$Z\left(s\right)={\left(\frac{1}{R_{b}}+C_{0}s+\frac{1}{R_{a}+L_{a}s}\right)}^{-1}$$

where

28 $$R_{a}=-\frac{G_{x}}{I_{gx}G_{u}}$$

is a resistor and

29 $$L_{a}=\frac{1}{I_{gx}G_{u}}$$

is an inductor. The complete equivalent circuit, taking into account the addition of *R*_1_, is shown in Figure 13a. The DC resistance of the circuit is

30 $$R_{d}=R_{1}+{\left(\frac{1}{R_{a}}+\frac{1}{R_{b}}\right)}^{-1}$$

**Figure 13:**
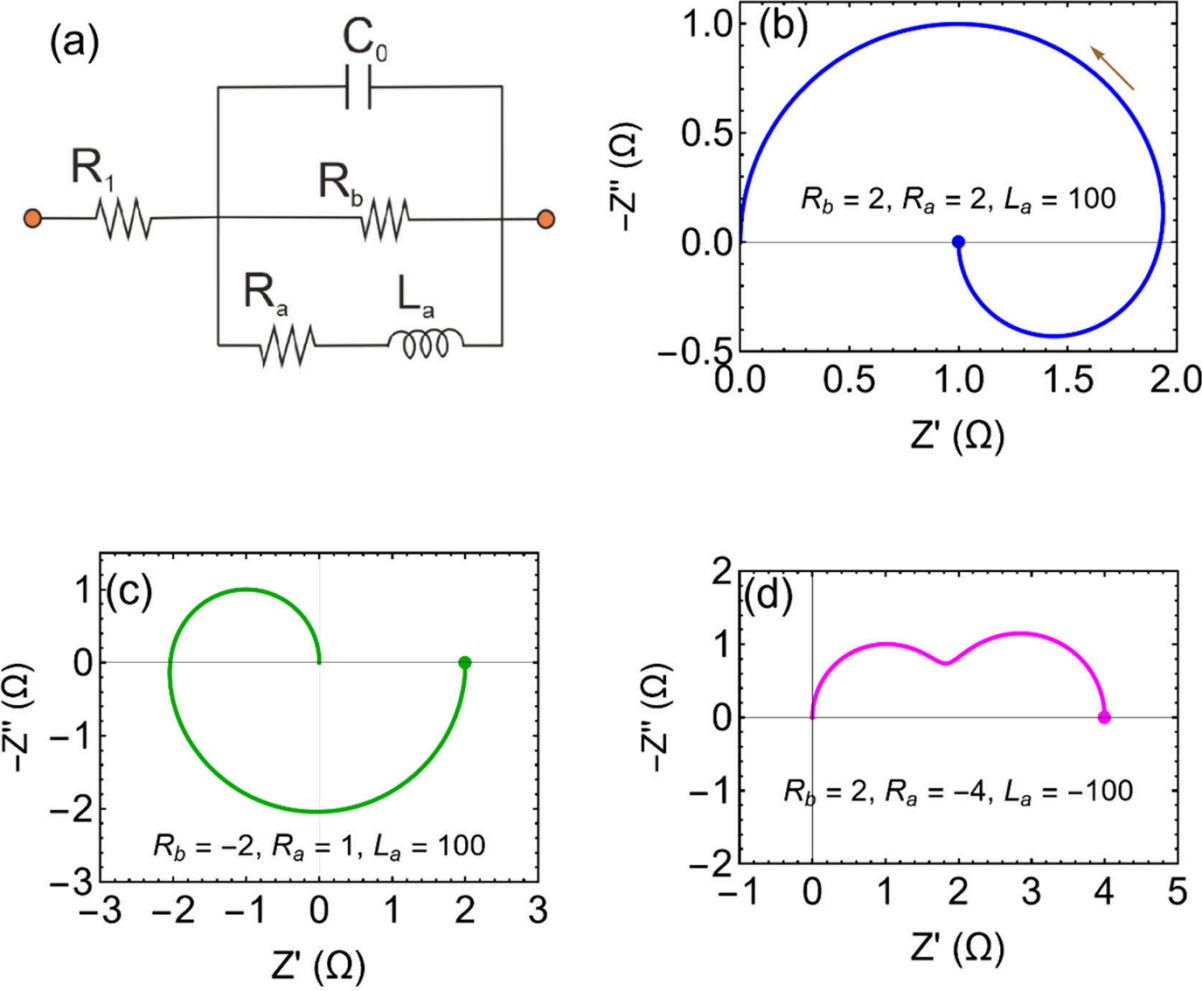
(a) Equivalent circuit of the chemical inductor. (b–d) Impedance spectra for *R*_1_ = 0, *C*_0_ = 1 *F*, and other parameters as indicated (*R* in Ω, *L*_*a*_ in H). The point is the DC value *Z*(ω = 0), and the arrow indicates the direction of increasing frequency.

When all the circuit elements are positive, the spectrum of Figure 13b is obtained. We observe a capacitive characteristic at high frequency, due to the capacitor *C*_0_ in the model, and an inductive shape at low frequency (also termed a negative capacitance^201–206^ ). This is a very general characteristic of all systems formed by two equations as Eqs. ([Disp-formula eq4], 5), whatever the physical interpretation of the state variable *x* might be. Consequently, this system has been denominated generally a chemical inductor,^177^ and the impedance characteristics have been amply discussed.^30,198^ The Eqs. ([Disp-formula eq4], 5) can also be considered in a general sense as describing a memristor, as indicated in Eqs. ([Disp-formula eqA1.1], [Disp-formula eqA1.2]).^176,177,207,208^ The presence of an inductor in a memristor has already been featured,^209^ which has important implications for synaptic devices and is discussed in Appendix 1.

The spectra corresponding to negative values for resistors and inductors of the system are shown in Figure 13(c, d). They will be considered later on.^198^

To obtain further insight into the abstract expressions of Eqs. ([Disp-formula eq4], 5) we consider the example of Eq. [Disp-formula eqA1.2] where the *x*-variable describes an activation function whose value varies between *x* = 0 and *x* = 1. The adaptation equation has the form

31 $$\tau_{k}\left(u\right)\frac{dx}{dt}=x_{eq}\left(u\right)-x$$

so that in equilibrium *x* → *x*_*eq*_.

Let us define the chemical capacitance^210^

32 $$c_{\mu }=\frac{dx_{eq}}{du}$$

By evaluation of the partial derivatives, we obtain

33 $$G_{u}=\frac{c_{\mu }}{\tau_{k}}$$

34 $$G_{x}=-\frac{1}{\tau_{k}}$$

35 $$R_{a}=\frac{1}{I_{gx}c_{\mu }}$$

36 $$L_{a}=\frac{\tau_{k}}{I_{gx}c_{\mu }}$$

We find that the inductor response time corresponds to the relaxation time in Eq. 31

37 $$\tau_{L}=\frac{R_{a}}{L_{a}}=\tau_{k}\left(u\right)$$

Therefore, we conclude that it is not necessary to add an inductor to the model in Figure 11a, since this component is already accounted for by the nonlinear element, as shown in the small-signal impedance analysis. It must be noted, however, that the inductance and the resistance associated with this inductor are not constant. They can vary along the stationary curve, and even become negative. Nevertheless, in an important class of situations like that described by Eq. 31 the inductor response time is specified directly by the relaxation time.

In summary, for making compact self-sustained oscillators it is highly desirable to have a nonlinear element that generates both a NDR and a chemical inductor, by some physicochemical mechanism that produces an internal degree of freedom represented by a variable *x*.

### 4.4 Voltage and Current Operation

As we have explained, a self-sustained oscillator operates with a constant input that supplies energy. As the main representative situations of the stationary energy input we distinguished in Figure 11b two fundamental modes of operation: one with a constant applied current, *I*_0_, and the other one with a constant applied voltage *V*_*a*_. There are significant differences regarding the oscillator response,^180,211^ and each of them offers distinct advantages and disadvantages depending on the intended application and system-level requirements.

The load line refers to a graphical tool used for analyzing and designing oscillator devices. It helps determine the operating point and understand how the active device interacts with the rest of the circuit, especially in what it concerns feedback and tuning network. The load line is a straight line drawn on the device’s characteristic curve that represents the constraints imposed by the external circuit (especially the resistive load and supply voltage). Figure 11b shows the load lines of an S-type oscillator operated at constant current (blue line) and at constant voltage (orange line). In the case of constant current, the load line is simply a horizontal line that determines a stable point (green).

In the case of constant voltage, Eq. 4 is substituted by

38 $$\frac{du}{dt}=\frac{1}{C_{0}}\left[\frac{V_{a}-u}{R_{1}}-I_{g}\left(u,x\right)\right]$$

The load line is the straight line obtained from Eq. 3 and it sets the operation at a NDR point (red).

When the oscillator operates in constant current mode, also known as galvanostatic mode in electrochemical terms, the external resistor shown in Figure 11a becomes irrelevant, since the current is fixed externally and does not depend on the series resistance. In contrast, during constant voltage operation, referred to as potentiostatic mode, the resistor plays a crucial role in determining the current that flows through the system. As a result, it becomes an important design parameter that directly affects the oscillator’s behavior. While this added flexibility can be useful for fine-tuning the circuit’s dynamics, it also introduces additional complexity in both the design and analysis stages.

Operating with a constant voltage source is generally more compatible with modern neuromorphic circuit design, particularly due to its alignment with traditional CMOS technology. CMOS-based systems typically process voltage signals, making voltage-mode circuits easier to integrate with existing digital and mixed-signal components. Moreover, voltage-mode operation enables the design of circuits that can function at very low supply voltages, thereby reducing overall power consumption–a critical factor in energy-efficient and battery-powered devices. Additionally, the widespread availability of voltage amplifiers, buffers, and other support components further simplifies the implementation of voltage-based oscillator systems.

On the other hand, current-mode operation presents unique advantages, especially when emulating the behavior of biological neural systems. In biology, information processing and communication between neurons are primarily driven by ionic currents crossing cell membranes. As a result, circuits designed to operate in current mode can more naturally and directly replicate these biological mechanisms. From a hardware perspective, current-mode circuits can offer higher density and simplicity, as electrical currents can be summed directly at junctions or nodes without the need for bulky passive components like resistors or capacitors. This property not only leads to more compact circuit layouts but also facilitates the design of parallel, large-scale neuromorphic networks.

Ultimately, the choice between constant current or voltage operation depends on the specific goals of the oscillator circuit–whether prioritizing compatibility with conventional electronics, minimizing power, maximizing biological realism, or achieving compactness and scalability.

### 4.5 Hopf Bifurcation, Stability, and Oscillations

In some cases, particular values of a system parameter can lead to qualitative changes in the system’s stability. These critical values are called bifurcation points, and the associated parameter is known as the bifurcation parameter. A Hopf bifurcation is a fundamental concept in dynamical systems theory that describes a transition from a stable equilibrium to a periodic oscillation regime when a system parameter crosses a critical threshold. For a Hopf bifurcation to occur, at least two interacting variables are required, as discussed in Sec. [Sec sec4.2], so that Eqs. ([Disp-formula eq6], 7) form a minimal oscillatory model. The Hopf bifurcation mechanism, which governs the transition between stationary and oscillatory states, plays a pivotal role in shaping the behavior of artificial neurons.

Properties of the bifurcation and stability of a dynamical model can be effectively characterized by linear local analysis of the differential equations, a method we have used in Eq. 19. Hence, classification of the different trajectories of evolution in a multidimensional phase space uses a linearization process to obtain the small perturbation velocity vector field Δ ***V***

39 $$\Delta V=\frac{d\Delta X}{dt}=\left(\frac{d\Delta u}{dt},\frac{d\Delta x}{dt}\right)$$

For a fixed external parameter (Δ*I*_0_ = 0), the Eqs. 19 at a stationary point ***Y***_0_ become

40 $$\frac{d}{dt}\left[\begin{array}{l} \Delta u\\ \Delta x \end{array}\right]=J_{X}\left[\begin{array}{l} \Delta u\\ \Delta x \end{array}\right]$$

where *J*_*X*_ is the Jacobian matrix of Eq. 20. The field Δ ***V*** indicates the direction of the trajectories, that may approach the ***Y***_0_, move away from it, etc. A diagonalization procedure of *J*_*X*_ establishes the dynamical properties of the point, indicating the direction of the flow of trajectories. These general features of differential equations that represent dynamical systems are amply explained in the book of Arnold^22^ and in many other texts.^23–25^ The standard formulas for the eigenvalues are given in Appendix 3. They are solutions of the equation

41 $$\lambda^{2}-T_{\lambda }\left(Y_{0}\right)\lambda +\Delta_{\lambda }\left(Y_{0}\right)=0$$

The trace of *J*_*X*_ is

42 $$T_{\lambda }=F_{u}+G_{x}$$

and the determinant is

43 $$\Delta_{\lambda }=F_{u}G_{x}-F_{x}G_{u}$$

The general expression of a pair of conjugated eigenvalues

44 $$\lambda_{\pm }=\alpha \pm i\omega$$

provides significant dynamical information on:

(i) The time constants of the evolution in the real part α. The state ***Y***_0_ will be stable (unstable) if the real part is negative (positive).
(ii) The oscillatory solutions in the imaginary part *iω*.

To clarify the interpretation, let us define a characteristic time constant

45 $$\tau_{\lambda }\left(Y_{0}\right)=-\frac{1}{\alpha }=-\frac{2}{T_{\lambda }\left(Y_{0}\right)}$$

and a characteristic frequency

46 $$\omega_{\lambda }\left(Y_{0}\right)={\left[\Delta_{\lambda }\left(Y_{0}\right)-\frac{1}{{\tau_{\lambda }}^{2}\left(Y_{0}\right)}\right]}^{1/2}$$

Then we can write the eigenvalues (Eq 44) as

47 $$\lambda_{\pm }=-\frac{1}{\tau_{\lambda }\left(Y_{0}\right)}\pm i\omega_{\lambda }\left(Y_{0}\right)$$

In the Hopf bifurcation the stability of a steady state shifts, by a change of sign of the real part of λ when α = 0, giving rise to a periodic orbit or limit cycle, as in Figure 12(e, g).^14,23^ In other words, the real part of the eigenvalues of the Jacobian matrix *J*_*X*_ crosses zero, meaning *T*_*λ*_ = 0, which makes the steady-state point nonhyperbolic. At the same time, the determinant Δ_λ_ remains positive.^212,213^ The limit cycle is unique, with all solutions tending to it, regardless of the initial conditions.

Right at the Hopf bifurcation, when ***Y***_0_ = ***Y***_*B*_, and *T*_*λ*_ (***Y***_*B*_) = 0, sinusoidal oscillations of frequency ω_0*B*_ are obtained (Appendix 3), where

48 $$\omega_{0B}\left(Y_{B}\right)={\left[\Delta_{\lambda }\left(Y_{B}\right)\right]}^{1/2}$$

Note that Eqs. ([Disp-formula eq46], 48) are valid in a restricted vicinity of the bifurcation point. At points far from the bifurcation there may occur other types of periodic motion like relaxation oscillations. This will be explained in Secs. 4.6 and 4.7.

In a *supercritical Hopf bifurcation*, a small, stable periodic orbit (limit cycle) with small amplitude and finite frequency arises across the parameter change. This contrasts with a *subcritical Hopf bifurcation*, where unstable limit cycles appear and the equilibrium becomes unstable before the bifurcation, often leading to sudden jumps in behavior. Unless otherwise stated, our examples below refer to the supercritical Hopf bifurcation.

For the FHN model of Eqs. ([Disp-formula eq15], 16) we have

49 $$J_{X}=\left[\begin{array}{ll} \frac{1}{\tau_{0}}\left(1-u^{2}\right) & -\frac{1}{C_{0}}\\ \frac{1}{\tau_{L}R_{a}} & -\frac{1}{\tau_{L}} \end{array}\right]$$

The bifurcation parameters are

50 $$\epsilon =\frac{\tau_{0}}{\tau_{L}}=\frac{R_{0}R_{a}C_{0}}{L_{a}}$$

51 $$r_{0a}=\frac{R_{0}}{R_{a}}$$

The trace and determinant become

52 $$T_{\lambda }=\frac{1}{\tau_{0}}\left(1-u^{2}-\epsilon \right)$$

53 $$\Delta_{\lambda }=\frac{1}{\tau_{0}\tau_{L}}\left(-1+u^{2}+r_{0a}\right)$$

Hopf bifurcation points occur at the points ***Y***_*B*_ with the current *I*_*B*_ corresponding to voltages

54 $$u_{B}=\pm {\left(1-\epsilon \right)}^{1/2}$$

The ϵ parameter in Eq. 50 determines the range of voltages where oscillation occurs. At a fixed *I*_0_ the Hopf bifurcation occurs at

55 $$\epsilon_{B}\left(I_{0}\right)=1-\mathrm{u̅}{\left(I_{0}\right)}^{2}$$

with frequency

56 $$\omega_{0B}\left(Y_{B}\right)=\frac{1}{{\left(\tau_{0}\tau_{L}\right)}^{1/2}}{\left(r_{0a}-\epsilon \right)}^{1/2}$$

If the capacitor *C*_0_ is too large (or the inductor too small), which means large values of ϵ (see Eq. 50), oscillations are not permitted. This property is observed in the bifurcation diagram of Figure 12c. In addition, only for |*I*_0_| ≤ |*I*_*B*_| does *T*_*λ*_ ≥ 0 and the system shows limit cycle oscillations of frequency ω_0_ (*y*_0_) as presented in Figure 12(e, f).

We have developed the general criteria for the stability of time-dependent trajectories such as limit cycles in the language of nonlinear dynamical systems that originates from Poincaré and Lyapunov.^22^ Equivalent statements can be found in other fields. For example, control theory is a branch of engineering and mathematics that deals with how to influence the behavior of dynamic systems (like robots, airplanes, or temperature control systems) using inputs.^79^ In this context, the Routh-Hurwitz criterion offers a way to assess the stability of a linear system by analyzing its characteristic polynomial. It essentially states that for the system to be stable, all the coefficients must satisfy specific sign conditions. A crucial part of this is ensuring that the elastic (stiffness) and damping terms are both non-negative. These terms help the system return to equilibrium and prevent unbounded growth in response.

The behavior of oscillatory devices and circuits is also often discussed using concepts such as Chua’s local activity theory and the related notion of the edge of chaos.^35,214^ In this framework, steady-state operating points are classified according to their ability to locally dissipate or supply energy, and oscillatory behavior is examined by embedding the device into a circuit with passive elements and analyzing its local stability. These approaches provide valuable criteria to identify operating conditions that enable the emergence of self-sustained dynamics.

### 4.6 Relaxation Oscillations

Relaxation oscillations are a type of repetitive, periodic behavior observed in some physical, chemical and biological systems where the system slowly builds up energy or increases a variable over time and then quickly releases it. This kind of behavior can be found in firing neurons, heartbeats, certain chemical reactions, and climate cycles. Unlike smooth, sinusoidal oscillations close to the point of a supercritical Hopf bifurcation, like those of an electrical LC circuit that we have obtained in Figure 12(e, f), relaxation oscillations are characterized by two distinct time scales: a slow buildup phase and a fast release phase, or to put it differently, one variable changes much more slowly than another.^215–217^

The Van der Pol oscillator is a classic example that introduced the relaxation oscillations. In the FHN model, which is similar to the Van der Pol model, we obtain relaxation oscillations when the capacitor value, *C*_0_ is small, Figure 12(g, h). The system moves along the *u̇* = 0 line. When a fold point is reached, a jump occurs to the other stable branch by a fast, sudden transition.

Relaxation oscillations can be viewed as a phenomenon where a system switches between two thresholds. The frequency of these oscillations is mainly determined by the buildup part of the motion, and the relaxation time, either *RC*_0_ or *R*/*L*,^18^ is much longer than that of sinusoidal oscillations at the Hopf bifurcation.

Importantly, some of the most basic relaxation oscillators in electronics are based on this same principle of threshold crossing and polarity switching, implemented using simple feedback loops. A representative case, involving an Op-Amp configured as comparator and integrator, will be examined in Sec. [Sec sec7].

### 4.7 S-Type Oscillators

In the previous sections, we have discussed the FHN model which is a two-variable N-type current-voltage curve system, in which the voltage is a multivalued function of current.^30,52^ Now we turn our attention to two-variable systems with an S-type curve, as indicated in Figure 10d. S-type systems are found in many semiconductor devices,^218^ more specifically in bistable semiconductors, where the interplay between electrical instability and material nonlinearity leads to complex dynamic phenomena such as current filaments, traveling waves, and stationary patterns. All of them are central for understanding the operation of bistable semiconductor devices^219^ and have been the subject of extensive research.

Binary oxide oscillators of NbO_2_ and VO_2_ are representatives of S-type systems. They have been already introduced in Sec. [Sec sec2], see Figures 3, 6 and 9, and detailed properties are shown in Figure 14. These materials are sub-stoichiometric transition metal oxides that exhibit a temperature-driven MIT, that results in a S-type NDR as shown in Figure 6.^28,36,37,220^ As current flows through the material, Joule heating induces a phase transition from an insulating to a metallic state, rapidly increasing electrical conductance.^34,55,57,58^ This transition is followed by cooling and recovery to the insulating phase, generating periodic oscillations.^221^

**Figure 14:**
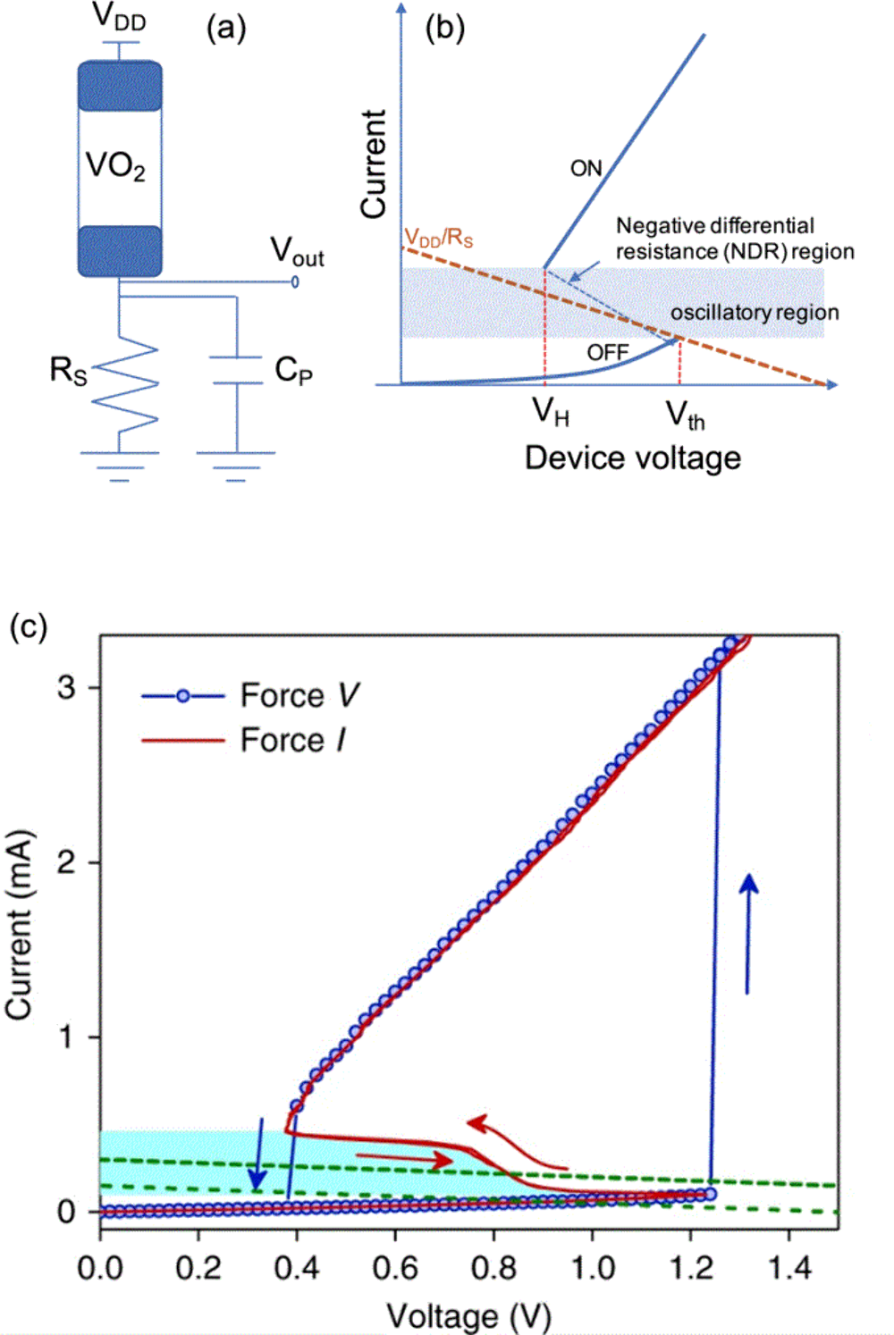
Illustration of (a) VO_2_ device with a resistor in series. (b) I–V characteristic of device. Reproduced with permission from ref 36. Copyright 2022 IEEE. (c) Typical two-terminal quasi d.c. voltage-controlled (force V) and current-controlled (force I) I-V characteristics of a VO_2_ active memristor device. A wide hysteresis loop exists in the voltage-controlled mode due to the Mott transitions (blue arrows). The same Mott transitions are manifested by an “S” shaped NDR regime (highlighted by cyan color) with a much narrower hysteresis (red arrows) in the current-controlled mode. In its resting state, the resistor load line intersects with its I-V loci outside the NDR regime (green dotted line). An input current or voltage stimulus can shift the load line into the NDR regime (green dashed line) and elicit an action potential generation (spiking). Reproduced from ref 28. Available under a CC-BY 4.0 license. Copyright 2018 The Author(s). Published by Springer Nature.

In these electro-thermal S-type oscillators, the internal thermal state of the active region, commonly referred to as the local temperature, plays a central role in determining both the existence of the NDR region and the onset of oscillatory dynamics.^222^

Within the general modeling framework adopted in this review, the local temperature can be naturally related to the internal state variable *x* used in the dynamical equations.^223,224^ In this physical realization, the current-voltage characteristics are governed by an electrical conductance that depends explicitly on the local temperature rather than on an abstract internal variable. The temporal evolution of this temperature arises from the dynamic balance between Joule power dissipation, *P*(*t*) = *i*(*t*)*u*(*t*), and heat transfer to the environment. A key parameter controlling this balance is the thermal conductance, which is distinct from the electrical conductance and quantifies the efficiency with which heat is evacuated from the device to its surroundings.

Importantly, in MIT materials, the thermal conductance may itself exhibit a significant temperature dependence, introducing an additional source of nonlinearity. This reciprocal coupling between electrical transport, local temperature, and thermal dissipation can generate, suppress, or reshape regions of NDR, thereby modulating the stability of operating points and the extent of the active oscillatory regime. While the specific physical interpretation of the internal variable and parameters must be adapted to each material system to reflect experimental conditions, the underlying nonlinear dynamical analysis remains formally analogous across different S-type oscillators.

As mentioned in Sec. [Sec sec2], VO_2_ and NbO_2_ oscillators^31–35^ are suitable for OBC paradigms^32,40,56,225–228^ These oscillators have demonstrated potential in solving complex computational tasks such as pattern recognition, associative memory, and signal processing.^1,36,37,59–66^ In the following, we address the properties of the frequency of the oscillations in relation to circuit and internal parameters, which are critical for the operation of ONNs and SNNs.

A dynamical model for S-type oscillators can be stated based on Eqs. ([Disp-formula eq4], 5) as follows^42,186,229^

57 $$C_{0}\frac{du}{dt}=I_{0}-g\left(x\right)u$$

58 $$\tau_{k}\frac{dx}{dt}=g\left(x\right)u-x$$

Here *g*(*x*) is a conductance of the nonlinear element, defined by

59 $$I_{1}=g\left(x\right)u$$

This model is represented in Figure 15a.

**Figure 15:**
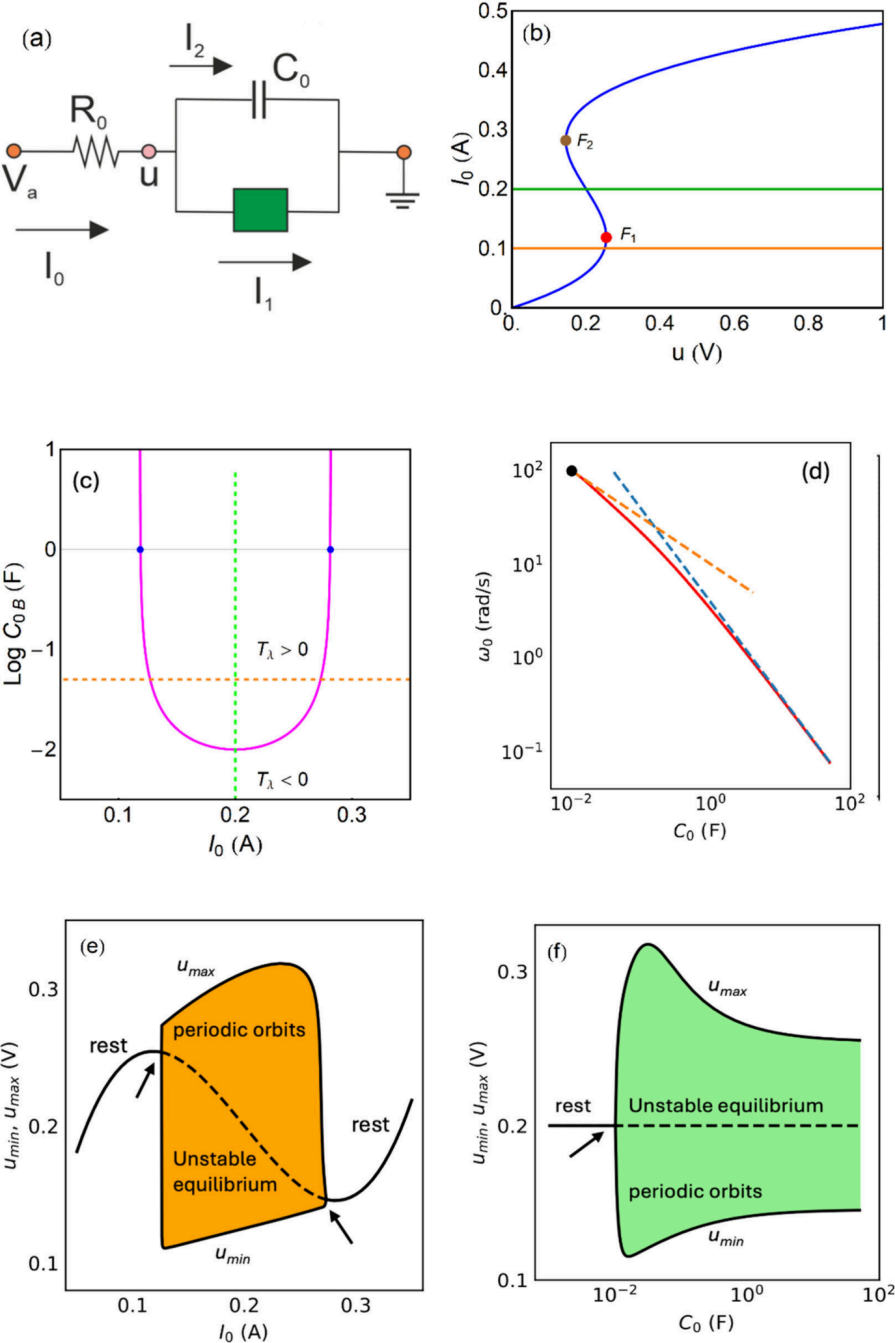
Bifurcation characteristics of the S-type system for constant applied current *I*_0_. (a) Oscillatory circuit. (b) Stationary curve *u*(*I*_0_) (blue) and constant current lines: *I*_0_ = 0.1 *A* (orange), *I*_0_ = 0.2 (green). The folding points are indicated. Parameters *a* = 50 *V*/*A* ^3^,*b* = 0.2 *A*,*c* = 1 Ω,*R*_0_ = 4 Ω, *τ*_*k*_ = 0.01 *s*. (c) Bifurcation characteristics. The pink line is the value of capacitance that gives *T*_*λ*_ = 0. The blue points are the folding points of the *u*(*I*_0_) curve. The horizontal and vertical dashed lines correspond to the projection lines of constant *C*_0_ (orange) and constant *I*_0_ (green) through which calculation of the maximum and minimum *u* values has been performed in (e) and (f) respectively, where the arrows indicate the points at which Hopf bifurcation occurs. (d) Frequency of the oscillations with varying *C*_0_ at *I*_0_ = 0.2 A. The black dot corresponds to the Hopf bifurcation point. The dashed orange and blue lines correspond to Eq. 68 and *ω*_*R*_ = ∼ 1/*C*_0_ respectively. Reproduced from Bisquert, J. et al., *J. Phys. Chem. Lett*. **2025**, *16*, 3616–3631. Copyright 2025 American Chemical Society.^42^

In S-type oscillators *u*(*I*_0_) is single valued. To illustrate a characteristic S-shape current-voltage curve the following cubic function^186,229^ is considered

60 $$u=a\left[{\left(I_{0}-b\right)}^{3}+b^{3}\right]-cI_{0}$$

in terms of parameters *a*,*b*,*c*. This is the blue curve in Figure 15b. The conductance function is

61 $$g\left(x\right)=\frac{1}{a\left(x^{2}-3bx+3b^{2}\right)-c}$$

The Jacobian matrix of Eq. 20 is

62 $$J_{X}=\left[\begin{array}{ll} -\frac{g\left(x\right)}{C_{0}} & -\frac{g^{\prime }\left(x\right)u}{C_{0}}\\ \frac{g\left(x\right)}{\tau_{k}} & \frac{1}{\tau_{k}}\left(g^{\prime }\left(x\right)u-1\right) \end{array}\right]$$

and one can obtain expressions of the trace

63 $$T_{\lambda }=-\frac{g\left(x\right)}{C_{0}}+\frac{1}{\tau_{k}}\left(g^{\prime }\left(x\right)u-1\right)$$

and the determinant

64 $$\Delta_{\lambda }=\frac{g\left(x\right)}{C_{0}\tau_{k}}$$

which is always positive because *g*(*x*) > 0, for the chosen set of a,b,c parameters. A more realistic model than Eq. 61 experimentally verified with thyristor S-oscillators has been developed.^230^

The bifurcation properties of S-oscillators have been amply described^32,186,229^ and the topic has been reviewed recently.^42^ A progressive passage occurs from harmonic oscillations [Figure 16 (a, b)] near the bifurcation point, to pulsed, triangular relaxation oscillations [Figure 16(c, d)].^215–217^ The bifurcation region with respect to (*I*_0_,*C*_0_) is given by *T*_*λ*_ = 0, and the limit cycle oscillations occur for *T*_*λ*_ > 0, Figure 15c. Experimental observations of this diagram have been reported.^187,231^

**Figure 16:**
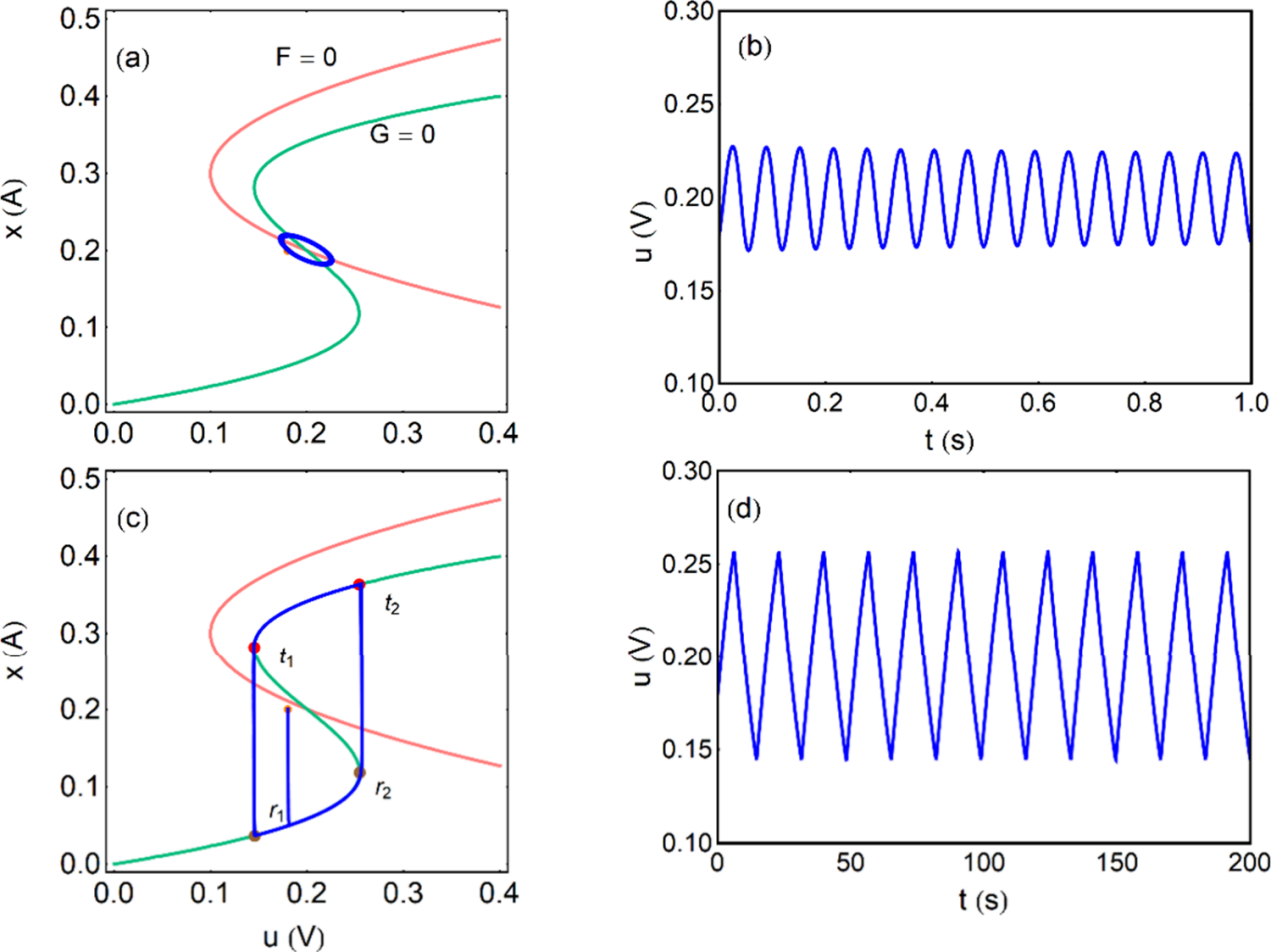
Oscillatory behavior at fixed current. Phase portrait plot, nullclines (a), (c), and trajectory of *u* variable (b), (d), of the dynamical evolution. The orange point is the initial condition. The points *r*_*i*_ = (u_i_,x_i_),*t*_*i*_ = (*u*__*i*+2_,_x_i+2_),*i* = 1,2, indicate the trajectory of relaxation oscillations. Parameters *a* = 50 *V*/*A* ^3^,*b* = 0.2 *A*,*c* = 1 Ω,τ_k_ = 0.01 *s*, *T*_*H*_ = 2 π/Δ_λ_^1/2^. (a, b) *I*_0_ = 0.2 *A*,*C*_0_ = 0.0101 *F*,*T*_*H*_ = 0.063 *s*, (c, d) *I*_0_ = 0.2 *A*,*C*_0_ = 10 *F*,*T*_*H*_ = 2.00 *s*. The initial conditions are *u*(t_0_) = 0.18 *V*,*x*(*t*_0_) = 0.2 *A*. Reproduced from Bisquert, J. et al., *J. Phys. Chem. Lett*. **2025**, *16*, 3616–3631. Copyright 2025 American Chemical Society.^42^

For the galvanostatic mode, the bifurcation can happen only when the current is between the folding points (*u*(*x*_*F*_),*x*_*F*_) of the current-voltage curve shown in Figure 15b. They satisfy

65 $$g^{\prime }\left(x_{F}\right)u\left(x_{F}\right)=1$$

and the differential resistance is

66 $$R_{d}=\frac{du}{dx}=\frac{1-g^{\prime }\left(x\right)u}{g\left(x\right)}$$

The region of NDR occurs between the folding points when *g*’(*x*)*u* > 1. In S-type oscillators the condition of fast-slow variables is inverted with respect to the N-type systems. This can be observed in the vertical turnovers of the relaxation oscillations in Figure 16d. If the capacitor *C*_0_ becomes small, oscillations cannot happen.^60,186^ The lower bound of the capacitor for oscillations to happen is the value at the bifurcation

67 $$C_{0H}=\tau_{k}\frac{g\left(x\right)}{g^{\prime }\left(x\right)u-1}$$

which is shown in Figure 15c. In Figure 15(e, f) we observe the amplitude of the oscillations as we move across the bifurcation region in the lines of Figure 15c. The frequency of small amplitude oscillations, which occur near the bifurcation point is

68 $$\omega_{\lambda }={\left(\frac{g\left(x\right)}{C_{0}\tau_{k}}\right)}^{1/2}$$

Introducing Eq. 67 we have the frequency at the Hopf bifurcation

69 $$\omega_{0B}=\frac{1}{\tau_{k}}{\left(g^{\prime }\left(x\right)u-1\right)}^{1/2}$$

In Figure 15d we observe how the oscillation frequency (red line) begins with the value ω_0*B*_ (black point). The frequency obeys Eq. 68 for a short-range of *C*_0_ values, and at higher values of the capacitance the frequency is well described by the limit of relaxation oscillations, which has the form *ω*_*R*_ = ∼ 1/*C*_0_. Similar considerations apply for the potentiostatic mode shown in Figure 17(a, b).

**Figure 17:**
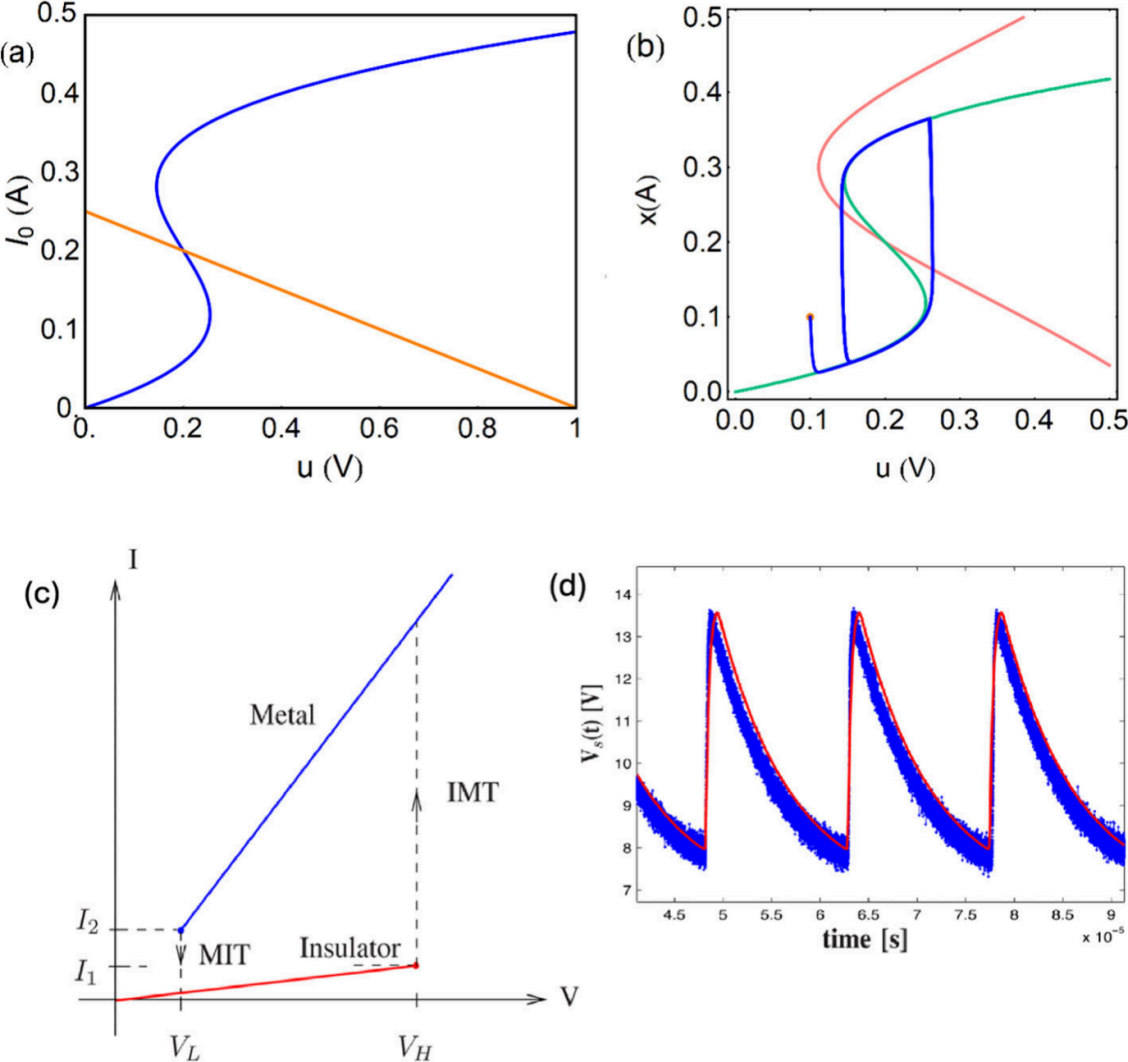
(a, b) S-type cubic model at fixed voltage. (a) Stationary curve *u*(*I*_0_) (blue) and line of constant voltage *V*_*a*_ = 1 *V* (orange) for parameters *a* = 50 *V*/*A* ^3^,*b* = 0.2 *A*,*c* = 1 Ω,*R*_0_ = 4 Ω. (b) Relaxation oscillations for C_0_ = 1 *F*,*T* = 1.95 *s*,ϵ = 2.5 × 10^–3^. Reproduced from ref 42. (c, d) S-type VO_2_ oscillator with a metal–insulator transition. (c) Current–voltage curve. (d) Simulated (solid line) and measured (noisy oscillogram) V_s_(t) waveforms. Reproduced with permission from ref 29. Copyright 2015 IEEE.

To obtain a description of an S-type relaxation oscillator, it is not necessary to formulate the full model with two differential equations, since we know that the vertical velocity *dx*/*dt* is very fast. Then a model formed by two straight lines is sufficient to obtain the period and shape of oscillations, Figure 17(c, d), provided that the load line passes between the points (*V*_*H*_,*I*_1_) and (*V*_*L*_,*I*_2_).^29,232^ The period is obtained by integration along the lines, between the turning points.^233^

Another way of obtaining an S-type oscillator is by combining two organic OECT to form an organic electrochemical nonlinear device (OEND)^77^ as shown in Figure 18(a-c), where each transistor provides one folding point. Figure 18d shows the match of the load line at constant applied voltage with the NDR of the OEND, that causes the relaxation oscillations of Figure 18(f-h). Figure 18e illustrates how the frequency of the oscillations decreases with increasing capacitance.

**Figure 18:**
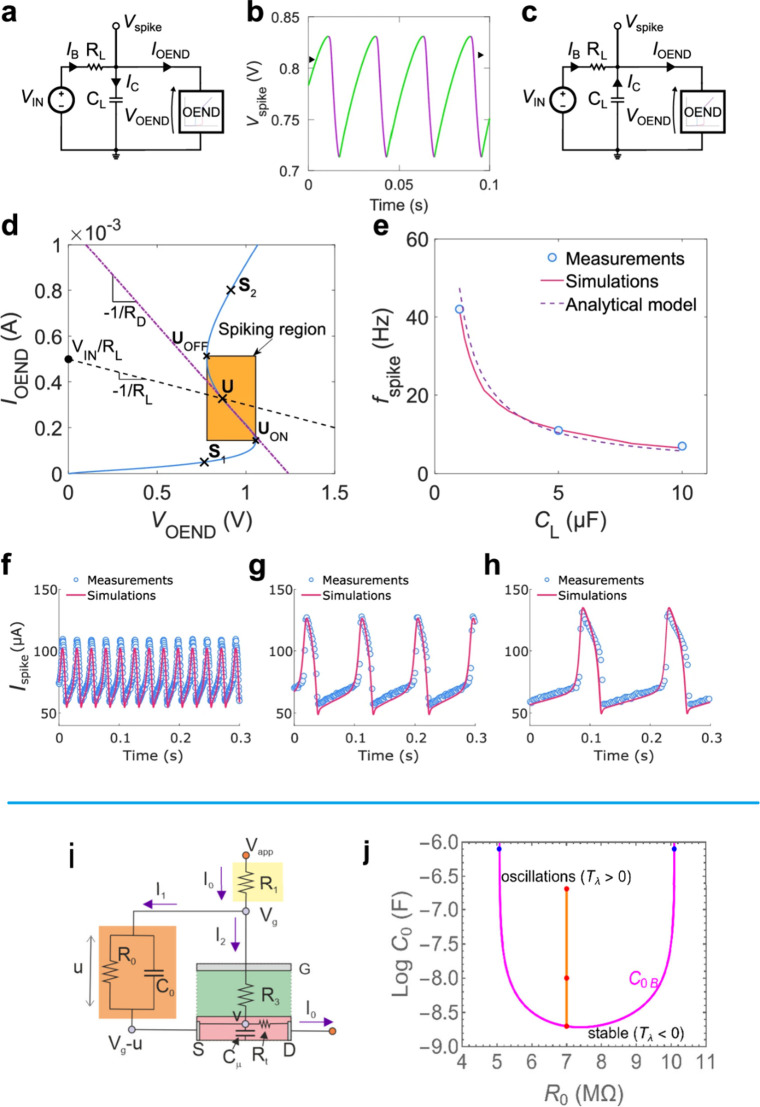
Neuron oscillator based on two organic electrochemical transistors combined. They are grouped in the block OEND. (a) Organic electrochemical artificial neuron (OAN) circuit highlighting the current partition when V_spike_ increases and the capacitor is charged. V_IN_ is the load voltage generator, R_L_ represents the load resistor, C_L_ represents the load capacitance, *I*_C_ represents the current of the capacitor C_L_, while *I*_B_ is the current flowing through R_L_. (b) Voltage oscillations (V_spike_) as a function of time. (c) OAN circuit highlighting the current partition when V_spike_ decreases, and the capacitor is discharged. (d) OEND (blue full line) and load-line (dashed line) current–voltage characteristics. When the load-line characteristic crosses the OEND characteristics in the NDR region (e.g., point U), its response bifurcates, producing voltage and current oscillations. The OEND NDR is highlighted by the dot-dashed purple line. The spiking region is defined by the upper- and lower-fold points, U_ON_ and U_OFF_, respectively. S_1_ and S_2_ are two points where the OEND characteristic shows a positive resistance (e) Spiking frequency f_spike_ as a function of load capacitor (C_L_). Symbols are the measurements; full line is calculated with numerical simulations and dashed line is calculated with an analytical model. (f) Spiking current as a function of time measured (symbols) and calculated with nonlinear transient simulations (full line) in the case C_L_ = 1 μF, (g) C_L_ = 5 μF, and (h) C_L_ = 10 μF. Reproduced from ref 77. Available under a CC-BY 4.0 license. Copyright 2024 The Author(s). Published by Springer Nature. (i) Oscillatory circuit model based on a single OECT showing node voltages and branch currents. The green-pink zone is the OECT, with gate (G), source (S) and drain (D) contacts indicated. The yellow and orange areas are externally connected elements. (j) Bifurcation diagram *C*_0_(*R*_0_). Reproduced from ref 234. Available under a CC-BY 4.0 license. Copyright 2025 The Author(s). Published by Elsevier.

Yet another application of an OECT oscillator based on the principle of a NDR is shown in Figure 18(i, j).^234^ Here a three contact transistor is converted into a two-contact limit cycle oscillator with the aid of supplementary *RC* elements. Therefore, a single OECT becomes an oscillatory system without the support of feedback amplifier elements. This model yields a bifurcation diagram (Figure 18j) similar to that of S-type oscillators, discussed above, and develops oscillations in the same way, from sinusoidal to relaxation type.

To summarize, from the analysis of nonlinear two-equation oscillatory systems, we remark that it is not possible to obtain an analytical solution of the limit cycles, though the linear analysis of the bifurcation provides many conclusions of practical value. In the general picture, Eq. 46 describes the frequency *ω*_*λ*_(***Y***_0_) of oscillations in the vicinity of the bifurcation, and away from it, relaxation oscillations, with much lower frequency occur, as illustrated by Figure 15d.

Taken together, the dynamical analyses presented in this section establish a conceptual bridge between device physics, circuit design, and oscillatory behavior, which will be exploited in the following sections devoted to materials and neuromorphic architectures.

## 5 Advanced Oscillatory Systems

While dynamic systems composed of two equations can reproduce basic oscillatory behaviors such as sinusoidal and relaxation oscillations, many natural and engineered processes, particularly in the fields of neuroscience and electrochemistry, exhibit more complex dynamics. These richer regimes often require higher-dimensional models or some other constraints to be accurately captured.^49^

### 5.1 Oscillatory Patterns in Biological Neurons

Biological information processing in the brain can be understood as a dynamic interplay of neural activity patterns that unfold over time, as discussed in Sec. [Sec sec2.3]. Recent advances in neuroscience and computational modeling suggest that mental processes are organized sequentially and hierarchically, where cognition emerges from transitions between transient but structured activity patterns known as metastable states.^112^ These states, while not permanently stable, are sufficiently persistent to support key cognitive functions such as perception, decision-making, and behavioral coordination.

The model of Izhikevich^235^ (Table 1) is very useful for generating a rich diversity of spiking patterns that can be observed in neurons in response to various stimuli, Figure 19, although strictly speaking this model is not a self-sustained oscillatory system but rather an IF one. By varying the parameters of the model and the stimuli it is possible to achieve from simple regimes such as a single spike (phasic spiking), Figure 19a, or a train of equally spaced spikes (tonic spiking), Figure 19b, to much more complicated behaviors like those of bursting in Figure 19c or even tonic bursting, Figure 19d, where sequences of rapid spike firing are interleaved with quiescent periods. Other not so regular regimes have been observed in neurons and are reproduced by the model as well. This is the case of resonant spiking in Figure 19e, in which the output of a spike requires stimulus pulses to be applied at a certain frequency, and spike-frequency adaptation^109^ in Figure 19f, where the spike frequency decreases progressively in spite of the constant stimulus applied.

**Figure 19:**
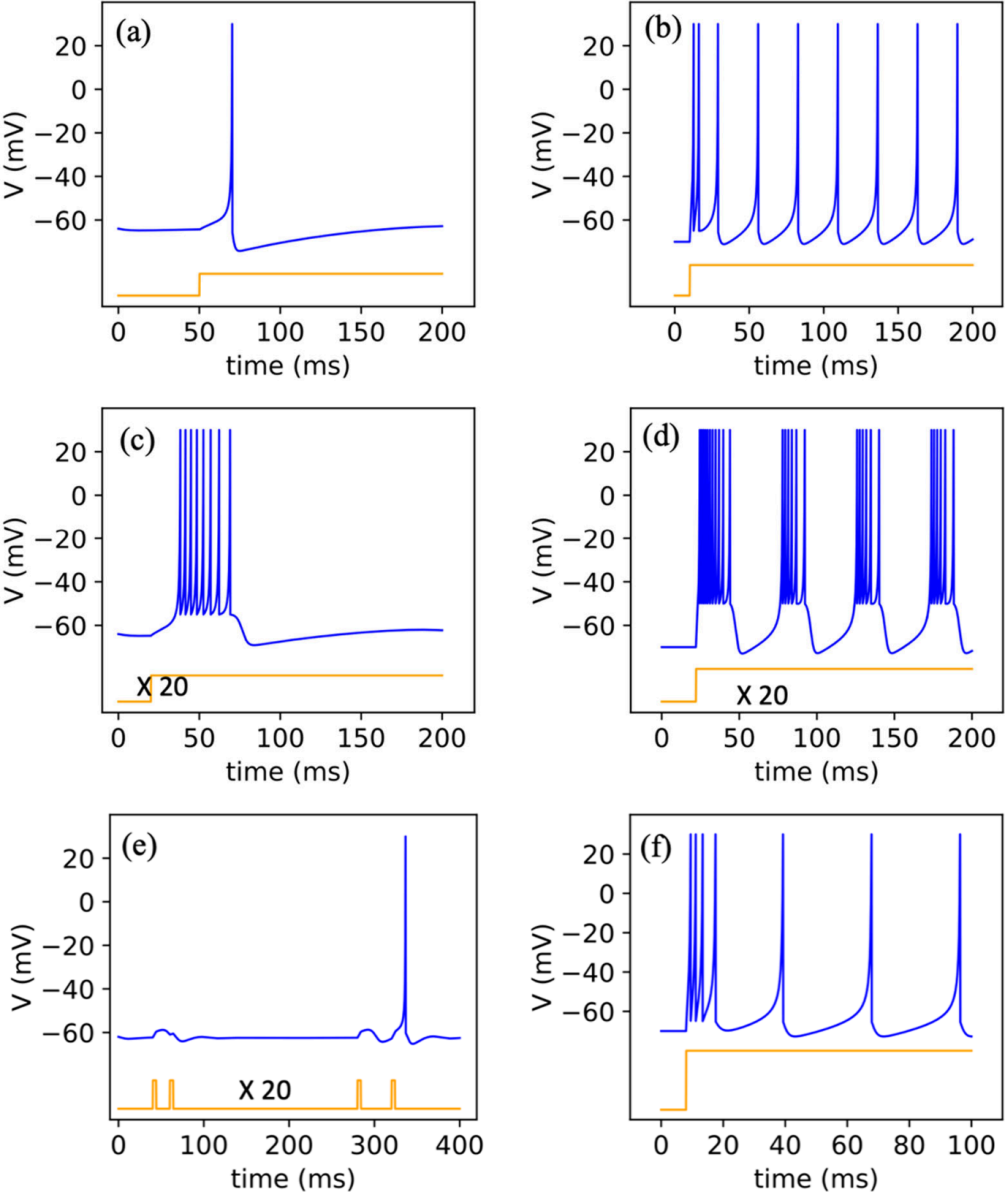
Spiking behaviors generated by the model 2 of Table 1 (blue traces). The panels illustrate six distinct dynamic regimes: (a) phasic spiking, (b) tonic spiking, (c) phasic bursting, (d) tonic bursting (e) resonator response, and (f) spike-frequency adaptation. The orange curves represent the applied input currents; in some cases, the input amplitude has been scaled by a factor of 20 to enhance visual clarity. Adapted from ref 235. Copyright 2004 IEEE. Electronic version and reproduction permissions are freely available at www.izhikevich.com.

Models such as the Izhikevich one provide a biologically plausible framework for simulating such dynamics. By capturing essential properties like spike timing, bursting, and the ability to transition between different regimes of activity, these models reveal how complex cognitive functions can arise from networks of relatively simple units. Within such frameworks, metastability plays a key role: the brain navigates through a sequence of quasi-stable configurations, or attractor-like states, allowing the system to flexibly respond to stimuli, maintain information across time, and shift between different mental modes.^236^ Hierarchical neural models further support this view by demonstrating how slower, large-scale dynamics can guide and constrain faster, local interactions.^237–239^ The duration and structure of such patterns often encode specific features of those stimuli which result from the interplay between the intrinsic properties of individual neurons and the synaptic architecture of neural circuits. In particular, bursting plays a key role in generating and maintaining metastable states. It enhances signal propagation, enables temporal coding, and facilitates synchronization across distributed brain regions.^240–242^ For these reasons, it is crucial for sensory perception, motor control, and cognitive functions, often emerging from intrinsic ion channel dynamics or network-level interactions.^108^ Bursting could be understood as a type of a more general oscillatory regime known as Mixed-Mode Oscillations (MMOs) that will be detailed in the next section. All these complex oscillations can be produced in different types of relaxation oscillators with several internal state variables^28,77^ and in electrochemical oscillators.^243^

### 5.2 Three Equations: Electrochemical Oscillators

Electrochemical oscillators have been increasingly explored as analogues of neuronal dynamics due to their intrinsic ability to generate rhythmic activity through the interplay of fast positive feedback and slow inhibitory mechanisms. Oscillations emerge in systems where the electrode potential (*E*) or surface coverage of adsorbed species (θ) governs self-reinforcing feedback loops, akin to the excitation and inhibition observed in biological neurons.^27,45–47^ In many electrochemical systems, a rise in electrode potential accelerates charge transfer reactions, further increasing the potential in a manner reminiscent of neuronal depolarization. This mechanism is particularly evident in passivation and electro-dissolution processes, where oxide layer formation alters reaction kinetics, mirroring the role of ion channel conductance in neural excitability. For example, electrocatalytic reactions, such as CO oxidation on platinum surfaces,^244,245^ demonstrate how surface coverage of reactive intermediates contributes to feedback-driven oscillations, analogous to synaptic interactions that regulate neural firing patterns.

The distinction between operating an electrochemical system at constant voltage or constant current, already commented in Sec. [Sec sec4.4], is far from trivial, as it has significant implications for both the system’s dynamics and its classification. In the context of electrochemistry, the capacitance typically represents the double-layer capacitance at the electrode-electrolyte interface, while the resistance accounts for both the intrinsic resistance of the electrolyte and any external series resistance. Based on these parameters, electrochemical oscillators are commonly classified into three major types:^180^ (i) Oscillators that function under strictly potentiostatic conditions, meaning there is no ohmic voltage drop across either the electrolyte or an external resistance; (ii) Oscillators that require potentiostatic control but include a finite ohmic resistance in the system, whether from the electrolyte or from an added external resistor; (iii) Oscillators that are capable of operating under both potentiostatic and galvanostatic conditions, showing robust oscillatory behavior regardless of whether the control variable is voltage or current.

The majority of electrochemical oscillators show N-type NDR^211^ that parallels neuronal excitability, as it enables oscillatory behavior through the dynamic coupling of charge transfer and surface reactions. These systems can yield sinusoidal and relaxation oscillations which can be easily modeled by a dynamic system with two equations. However, other arrangements show behaviors that go beyond the capabilities of low-dimensional models. This is the case of MMOs, which represent a class of complex dynamical behavior in which a system exhibits an alternating pattern of large-amplitude oscillations (LAOs) and small-amplitude oscillations (SAOs). They were first discovered in the famous BZ reaction.^122^

Another paradigmatic case is the polarographic reduction of indium (In^3+^) to metallic indium (In^0^) amalgam in thiocyanate-containing media, where MMOs have been experimentally observed.^244,246^ This reaction exhibits a N-shaped NDR region in the current-potential (I-E) curve, which emerges from electrostatic interactions and surface processes. The mechanism involves the accumulation of thiocyanate ions (SCN^–^) near the In (III) species at the electrode interface. These anions act as catalytic inhibitors and create a local electric field that repels additional SCN^–^ ions, leading to a transient destabilization. Once the repulsive force dominates, SCN^–^ ions desorb from the interface, restoring the conditions for another accumulation phase–thus generating oscillations.

The system, in terms of nondimensional magnitudes, can be described by a dynamic model involving three differential equations that was proposed by Koper^118^ (model 6 of Table 1), where *k­(u)* expresses the rate constant of the electron transfer, and θ is related to the electrode coverage with adsorbed SCN^–^ ions. The first variable, *u*, is the electrode potential, and the other two variables, *x* and *w*, account for the concentrations of two spherical diffusion layers of the same thickness, that arise in place of a single Nernst diffusion layer at the hanging mercury drop electrode. Other important parameters are the ohmic resistance, *r*, and the external voltage, *v*, from which the dimensionless current can be calculated from an equation similar to Eq. 3, here expressed as

70 $$i=\frac{v-u}{r}$$

The integration of the equations gives rise to different dynamic behaviors depending on the value of the involved parameters. Figure 20 shows the phase portraits and the time evolution of *i* for three increasing values of the ohmic resistance, *r*, indicated in the caption. In spite of small differences in *r*, the system displays totally different regimes. While for the smallest *r* value (upper plots) it yields sinusoidal oscillations, that are typical of a Hopf bifurcation, a slight increase in *r* produces a radical change of scenery where MMO appear (middle and bottom plots). They consist of large orbits in the phase portrait [Figure 20(c, e), blue traces], associated with a steady state that evolves at some point toward more closed spiral trajectories that end up again in the previous orbit. Such large orbits correspond to the highest spikes in the diagrams of the evolution time [Figure 20(d, f)] and the close orbits to small oscillations. As the figures suggest, the system is very sensitive to small variations of the ohmic resistance. Such extreme sensitivity to parameter variations is characteristic of this type of regimes. In this case, the parameter *r* allows tunning the number of small oscillations arising between large spikes. The notation *L* ^*m*^, meaning there are *m* small amplitude oscillations separated by *L* large-amplitude oscillations^178^ has been used in this respect to characterized MMOs. This way, the regimes of Figure 20(d, f) could be named as 1 ^20^ and 1 ^3^ respectively.

**Figure 20:**
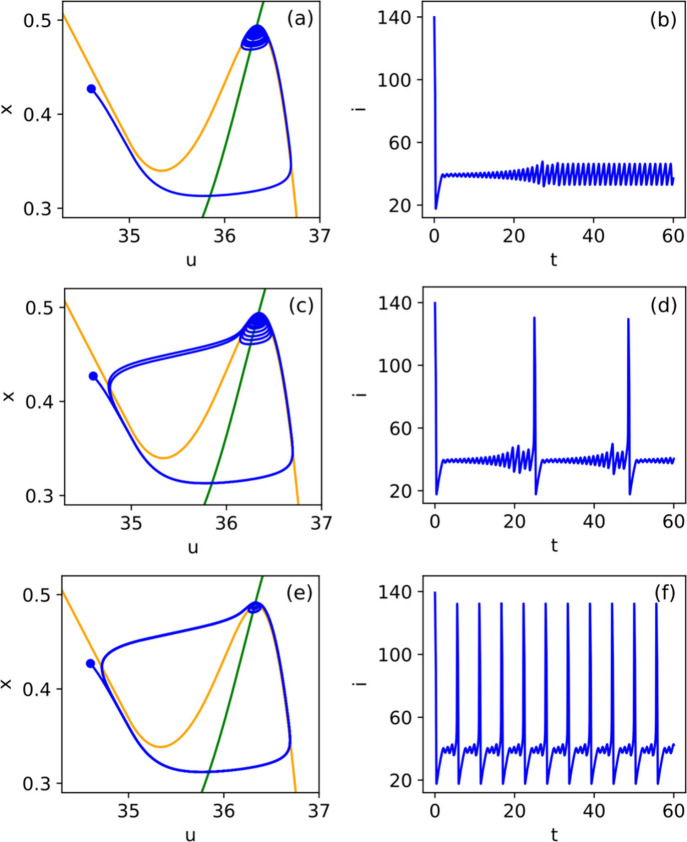
Phase portraits (a), (c), (e) and intensity vs time behavior (b), (d), (f) of the 3D system defined by Koper (model 6 of Table 1) for three different values of the *r* parameter: 0.017168 (up), 0.0171732 (middle) and 0.01724 (down). Yellow and green lines correspond to *u* and *x* nullclines respectively and the blue dot is the starting point of the trajectory. Other parameters are *v* = 37, *m* = 120, *k*_1_ = 2.5, *k*_2_ = 0.01, *e*_*d*_ = 35, *b* = 0.5, α*n* = 0.5, *e* ^*0*^ = 30.

Several mechanisms responsible for the emergence of MMOs have been proposed.^118,247–249^ One of the key points here is the interaction of fast and slow subsystems to produce a separation of time scales. This separation enables different components to dominate at different times, yielding the characteristic alternation of LAOs and SAOs. A powerful framework for studying MMOs is Geometric Singular Perturbation Theory, which decomposes fast-slow systems into lower-dimensional subsystems and examines their interactions.^178^ Within this context, *canard phenomena*, which consist of a transition from relaxation oscillations to small amplitude limit cycles, within a very narrow range of a given parameter value, play a critical role. These structures arise naturally in biological systems as well, for example in conductance-based models such as the HH system and in reduced three-dimensional versions,^249^ where the geometry of folded critical manifolds leads to sudden transitions in system dynamics, highlighting the universality of these underlying mechanisms.

MMOs are not just mathematical curiosities–they play a critical role in understanding the stability and control of processes such as corrosion, electrocatalysis, and the development of neuromorphic electrochemical devices, offering energy-efficient, bioinspired approaches to signal processing and AI.^244,250–252^ By harnessing the nonlinear dynamics inherent to these systems, researchers aim to develop hardware implementations that replicate key aspects of neuronal activity, paving the way for novel applications in brain-inspired computation and real-time adaptive systems.

### 5.3 Four Equations: The Hodgkin-Huxley Model

The spikes in natural neurons originate from the activity of voltage-gated ion channels embedded in the neuron’s membrane. These specialized protein structures regulate the movement of ions in and out of the neuron, creating the necessary electrical currents that drive neuronal signaling, as shown in Figure 2.

When a neuron receives input from other neurons through its dendrites, these signals can be either excitatory, increasing the likelihood that the neuron will fire, or inhibitory, reducing that likelihood. The neuron integrates these inputs over time, summing up excitatory and inhibitory influences from synaptic potentials, as discussed in Sec. [Sec sec2.1]. If the combined input surpasses a specific threshold, the neuron generates an action potential, sending a spike down its axon to communicate with downstream neurons. The HH model serves as the primary mathematical framework for describing all these operations in natural neurons,^13–15^ providing a detailed representation of how neurons generate and propagate action potentials using a system of four coupled differential equations (model 7 of Table 1).

The HH model describes several key physiological processes that govern neuronal spiking. Particularly, it tracks the neuron’s membrane voltage, *u*, which fluctuates according to three main ionic currents: Sodium current (Na^+^), which is responsible for the rapid depolarization phase of an action potential; Potassium current (K^+^), which contributes to repolarization by restoring the resting membrane potential; and leak current which is a small, passive current that accounts for minor ion flows, maintaining baseline electrical activity. Note in Figure 2b that the resting potential, *V*_R_, is about −70 mV. The opening of the Na^+^ channel produces a depolarizing flux by cations entering the axon, and a peak potential when the Na^+^ channel closes and the K^+^ channel opens up, returning the membrane to equilibrium but with a hyperpolarizing overshoot. The total current has positive differential resistance everywhere, as indicated in Figure 21b, while the partial sodium current contains the main NDR, Figure 21c. In the equations, *g*_*Na*,_ *g*_*K*,_ and *g*_*Leak*_ account for the maximal conductance values, and E_Na,_ E_K_ and E_Leak,_ correspond to the equilibrium potentials respectively. Besides the membrane voltage, the model introduces three gating variables, *m*, *h* and *n* that control the probabilistic opening and closing of ion channels. *m* and *h* represent the activation and inactivation of the Na channel and *n* accounts for the activation of the K channel. They adjust dynamically in response to voltage changes, influencing the flow of ions. Finally, *m*_*eq*_, *h*_*eq*_ and *n*_*eq*_ characterize steady state activation curves that can be approximated by Boltzmann-type functions, Figure 21a.

**Figure 21:**
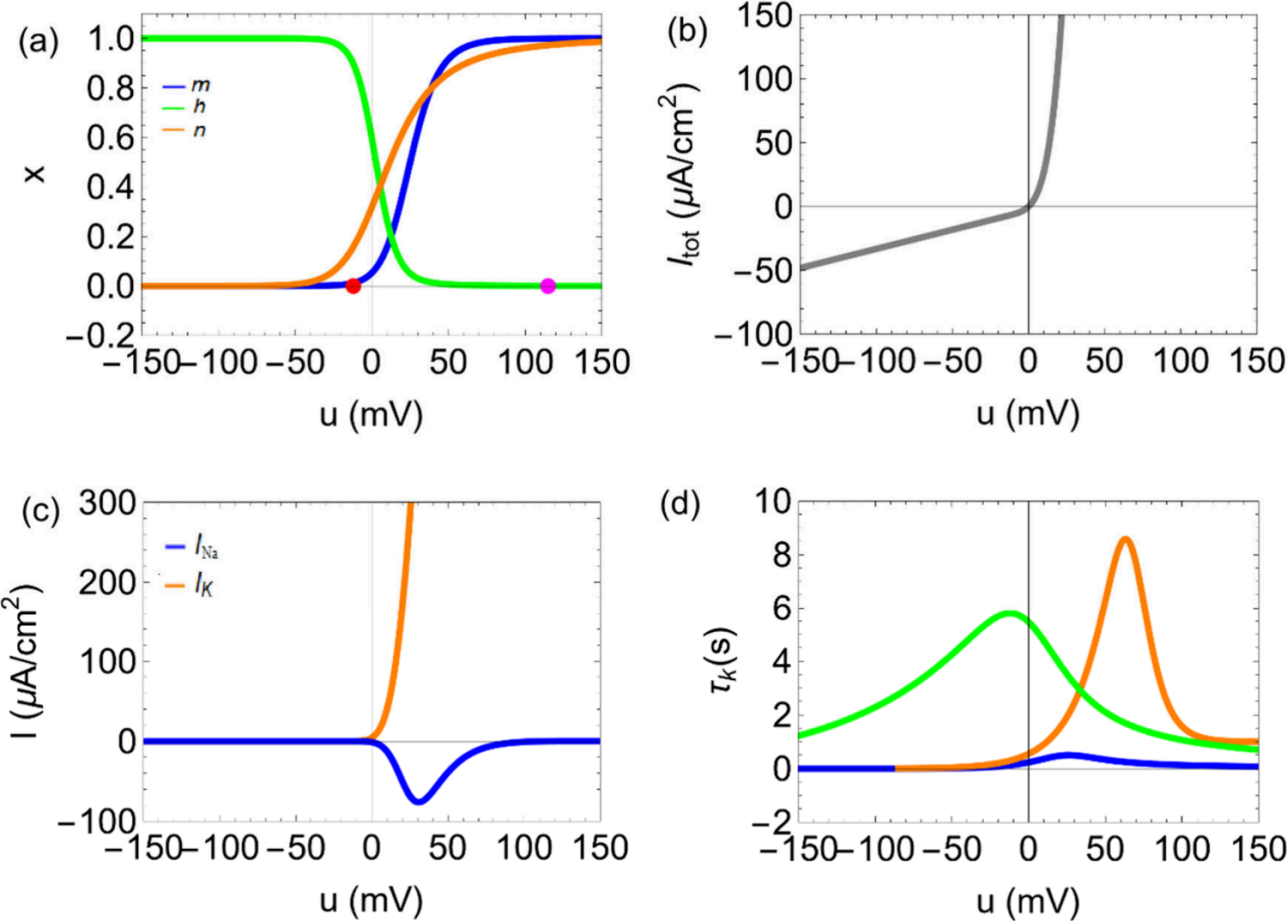
(a) Equilibrium values of the HH gating variables dependence on membrane potential *u* = *V* - *V*_*R*_. The resting potentials *E*_*Na*_ = *V*_*R*_ + 115 mV, *E*_*K*_ = *V*_*R*_ - 12 mV are indicated by dots. (b) Total equilibrium current. The conductances are *g*_*Na*_ = 120, *g*_*K*_ = 36 mS/cm^2^, *g*_*leak*_ = 0.3 mS/cm^2^ and *E*_*L*_ = *V*_*R*_ + 10.6 mV. (c) Equilibrium current of the sodium and potassium channels. (d) Relaxation times in linear scale. Reproduced from ref 253. Available under a CC-BY 4.0 license. Copyright 2024 The Author(s). Published by Advanced Physics Research.

One key point here is that while the leakage channel has an instantaneous response against voltage changes, the Na^+^ and K^+^ channels react with some delay, modulated by the times *τ*_*m*_, *τ*_*h*_ and *τ*_*n*_, whose dependence on *u* can be approximated through Gaussian functions, Figure 21d. These times, together with the capacitance value, *C*_0_, play a very important role, enriching the behavior of the system and ultimately producing the characteristic spiking behavior of neurons. By capturing the intricate temporal dynamics of voltage-gated ion channels, the HH model offers profound insights into neural excitability and has become a cornerstone in both neuroscience and neuromorphic computing.

An important feature of the HH model is its ability to exhibit complex dynamical behaviors, including bifurcations and oscillations, depending on parameters such as the applied external current, *I*_*ext*_. At low or zero applied current, the system settles into a stable resting state. This resting membrane potential corresponds to a stable fixed point in the phase space of the model. In this regime, any small perturbation to the membrane potential decays back to rest, and the neuron does not fire. However, when a sufficiently large input current is applied, the system can be pushed past a threshold, leading to the generation of an action potential. This phenomenon reflects the model’s inherent excitability. As the applied current increases, the model undergoes a Hopf bifurcation. Depending on some parameter values, this Hopf bifurcation can be either subcritical or supercritical.^254–258^ In the example of Figure 22, a Hopf bifurcation from quiescence to periodic spiking occurs at *I*_*ext*_ = 9.780 μA/cm^2^. Because at this point the bifurcation is subcritical the oscillations start abruptly with a certain large amplitude, see Figure 22a and its zoomed version Figure 22b. From this point on, the amplitude decreases progressively as the externally applied current increases, terminating at *I*_*ext*_ =154.527 A/cm^2^, where another Hopf bifurcation occurs.^254^ In this case the bifurcation is supercritical and the amplitude tends to zero as the parameter *I*_*ext*_ approaches the bifurcation point, or vice versa, it could be understood that oscillations start from zero amplitude when approaching from a quiescent to an oscillatory state.

**Figure 22:**
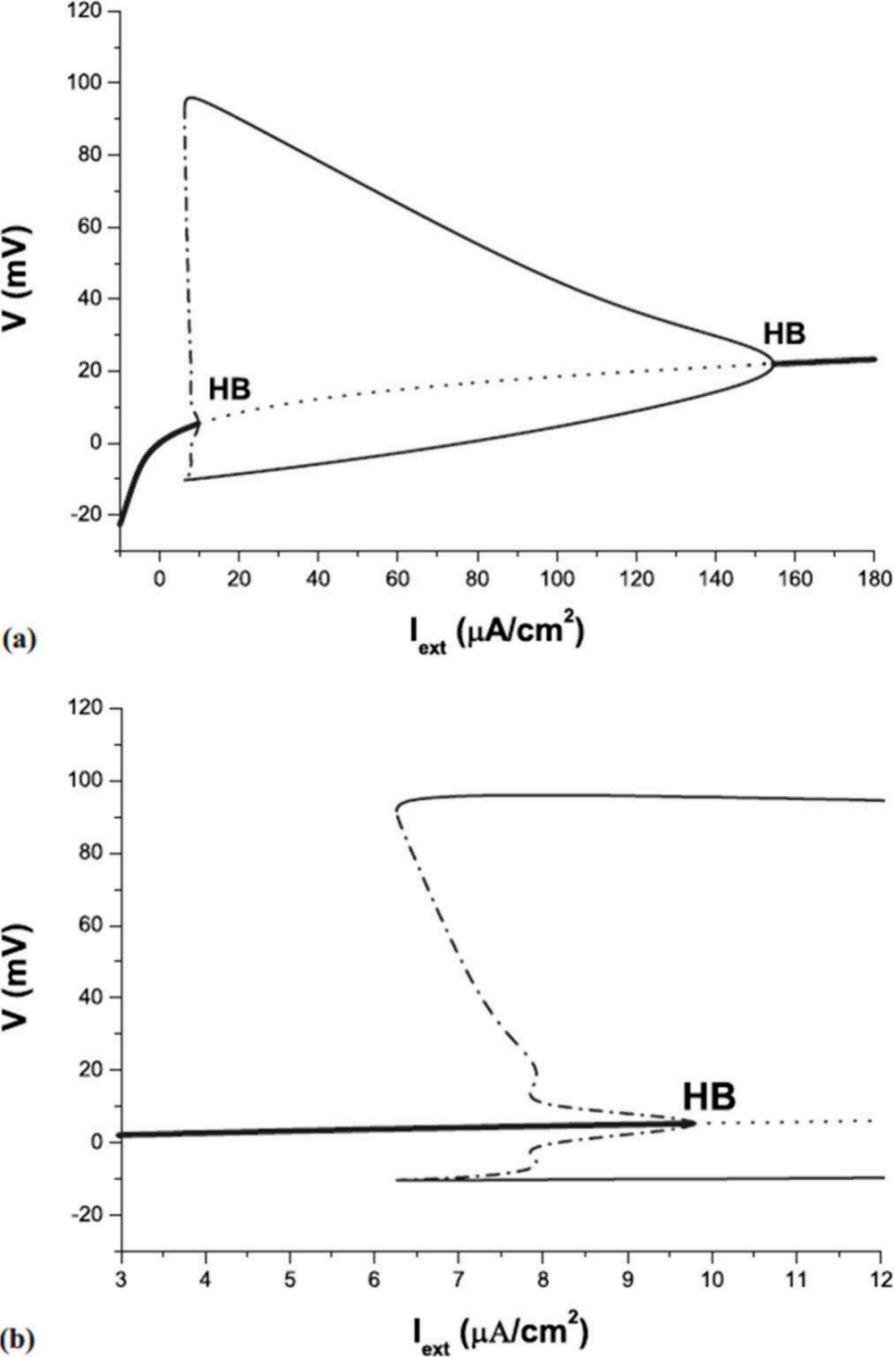
(a) Bifurcation diagram of the HH model. The thick solid lines represent stable equilibrium points, while the dotted line represents unstable ones. The maxima and minima of stable and unstable limit cycles are indicated by thin and dashed lines respectively. (b) Zoom of (a) near the left Hopf bifurcation point. Reproduced with permission from ref 254. Copyright 2008 American Physical Society.

The subcritical bifurcation is characterized by a range of *I*_*ext*_ values located before the bifurcation point (say from 6 to 10 μA/cm^2^ approximately) where stable fixed points coexist with unstable limit cycles, thus, small perturbations can push the system into a large excursion. Once the bifurcation point is crossed, the fixed points become unstable and the system jumps to stable periodic orbits, resulting in repetitive spiking. In contrast, as mentioned before, the supercritical Hopf bifurcation involves the smooth emergence of stable limit cycles from the fixed point. While this type of bifurcation can be observed in other neuronal models or modified versions of HH model, it is less typical in the original formulation. Nevertheless, both cases are related to the dynamical mechanism of neuronal excitability from quiescence to firing regimes (associated with type II excitability), where the neuron begins firing at a nonzero, finite frequency as the current crosses the bifurcation threshold.

Another important dynamical feature of the HH model is bistability because it underlies complex neuronal behaviors such as bursting or memory-like states. This phenomenon occurs within a certain range of parameter values, especially when the system is near a subcritical Hopf bifurcation. In this case, both the resting and spiking state, can be stable. This means that the neuron’s long-term behavior depends on its initial conditions; it may either remain at rest or enter a regime of continuous firing. Bistability can occur as well in other phasic environments, for instance in the context of a saddle-node bifurcation, as it will be detailed in the next section.

All the described characteristics enable biological neurons to perform highly efficient, adaptive, and robust information processing, distinguishing them from artificial neuron models, which often simplify or omit such dynamics. For this reason, many works have been focused on the development of OECTs that mimic biology-like spiking features by reproducing the whole HH circuit.^72,75,78^ However, such mathematical complexity is not always necessary. There is a long tradition of simplified models with only two differential equations that provide realistic spiking features,^14,49^ such as the FHN model, (model 1 of Table 1), and Rinzel and Wilson model^14,188,259^ (model 3 of Table 1). In this regard, we have seen in Figure 19 the variety of regimes that can be generated by the simplified two-dimensional model of Izhikevich (model 2 of Table 1). These models are more manageable than the HH model for the design of large networks. Additionally, as it will be explained in the next section, some simplified models inspired by HH, such as the Morris-Lecar model,^189^ (model 4 of Table 1), exhibit a different kind of bifurcation known as a saddle-node on invariant circle (SNIC) bifurcation. All in all, the HH model represents a simple neuron, the giant squid axon, and it can also be regarded as a simplified situation. Accurate brain neuron models may include more than ten differential equations, representing a variety of ion channels.^260^ The choice of neuron model then depends on the experimental and computational context of the particular circumstances.

### 5.4 Saddle-Node Bifurcation

Whereas complexity arises in many cases from an increased number of variables and their corresponding differential equations, in other cases it may be originated from the diversity and relative proximity of fixed points, where nullclines intersect. Such points can be repelling (unstable) or attracting (stable) and limit cycles can develop around them, thus creating attraction basins. This is the case of the saddle-node (SN) bifurcation, also known as fold bifurcation, that can appear in systems of two differential equations.^261–264^ This bifurcation occurs when two fixed points, one stable and another one unstable, approach each other, as a parameter of the system is varied, merge and finally disappear. An example (model 4 of Table 1) can be built up from a simplified version of the HH model.^49^ In fact, it is similar to the Morris-Lecar model used for describing voltage oscillations in the barnacle giant muscle fiber.^189^ The two variables of the system are *u*, the membrane voltage, and *x*, the probability of the K^+^ activation gate to be open. The other parameters that modulate the response of the system are similar to those of the HH model including the capacitance *C*_0_ and the main bifurcation parameter, the applied current *I*_0_. However, we will consider here that the time *τ*_*k*_ does not depend on *u*.

When it comes to a SN bifurcation, there is usually a third fixed point that modulates to a large extent the behavior of the system. In this context two possibilities may arise depending on if such SN annihilation occurs on a SNIC or not (SN). Figure 23 shows the phase portraits (*x* vs *u*) and voltage-time evolution for both possibilities before (top), at (middle) and after (bottom) the bifurcation, which is produced when the injected current is increased from 0 to 10 μA/cm^2^. The bifurcation occurs at *I*_0_ = 4.51 μA/cm^2^. The fixed stable and unstable nodes responsible for the bifurcation are located at the bottom-left part of the portraits (black and white dots respectively) and the third fixed point, which is unstable in this case, is placed more at the center. All of them are produced by the intersection of the *u* (yellow) and *x* (green) nullclines. At the bifurcation point, the linearized system’s Jacobian matrix has one eigenvalue equal to zero and another that is nonzero, typically negative. This indicates that the fixed point is nonhyperbolic, meaning that it does not exhibit the typical exponential behavior associated with purely stable or unstable systems.

**Figure 23:**
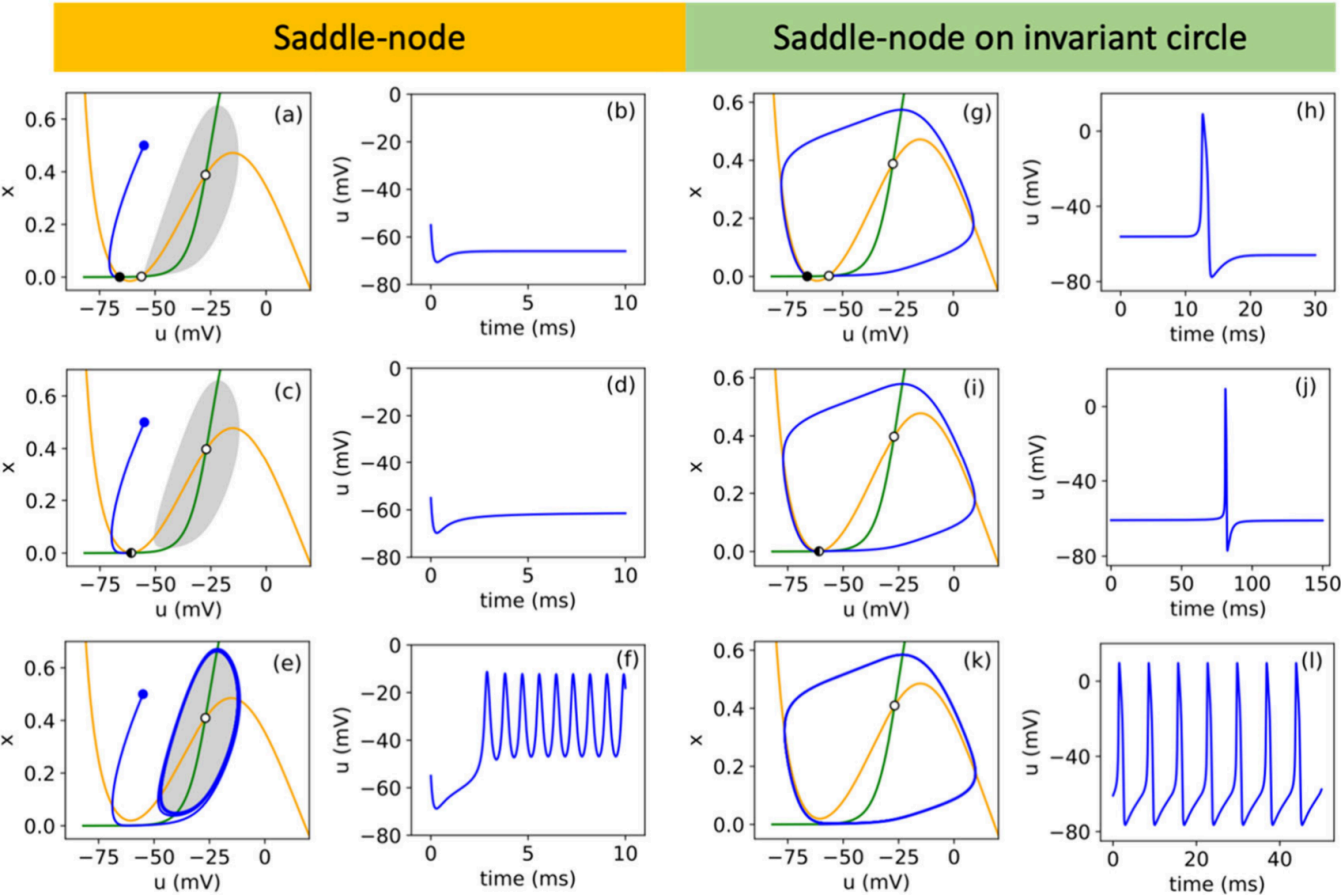
Phase portraits (a), (c), (e) and (g), (i), (k) and voltage vs time behavior (b), (d), (f) and (h), (j), (l) for a saddle-node (SN) and a saddle-node on invariant circle (SNIC) bifurcations respectively produced when varying the injected current parameter, *I*_*0*_, in the dynamical system defined by Morris-Lecar equations (model 4 of Table 1). SN and SNIC bifurcations arise from different τ values: 0.154 and 1 ms respectively. The plots represent sceneries before (*I*_*0*_ = 0 μA/cm^2^) (up), at (*I*_*0*_ = 4.51 μA/cm^2^) (middle) and after (*I*_*0*_ = 10 μA/cm^2^) (bottom) the bifurcation, which is characterized by the annihilation of the fixed stable and unstable points at the bottom-left of the portraits. Yellow and green lines correspond to *u* and *x* nullclines respectively and the blue lines represent the trajectories. They start at the same point for the SN case (blue point) and very close to the unstable bottom point, but at a slightly higher voltage, for the SNIC case. Common simulation parameter values are *C*_0_ = 1 mF/cm^2^, g_Leak_= 8 mS/cm^2^_,_ g_Na_ = 20 mS/cm^2^_,_ g_K_ = 10 mS/cm^2^, E_Leak_ = −80 mV_,_ E_Na_ = 60 mV_,_ E_K_ = −90 mV, V_1/2m_ = −20 mV, V_1/2n_ = −25 mV, *k*_m_ = 15 mV, *k*_n_ = 5 mV.

In terms of dynamics, the bifurcation captures a sudden qualitative change in behavior. It transitions from having no fixed points to having two, or vice versa, depending on the direction of the parameter change. In other words, one of the key features of the SN bifurcation is the creation or destruction of steady states, making it a fundamental mechanism underlying many nonlinear phenomena in physics, biology, and engineering.

The difference between SN and SNIC cases originates from the time, *τ*_*k*_. The fact that this time is relatively long (1 ms) for SNIC causes the trajectories (blue lines) in the phase portraits to attach more to the *u*-nullcline thus giving rise to large single voltage spikes before the bifurcation. This is the case of heteroclinic trajectories starting nearby the bottom unstable point but at slightly higher voltages and finishing at the stable point. This constitutes the invariant circle. At the bifurcation, both points merge and such trajectories become homoclinic, meaning that they start and finish at the same point. In principle, a trajectory starting at the exact bifurcation point would take infinite time to accomplish the complete circle. However, once the bifurcation has been overcome the system starts oscillating, producing spikes with a certain frequency. During this periodic process, the slow (nonspiking) part of the oscillation corresponds to a very short trace in the phase portrait near the point where the stable and unstable points had merged while the fast-spiking part corresponds to the rest of the cycle. Thus, even though the system has surpassed the bifurcation and the two fixed points causing it have disappeared, it still “remembers” the point where the bifurcation occurred and slows down nearby. The increase of the injected current, *I*_*0*_, moves away the trajectories from the merging point and thus increases the passage speed at that place. Therefore, the quiescent part shortens and the spiking frequency increases. This explains why this type of oscillations start from zero frequency, at difference from what occurs in a Hopf bifurcation, where oscillations start at certain finite frequency. This spiking behavior arising from a SN bifurcation in an invariant circle coincides with Type I excitability of neurons according to the classification of Hodgkin.^13–15^

The phase portrait scenario is different with a shorter relaxation time. For *τ*_*k*_ = 0.152 ms (left part of Figure 23, SN case) the third fixed point at the center of the phase portrait produces a basin of attraction (shaded areas). Therefore, any trajectory starting within that basin will end up in a limit cycle at the periphery of the basin, thus giving rise to voltage oscillations regardless of the SN bifurcation. However, if the trajectory starts out of the basin, it will go quickly to the stable node as long as the bifurcation has not occurred yet, thus producing a stable state. This is a case of bistability between a resting and a spiking state. Once the bifurcation is overcome, there are no stable points and all the trajectories end up in the limit cycle producing an oscillatory behavior with a finite frequency. This behavior coincides with Hodgkin Type II excitability.

## 6 General Properties of Oscillators

Having discussed many self-sustained oscillators representing neuron models in the previous section, we now examine some shared properties of certain classes of models, to show different resources to describe oscillatory devices and their combinations.

### 6.1 Local and Global Analysis

Lyapunov conditions provide a framework for analyzing the stability of equilibrium points in dynamical systems by considering how small perturbations behave in their vicinity, as explained in Appendix 2. If a point is stable according to Lyapunov’s criteria, then small deviations from that point decay over time, returning the system to equilibrium. However, the emergence of limit cycles (closed trajectories in phase space that represent sustained oscillations) cannot always be fully captured by such local analysis. While a limit cycle may surround a stable point or arise near a bifurcation, it inherently involves trajectories that venture far from the immediate neighborhood of the equilibrium. We have observed some examples of these features in the SN bifurcation in Sec. [Sec sec5.4], where the trajectories in phase space depend on a third fixed point away from the merging fixed points. In such cases, the linear approximation used in local stability analysis, such as linearization around fixed points, may no longer be valid. As a result, the presence, shape, and behavior of limit cycles often depend on the global structure of the dynamical system, not just local properties. To fully understand these oscillatory dynamics, one must often consider the nonlinear and global features of the system rather than relying solely on local Lyapunov-based analysis.

### 6.2 Conductance-Activated Models and Relaxation Time

We remark a general structure of the equations for oscillatory systems that represent voltage-gated ion channels. The current across a channel has the basic form

71 $$I_{ch}=xg_{x}\left(u-E_{0}\right)$$

Here *g*_*x*_ is a conductance, *x* is the gating variable (0 ≤ *x* ≤ 1), and *E*_0_ is an equilibrium potential. Note that this form appears in the models 3 (Rinzel-Wilson), 4 (Morris-Lecar) and 7 (HH) of Table 1, and occurs in a large variety of related models as described by Izhikevich.^49,240^ All these models have the common property that the gating variable activates a high conductance state, hence we may call them *conductance-activated* models. In contrast, models 1 (FHN) and 2 (Izhikevitch) in Table 1 are not conductance-activated. The memory variable, instead, is a current, associated to the current in the inductor.

Eq. 71 represents, with the help of the gating variable, *x*, the important property of rectification, where the current is much larger in one voltage range than in the opposite. More specifically, Eq. 71 describes the phenomenon of *ionic current rectification*, where the flow of ions through channels or pores behaves differently depending on the direction or rate of the applied voltage.^265^ Such rectifying channels, found in cell membranes, allow more ion flow in than out, depending on conditions like ion concentration and speed of measurement. That behavior is important for how cells control their electrical activity and is a key part of how neurons send signals, as described in the HH model. This happens not only in natural protein ion channels in living organisms but also in synthetic nanopores made in laboratories.^266^ These artificial nanopores that mimic biological channels achieve rectification by means of an uneven pore shape or surface charge, which influences in the way ions move through them.^267^

In the mentioned models Eq. 71 is supplemented by a chemical inductor relaxation equation for *x* of the type (see Eq. [Disp-formula eqA1.2]):

72 $$\tau_{k}\left(u\right)\frac{dx}{dt}=x_{eq}\left(u\right)-x$$

The function *x*_*eq*_, with a sigmoidal or another similar form (Eq. [Disp-formula eqA1.3]) defines the activation of rectification at some threshold voltage *V*_*B*_. Therefore, as discussed in Appendix 1, Eq. 72 introduces hysteresis, i.e. a delay or difference in a system’s response depending on how fast or slow it is measured. This has been found, for example, when measuring the I-V characteristics of channel systems that conduct electricity through ions.^268^

Note that a model with the dynamical structure of Eqs. ([Disp-formula eq71], 72) is quasi-linear in both variables *u*,*x*, in the sense that the nonlinearity occurs in the function *x*_*eq*_(*u*) of the chemical inductor and possibly in the relaxation time *τ*_*k*_(*u*). This has been discussed in Appendix 1, since memristors enable this dynamical structure in the models of the type conductance-activated quasi-linear memristor model (CALM).^81^ In addition to the rectification property of Eq. 71, the kinetic relaxation time *τ*_*k*_(*u*) is an essential component of the model as it governs the rate at which transitions occur between different conductive states. This characteristic time determines how quickly a system can respond to changes in stimuli, and it is strongly influenced by the applied voltage–meaning that as the voltage varies, so does the kinetic relaxation time (see Figure 21d). It is, in fact, a critical parameter in neuron models, as we have shown in Figure 23.

Although the previous features produce rectification and hysteresis effects, neuron models usually require an additional ingredient, discussed before, that is a NDR. The mechanism for introducing this ingredient takes different forms depending on the model equations. In two-variable models 1-4 of Table 1, the NDR occurs in the fast channel (*u* variable), while the *x*-channel of the chemical inductor has a positive resistance. In model 7 of Table 1 (HH), the NDR is formed by the combination of an activation (*m*) and a deactivation (*h*) variables, thus the Na^+^ channel first opens and then closes, Figure 21c, with the variables having different relaxation times, see Figure 21d.^253^

The structure of the NDR in conductance-activated models can be further clarified by the following system of equations, that describes an oscillatory voltage (Figure 24c) in a fluidic nanochannel.^269^

73 $$C_{m}\frac{du}{dt}=I_{tot}\left(u\right)-g_{H}\left[r+b+pW\left(u\right)\left(1-r\right)\right]u$$

74 $$\tau_{k}\frac{dp}{dt}=P_{eq}\left(u\right)-p$$

75 $$P_{eq}=\frac{1}{1+e^{-\left(u-V_{P}\right)/V_{mp}}}$$

76 $$W=\frac{1}{1+e^{\left(u-V_{W}\right)/V_{mw}}}$$

**Figure 24:**
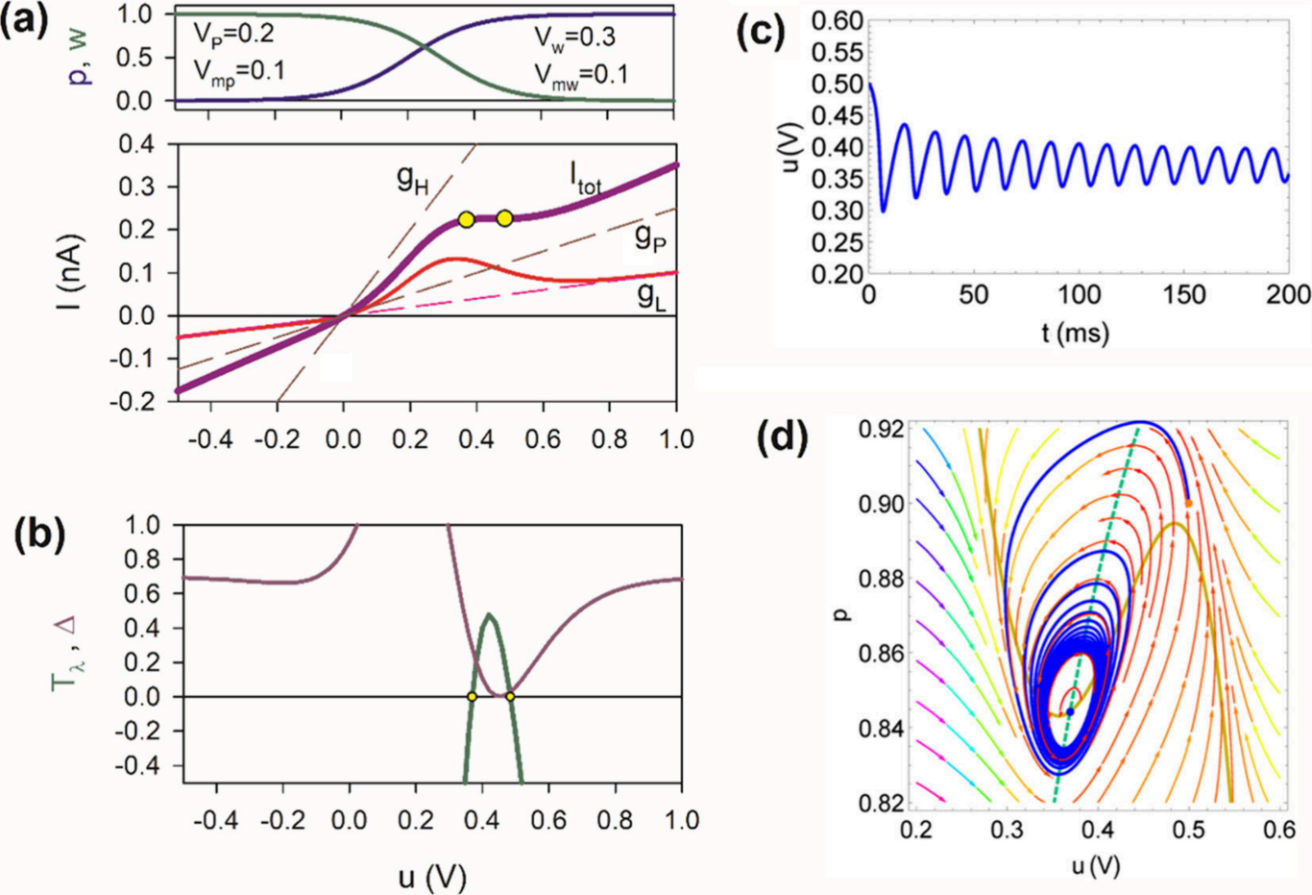
Oscillations in the rectifying nanopore model. (a) Top: activation, *P*_eq_, and inactivation, *W*, functions as a function of the voltage variable, *u*. Bottom: Total current-voltage characteristics (thick purple curve) obtained by adding the contribution of the leak current *g*_*p*_ (dashed line) to the function *g*_*H*_ [*r* + *P*_*eq*_ *W* (1 - *r*)]*u* (red curve). Other reference dashed lines corresponding to low *g*_*L*_ and high *g*_*H*_ conductivities are indicated. The yellow points are the Hopf bifurcations that are obtained from (b) by the trace *T*_*λ*_ = 0, causing oscillations when the determinant Δ > 0. (c) Voltage evolution with time. (d) Phase portrait plot showing the nullclines, trajectories, and vector velocities. The *f*-nullcline is the yellow line [associated with Eq. 73] the *h*-nullcline is the green line [associated with Eq. 74]. The orange point is the starting condition. The blue point is the fixed point at the nominal potential. The blue line indicates the evolution of the voltage until it settles into permanent limit cycle oscillations. Parameters: *C*_*m*_ = 0.1 mF, τ_0_ = 5 ms, V_mp_ = *V*_*mw*_ = 0.1, *V*_*P*_ = 0.2, *V*_*W*_ = 0.3, *b* = 0.25, *r* = 0.1, *g*_*H*_ = 1 *nS*. Reproduced from ref 269. Available under a CC-BY 4.0 license. Copyright 2025 The Author(s). Published by American Physical Society.

This is, in fact, another two-equation model, with the usual conductance appearing in Eq. 73, which is activated by variable *p* that is driven by the relaxation Eq. 74. In addition, the function *W*(*u*) produces the inactivation of the conducting channel in Eq. 73, at a certain onset voltage *V*_*W*_. Figure 24a (top) shows the two equilibrium functions of Eqs. ([Disp-formula eq75] and 76). The thresholds and the steep of the transitions are controlled by *V*_p_, *V*_W_ and *V*_*mp*_, *V*_*mw*_ parameters respectively. The term *b* in Eq. 73 corresponds to the leak current and it is defined by a conductivity *g*_*p*__,_ so that *b* = *g*_*p*_/*g*_*H*_, and *r* = *g*_*L*_/*g*_*H*_ where *g*_*L*_ and *g*_*H*_ correspond to low and high conductivities respectively. The leak current makes the total current (thick purple curve in Figure 24a bottom) have a positive derivative everywhere. Hence the Hopf bifurcation occurs by a “hidden” NDR^270^ illustrated by the red curve in the same figure.

### 6.3 Phase Response Methods and Network Synchronization

A general property of self-sustained oscillators is that they evolve over time toward a stable, periodic trajectory in their phase space, a limit cycle, regardless of their initial conditions. Once on this cycle, the system’s behavior becomes predictable and repetitive, making it an ideal framework for modeling biological rhythms such as neuronal firing, circadian cycles, or cardiac rhythms. While analytical trajectories of nonlinear oscillators can usually not be found, a number of methods denominated phase response techniques become highly valuable to describe significant aspects of oscillators when they are perturbed by an external impulse or coupled in interacting systems.^16,20,51^

At the heart of each limit cycle oscillator is an internal clock, which can be described by a phase variable ϕ, typically ranging from 0 to 2π. This phase advances uniformly over time in the absence of external input, governed by the simple differential equation

77 $$\frac{d\phi }{dt}=\omega$$

where ω is the natural angular frequency of the oscillator. The phase thus acts as a compact representation of the oscillator’s position along its cycle, providing a powerful tool to study how the system behaves and interacts with its environment. The phase representation of the FHN oscillator is shown in Figure 25(a, b).

**Figure 25:**
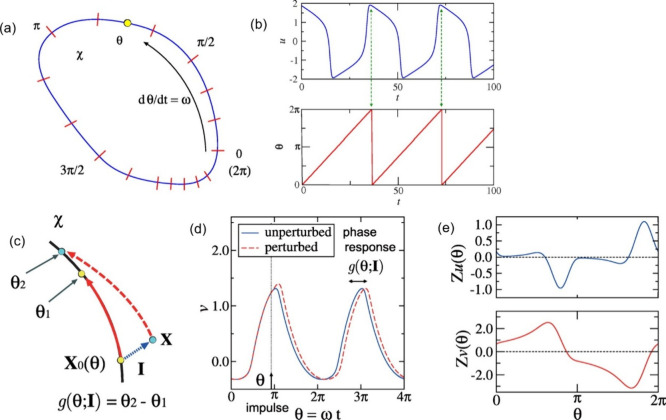
Phase response methods on the FHN model. (a) Definition of the phase of the limit cycle. The phase θ is identified with the time multiplied by the frequency, *i.e. θ* = *ωt*, yielding a constantly increasing phase variable and nonuniform scales on the limit cycle. (b) Time series of the variable *u* and phase θ of the limit-cycle orbit. (c), (d) Phase response of the oscillator to an impulsive stimulus (state space and time sequence). (e) Phase sensitivity function ***Z*** (θ) = (*Z*_*u*_ (θ),*Z*_*v*_ (θ)) of the FGN model. Reproduced with permission from ref 271. Copyright 2015 Taylor and Francis.

A crucial aspect for understanding the utility of this approach is knowing how these oscillators respond to external perturbations. This is where Arthur Winfree’s phase response method becomes highly valuable.^20^ Winfree introduced a technique to quantify how a brief stimulus, applied at different points along the cycle, shifts the oscillator’s phase, as indicated in Figure 25(c, d). This relationship is captured by the Phase Response Curve (PRC), denoted by ***Z*** (θ), which maps the oscillator’s phase shift at the time of stimulus, Figure 25e.^271,272^ Mathematically, when a small external perturbation Δ ***I*** is applied to an oscillator described by the vector ***X***, the resulting phase shift Δθ can be approximated as Δθ = ***Z*** (θ)•Δ ***I***, where ***Z*** (θ) serves as a sensitivity function. The PRC provides a deep insight into the oscillator’s dynamics, revealing when it is most susceptible to being advanced or delayed. This response profile is critical for understanding how oscillators synchronize with each other or entrain to periodic external inputs (Sec. [Sec sec6.4]), such as a circadian clock synchronizing to the light-dark cycle. Methods to calculate the phase sensitive function have been developed by Ermentrout^16,273,274^ in relation to the modeling of neural systems.

Phase response methods offer a powerful approach for understanding the dynamics of systems made up of multiple interacting oscillators.^51,275^ Winfree^19^ observed that oscillators of a population, coupled with a certain strength, can adjust their phases between each other. When the coupling strength ϵ is weak, each oscillator tends to follow its intrinsic rhythm. But as ϵ increases beyond a critical threshold, a collective phenomenon emerges: synchronization.

Consider a population of *N* oscillators each described by the vector ***X***_*j*_. The dynamics of each oscillator is described by^271^

78 $$\frac{dX_{i}}{dt}=F_{j}\left(X_{j}\right)+\epsilon \frac{1}{N}\overset{N}{\underset{k=1}{\sum }}H\left(X_{k}\right)$$

The term ***F***_*j*_(*X*_*j*_) represents the unperturbed dynamics of the oscillators as in Eqs. ([Disp-formula eq6], 7). The last term is the weak global coupling so that all oscillators feel the common mean field ∑_*k* = 1_^*N*^ ***H*** (***X***_*k*_)/*N*. As an example, this approach is useful for studying a population of electrochemical oscillators coupled by a common resistor.^250^

By applying the phase reduction to Eq. 78 and averaging, the following *globally coupled phase model* is obtained^271^

79 $$\frac{d}{dt}\phi_{i}\left(t\right)=\omega_{j}+\epsilon \frac{1}{N}\overset{N}{\underset{k=1}{\sum }}\Gamma \left(\theta_{j}-\theta_{k}\right)$$

where *ω*_*j*_ is the natural frequency of the *j* ^th^ oscillator and

80 $$\Gamma \left(\varphi \right)=\frac{1}{2\pi }\int_{0}^{2\pi }Z\left(\varphi +\psi \right)\cdot H\left(X_{0}\left(\psi \right)\right)d\psi$$

is the phase coupling function. In particular, when the phase coupling function is given by the lowest order Fourier term as Γ­(φ) = −sinφ, the model becomes the well-known Kuramoto model^51^ given by

81 $$\frac{d}{dt}\phi_{i}\left(t\right)=\omega_{j}-\epsilon \frac{1}{N}\overset{N}{\underset{k=1}{\sum }}\sin\left(\theta_{j}-\theta_{k}\right)$$

Kuramoto extended Winfree’s results and proposed the solvable coupled oscillator model (81) that exhibits a collective synchronization transition.^276,277^ A subset of oscillators begins to lock in phase, and their dynamics become coordinated. This emergence of synchronization from individual variability is one of the most striking features of the model and captures real-world behavior observed in biological and physical systems.^160^

The Kuramoto model has found wide applications across disciplines.^276^ In neuroscience, it helps explain how networks of neurons can synchronize their firing patterns.^278^ In chronobiology, it models how circadian cells synchronize within an organism or how populations entrain to environmental cues like light.^279^ In engineering, it informs the design of distributed systems that need to maintain timing, such as sensor networks or power grids.^161^

In summary, the PRC method simplifies complex multidimensional oscillator models to a single scalar variable representing the phase of oscillation.^20,280^ In practical terms, the PRC has been applied to many biological systems. For instance, in circadian biology, researchers use brief light pulses to probe how the internal clock responds depending on the time of the day the light is delivered.^281^ This generates a characteristic PRC, which may show phase delays when light is applied in the evening and phase advances when applied in the early morning. Overall, the framework of limit cycle oscillators and the Winfree phase response method offers a unifying language for studying rhythmic systems.

### 6.4 Entrainment

Another way of referring to the phenomenon of synchronization is *entrainment*. This term has been mostly used in the framework of biological systems, in which two or more oscillators interact–often through physical or chemical means–in such a way that they become synchronized in their rhythms.^282^ Entrainment plays a crucial role in coordinating processes across various levels of organization, from molecular mechanisms within a single cell to physiological functions across entire organisms and the swarming of populations.^283^

Entrainment by a periodic external input is a fundamental mechanism by which oscillatory systems synchronize their internal rhythms to regular signals from the environment.^24^ In the context of neural networks, this process plays a critical role in coordinating neural activity both within and across brain regions.^284^ Neural oscillators, ranging from single neurons to entire populations, can lock their firing patterns to the frequency and phase of an external input, such as a rhythmic sensory stimulus, electrical stimulation, or an ongoing oscillatory input from another brain area.

This entrainment helps structure neural dynamics in ways that are essential for cognitive and behavioral functions. For instance, entrainment to periodic auditory or visual inputs is observed in attentional tasks, where cortical neurons align their activity with rhythmic stimuli to enhance perception and reaction timing. Similarly, neural entrainment to speech rhythms is thought to facilitate language comprehension by synchronizing neural excitability with the temporal structure of speech.

From a modeling perspective, neural populations are often represented using phase oscillator models, such as the Kuramoto model, which can incorporate external periodic forcing to study entrainment phenomena. These models help explain how coherent rhythms can emerge in large-scale brain networks and how phase-locking to external stimuli can arise under different coupling strengths and noise conditions. A key mathematical tool in this analysis is the PRC which describes how an oscillator’s phase is shifted by a perturbation, as shown in Sec. [Sec sec6.3], allowing precise prediction of entrainment dynamics.^284^

### 6.5 Forcing the Oscillators: Impedance Spectroscopy

We have commented in Sec. [Sec sec6.3] the significance of observing the effect of a small forcing perturbation on the evolution of the phase of the oscillator. Now we turn to the analysis of a small forcing current Δ*I* with frequency *ω*_*f*_ over the complete system of differential equations that define the change of the phase space vector ***X***. The dynamical equations including such perturbation have been already discussed in Sec. [Sec sec4.3] (see Eq. 19). There we remarked that the system properties could be analyzed in terms of the linear impedance, *Z*(*s*), where *s* = *i ω*_*f*_. This approach connects with a widely used technique in many classes of systems, which is the method of impedance spectroscopy, that utilizes equivalent circuits and complex plane plots as the main methods of analysis.^285–287^ Hence, impedance spectroscopy becomes an important method for the characterization of self-sustained oscillators. Furthermore, we note that the equations governing the impedance response contain the linearized equations of the oscillatory system (Eq. 39) as the particular case in which the external forcing is constant, Δ*I* = 0. This allows the impedance spectroscopy method to determine the bifurcation properties and classifying the oscillatory conditions, as described in the work of Koper and co-workers.^45,288,289^

Let us explain the overall connection between impedance measurements and bifurcation properties taking the example of the two-equation oscillator.^52,195^ The impedance is obtained from Eq. 19 and the result is shown in Eq. 26. We have emphasized the chemical inductor model of Figure 13a for positive values of resistance and inductance (Figure 13b), but the same impedance model and equivalent circuit are valid when resistance and inductance are negative [Figure 13(c, d)].

Translating Eq. 19 to the frequency domain and using the Cramer’s rule, the impedance function can be written as

82 $$Z\left(s\right)=\frac{\Delta u}{\Delta I_{0}}=\frac{1}{\det\left\{J_{X}-sI\right\}}\frac{1}{C_{0}\tau_{k}}\left(1+s\tau_{k}\right)$$

The determinant can be expanded as

83 $$\det\left\{J_{X}-sI\right\}=s^{2}-T_{\lambda }s+\Delta_{\lambda }$$

Note that this is the characteristic polynomial equation of the Jacobian matrix *J*_*X*_, and it is, in fact, similar to the eigenvalue Eq. 41, since the linear matrix for the self-sustained oscillator *J*_*X*_ is the same (Eq. 21). However, there is a difference originated by the forcing term in Eq. 19 which makes the system operates at different conditions. While the system driven by Eq. 40 oscillates by means of a limit cycle and the eigenvalues, λ, can have both real and imaginary parts, the system driven by Eq. 19 is forced by an external current of frequency *ω*_*f*_.^54^ Thus *s* = *iω*_*f*_ can be compared to λ if imaginary values of the frequency are introduced, to obtain the general expression (Eq. 47) that provides the criteria for bifurcation.^27,290–292^

The general expression of the impedance in Eq 82 is

84 $$Z\left(s\right)=\frac{\Delta u}{\Delta I_{0}}=\frac{1}{C_{0}\tau_{k}}\frac{\left(1+s\tau_{k}\right)}{s^{2}-T_{\lambda }s+\Delta_{\lambda }}$$

It is the same function as Eq. 26. The spectra are shown in Figure 26 for the FHN model previously described in Figure 12. For a stable point ***Y***_0_ the spectrum (blue curve in Figure 26) is the chemical inductor of Figure 13b, while in the oscillatory region the spectrum (green curve in Figure 26) is that of Figure 13c. This last form is most relevant for oscillations in a Hopf bifurcation. The spectrum starts at positive DC resistance, but it makes an excursion into the NDR region at a finite frequency ω_1_ > 0. Experimental examples from an electrochemical system are shown in Figure 27. This elementary model of two equations provides a basic framework for analyzing more complex systems, as the HH model that is characterized by the presence of three chemical inductors.^290,293–295^

**Figure 26:**
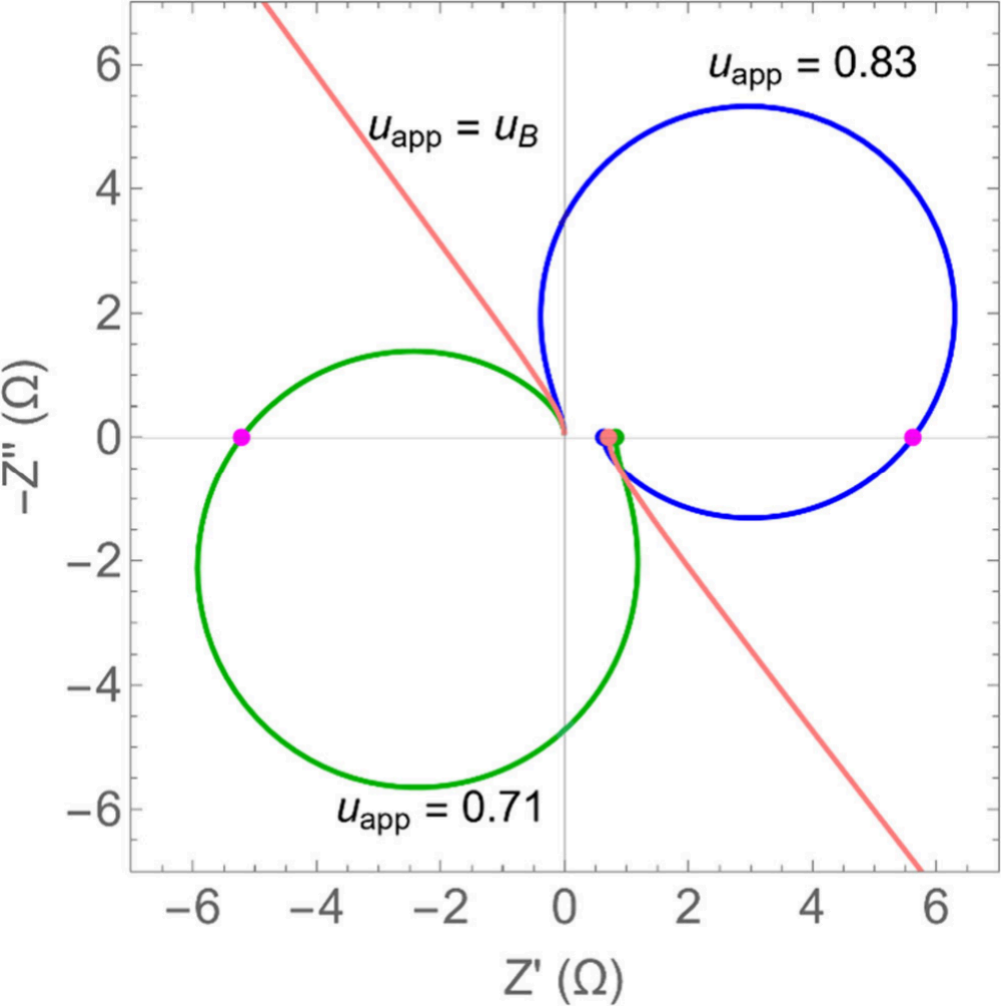
Impedance spectra of FitzHugh-Nagumo model at different applied voltages corresponding to Figure 12b, indicating the characteristic frequencies ω = 0 (same color as the spectrum) and the crossing of the horizontal axis (magenta). *u*_*B*_ = 0.775 is the Hopf bifurcation voltage.

**Figure 27:**
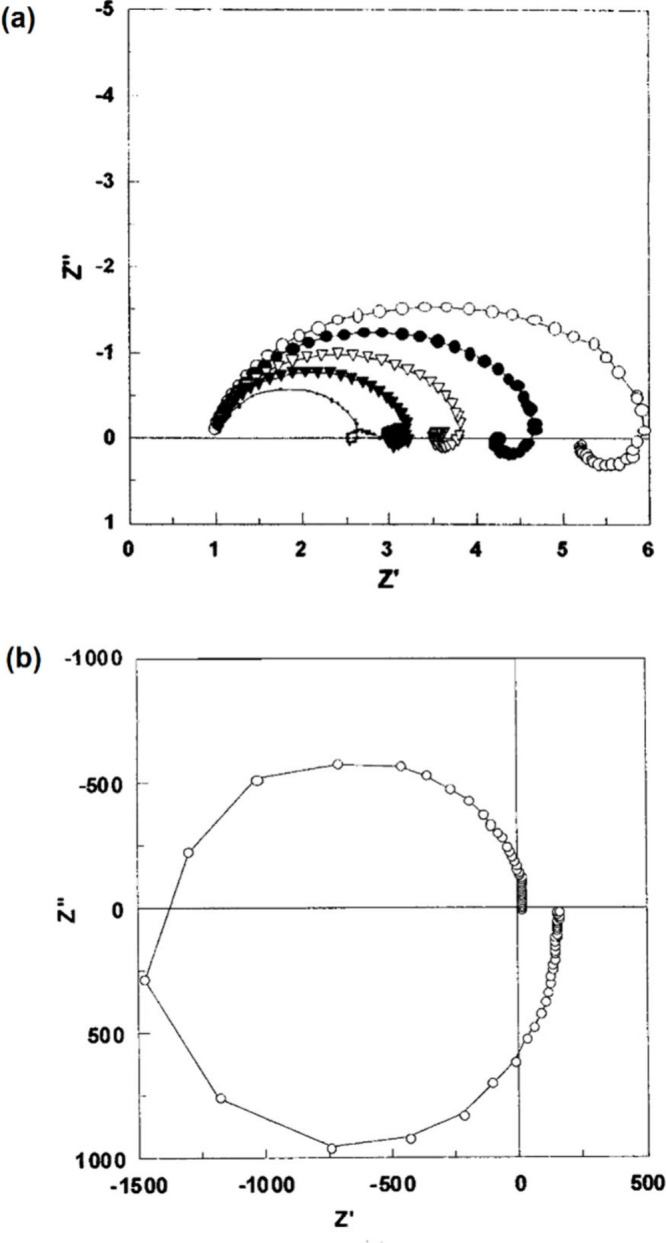
Impedance spectra of anodic dissolution of copper on 0.15 M CuSO_4_ and 5 M H_2_SO_4_ at 25 °C. (a) At low polarizations. (b) After the Hopf bifurcation. Reproduced with permission from ref 296. Copyright 2003 The Electrochemical Society.

In between the stable and limit cycle regions lies the Hopf bifurcation point, as we have seen. There, we have the condition *T*_*λ*_ = 0 and the frequency of the oscillations is ω_0*B*_ (Eq. 48). At this point the eigenvalues becomes purely imaginary and the impedance takes the form

85 $$Z\left(s\right)=\frac{\Delta u}{\Delta I_{0}}=\frac{1}{C_{0}\tau_{k}}\frac{\left(1+i\omega_{f}\tau_{k}\right)}{{\omega_{0B}}^{2}-{\omega_{f}}^{2}}$$

We can see that there is a pole where the impedance becomes infinite at the frequency *ω*_*f*_ = ω_0*B*_. Thus, the resonance condition that we stated in Eq. 1 is satisfied again. The singular spectrum is shown by a pink line in Figure 26. This singularity marks the qualitative change of the impedance from positive to negative intercept with the horizontal axis.

In summary, by the analysis of small signal impedance in the frequency domain we obtain that the neuron can be categorized both as a resonator and a self-sustained oscillator, depending on the point of operation.^172^ The resonant structure is provided by the combination of a constant capacitance such as the membrane capacitance, and the chemical inductor discussed in Sec. [Sec sec4.3] that is common to all the models listed in Table 1.

### 6.6 Nyquist Criterion for Oscillations

The Nyquist criterion for oscillators is a fundamental principle used to determine whether a system will spontaneously begin to generate periodic signals, i.e., whether it will oscillate.^297^ At its core, the criterion assesses the behavior of signals in a feedback loop. For a system to oscillate, two main conditions must be met: first, the signal that travels around the loop must return with the same phase, meaning there is constructive interference; and second, the amplitude of the returning signal must be at least as strong as the original – that is, the loop gain must be greater than or equal to one. These two conditions together indicate that any small perturbation in the system will be reinforced rather than dampened, leading to sustained oscillations.

When applied in the context of nonlinear dynamics and bifurcation theory – specifically for the Hopf bifurcation – the Nyquist criterion takes on an alternative, but related, interpretation using impedance. In terms of impedance, the Hopf bifurcation can be identified when the real part of the system’s total impedance becomes zero or negative at a specific frequency, as we have shown above. Thus, impedance spectra can be used to classify whether a system is near or undergoing a bifurcation. More specifically, the points in the impedance spectrum where the impedance becomes zero (its “zeros”) correspond to the eigenvalues of the system’s Jacobian matrix when the system is under potentiostatic control. These eigenvalues determine the stability of the system: when they cross into the right half of the complex plane, oscillations begin. Consequently, the Nyquist criterion for the Hopf bifurcation^298^ can be translated into impedance conditions.^45^ Oscillation can occur if the real part of the total impedance becomes zero or negative at some frequency. Therefore, the impedance spectra can be classified to indicate bifurcations. As mentioned, zeros of the impedance correspond to the eigenvalues of the Jacobian under potentiostatic control. Poles of the impedance correspond to the eigenvalues under galvanostatic control.^45,288,289,299^ Consequently, an effective way to analyze the potential for self-oscillation and classify the bifurcations is by examining the impedance function of the system and its corresponding poles and zeros in the complex frequency domain.^27,290–292^ In this way, the Nyquist criterion, impedance analysis, and bifurcation theory come together to provide powerful tools for predicting the onset of oscillatory behavior in dynamic systems.

## 7 Feedback-Based Oscillators

Feedback-based oscillators are self-sustained dynamical systems in which oscillatory behavior is determined by feedback-induced instabilities and circuit parameters, rather than by the DC operating points or intrinsic NDR. Although the trivial equilibrium state at zero voltage and current does not supply energy, it can become unstable for appropriate parameter values, such that small perturbations (such as thermal noise in experiments or a numerical kick-start in simulations) are sufficient to initiate oscillations. Once triggered, the oscillatory dynamics are sustained by the feedback loop and can be analyzed within the same nonlinear dynamical framework developed in the preceding sections. This feedback loop, under appropriate conditions, enables the continuous regeneration and maintenance of the oscillatory behavior.^300,301^

In conventional electronic implementations, such oscillators are typically composed of two functionally distinct components. The first is the frequency selector network, β, which governs key properties of the oscillation like the frequency, as shown in Figure 28. The second is an active amplification element, gain stage A, which provides the necessary gain to sustain the oscillations. This active element is often realized through an Op-Amp or an equivalent configuration of transistors, designed to ensure the circuit meets the necessary gain and phase criteria for oscillations.^83^

**Figure 28:**
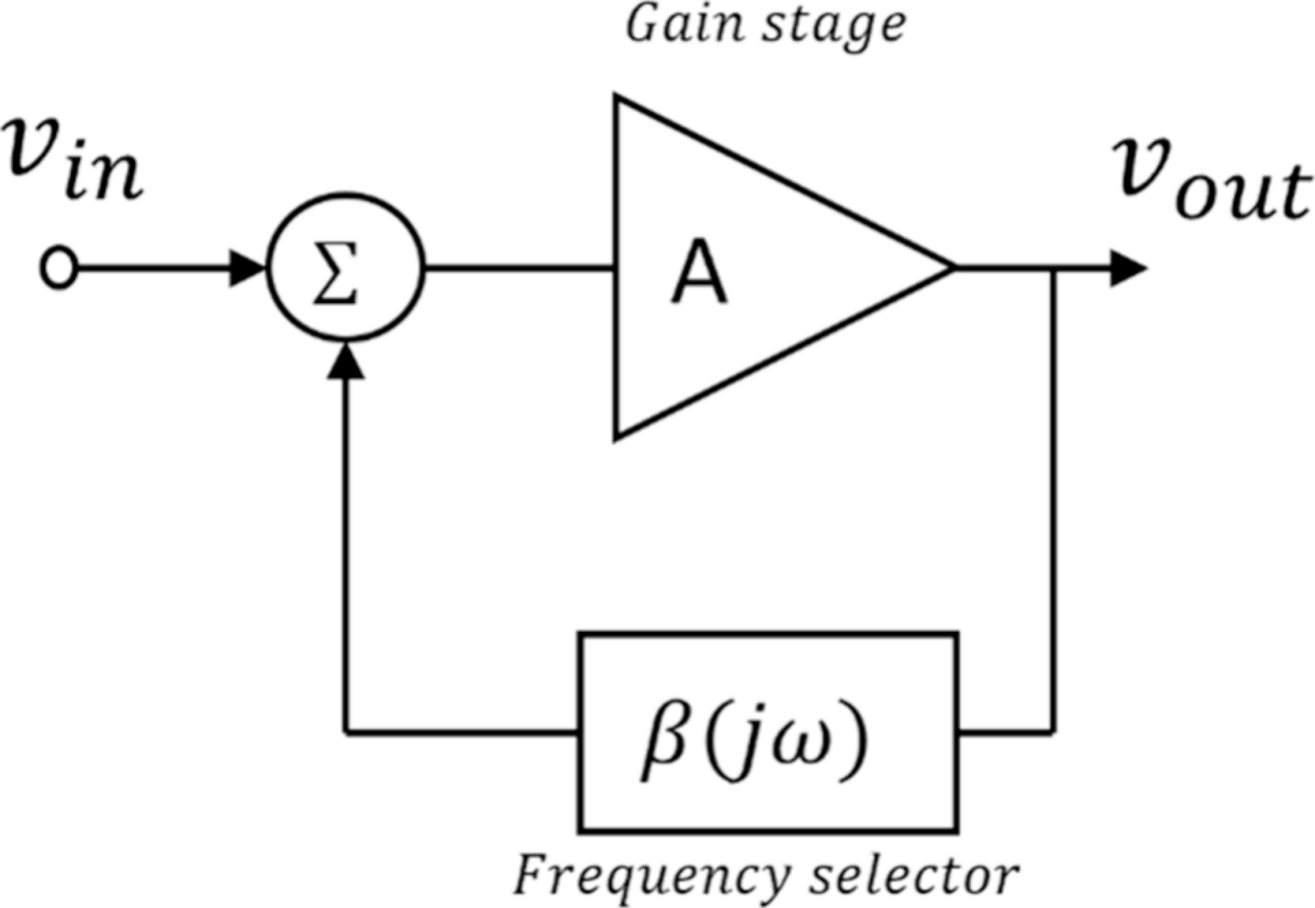
Block diagram of the system used in feedback-based oscillators, highlighting the two fundamental components: the gain stage and the frequency selector.

These conditions for the onset and stability of oscillations in feedback-based oscillators are determined by well-established theoretical conditions, most notably the Barkhausen and Nyquist or Routh-Hurwitz criteria.^297,302^ These formalisms have already been introduced previously and define the necessary requirements that a system must fulfill in order to sustain periodic behavior. They state that for a circuit to oscillate, the loop gain must be equal to or greater than unity, and the total phase shift around the loop must be an integer multiple of 2π. This ensures that the signal constructively reinforces itself after each cycle, allowing oscillations to persist without attenuation. In practical terms, these criteria guide the design and tuning of the oscillator’s components, particularly the feedback network and the active amplification stage, to guarantee the precise conditions under which self-sustained oscillations emerge and remain stable over time.

The amplification element within a feedback-based oscillator can also be configured to implement either positive or negative feedback, depending on the phase relationship between the output and the input signals. In the case of positive feedback, the output signal is in phase with the input and of sufficient amplitude, effectively reinforcing the input and promoting the growth or maintenance of the oscillation. This in-phase and amplified reinforcement forms a constructive feedback loop, precisely the condition described by the Barkhausen and Nyquist criterion previously discussed, and is essential for sustaining an undamped oscillatory behavior. Conversely, in a negative feedback configuration, the output signal is out of phase with the input, typically by 180 deg, resulting in a signal that opposes the input and thus attenuates the system’s response. While negative feedback is commonly employed in amplifiers to enhance stability and linearity, it is generally avoided in oscillator design when the goal is to initiate and sustain oscillations.^303–305^

Feedback oscillators can generate both sinusoidal and nonsinusoidal waveforms, and their role is foundational across a range of disciplines, including electronics, signal processing, control systems, communications, and even biological systems where oscillatory behavior is intrinsic to function.^306,307^

Understanding the principles that govern the operation of feedback-based oscillators is not only essential in classical electronics but also increasingly relevant for the development of novel self-sustained oscillatory devices. In particular, leveraging the feedback mechanisms inherent in these systems provides a powerful strategy for inducing oscillations in emerging materials and architectures.^308,309^

With this foundational understanding in place, we now proceed to examine the different categories of feedback-based oscillators, distinguishing between those that generate sinusoidal and nonsinusoidal signals. In the case of sinusoidal oscillators, the oscillatory behavior can be derived directly from the linear differential equations governing the circuit dynamics. By analyzing the system in this framework (following the previous discussion in Secs. [Sec sec4.1] and 4.5), we will revisit and rigorously reinterpret the classical Barkhausen and Nyquist criteria as applied in these configurations.^310^ This analytical approach allows for a clear identification of the frequency and stability conditions associated with purely harmonic oscillations and serves as a bridge toward more advanced modeling. In parallel, this framework opens the door to extending the analysis into nonlinear regimes, where real-world circuit nonidealities and intrinsic nonlinearities must be taken into account.^311,312^

On the other hand, nonsinusoidal oscillators, such as relaxation oscillators^216^ or multivibrators,^313^ will be addressed only briefly. Although these systems are capable of producing sharp transitions and nonharmonic waveforms, their inherent complexity and lack of controllable frequency response reduce their controlled applicability where smooth and tunable oscillatory dynamics are generally preferred.

### 7.1 Sinusoidal Feedback-Based Oscillators

Sinusoidal feedback-based oscillators are a class of systems which, under appropriate conditions, exhibit a sustained oscillatory response characterized by a purely sinusoidal waveform. The frequency of these oscillations is determined by the frequency selector network embedded within the circuit architecture. Depending on the specific structure and components of this network, various types of feedback-based sinusoidal oscillators have been developed, each with distinct frequency characteristics and stability properties.^314–316^ In this section, we focus on the most widely used and technologically relevant configurations.^317^

A common and insightful feature of these circuits is their behavior as a function of the loop gain. By varying the gain of the amplification stage, the system can transition through different dynamical regimes: from a stable fixed point (no oscillation), to a regime of steady-state sinusoidal oscillations, and, depending on the nonlinear characteristics of the system, eventually to unstable or growing oscillations in a limit cycle. Consequently the gain of the amplification stage will be the bifurcation parameter. The system exhibits a subcritical-like Hopf bifurcation: oscillations appear abruptly and are unstable just beyond the threshold.^318^

Understanding this bifurcation framework is fundamental, as it captures the intrinsic nature of the transition to oscillatory behavior in real-world circuits. Here, we will first derive the conditions for oscillation and their stability for an idealized system with linear gain, and then contrast them with the behavior introduced by nonlinear gain saturation.

Among all sinusoidal feedback-based oscillator topologies, the Wien-Bridge oscillator stands out as the most basic yet conceptually illustrative example.^317^ It consists of an Op-Amp configured with two resistors that define its gain, and a frequency selector Wien-Bridge network. The Wien-Bridge network itself is formed by two RC branches: one configured as a series RC and the other as a parallel RC. These are connected in series and placed between the output and the input terminals of the Op-Amp to establish the desired frequency-dependent feedback.^319^

In Figure 29, we show the two most basic configurations of the Wien-Bridge oscillator. We now analyze the differential equations that describe the behavior of these systems. As done in Sec. [Sec sec4], we will use the characteristic Jacobian matrix to extract the conditions for oscillation, determine their stability, and calculate the oscillation frequency.

**Figure 29:**
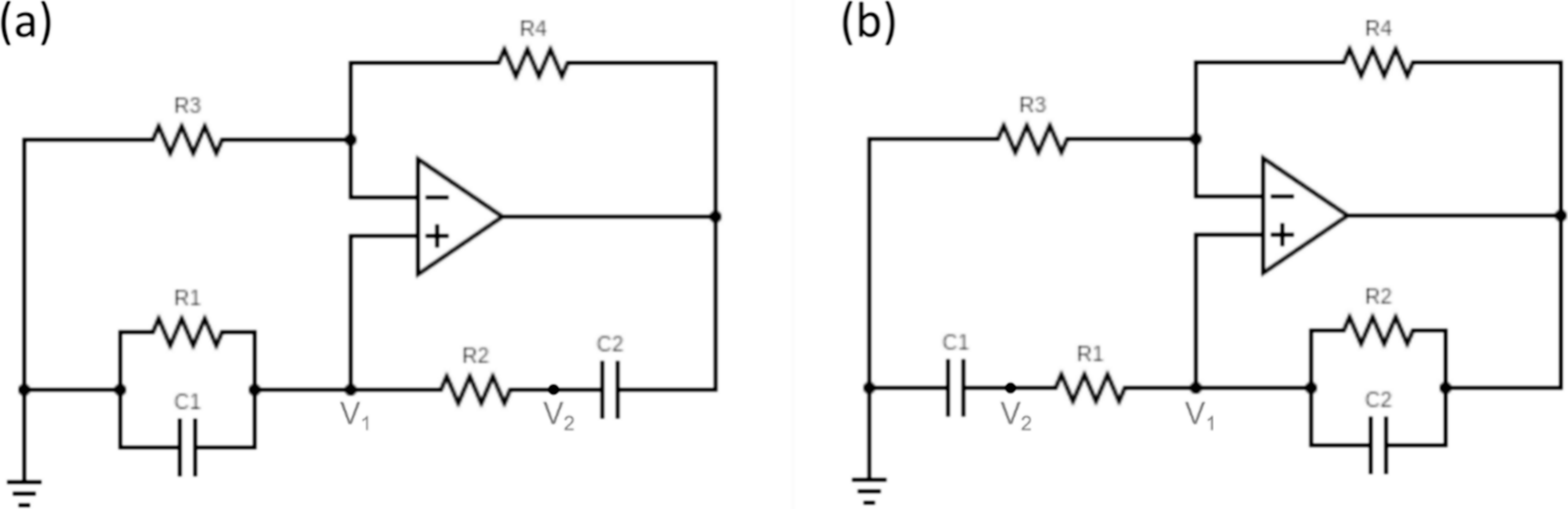
Representation of the circuits of the two different configurations for the Wien-Bridge oscillator. *V*_1_ and *V*_2_ are the voltage variables determining the future state of both systems.

We will also point out the key difference between both circuits, which has been the subject of discussion in some studies, particularly regarding the interpretation of the Barkhausen and Nyquist criteria.

The differential equations governing the system shown in Figure 29a, derived using basic circuit laws such as Kirchhoff’s voltage and current laws, and assuming an ideal op-amp, are given by

86 $$F=\frac{dV_{1}}{dt}=\frac{1}{C_{1}}\left[\frac{1}{R_{2}}V_{2}-\left(\frac{1}{R_{1}}+\frac{1}{R_{2}}\right)V_{1}\right]$$

87 $$G=\frac{dV_{2}}{dt}=\left(\frac{A}{C_{1}R_{2}}-\frac{1}{C_{2}R_{2}}\right)V_{2}+\left(\frac{1}{C_{2}R_{2}}-\frac{A}{C_{1}R_{1}}-\frac{A}{C_{1}R_{2}}\right)V_{1}$$

where *V*_1_ denotes the voltage at the non-inverting input of the operational amplifier, *V*_2_ corresponds to the voltage at the node between *R*_2_ and *C*_2_, and the amplifier gain, denoted by *A*, is determined by the resistor network composed of *R*_3_ and *R*_4_, and is given by the well-known expression

88 $$A=1+\frac{R_{4}}{R_{3}}$$

With these definitions in place, we can now construct the Jacobian matrix of the system by evaluating the partial derivatives of the governing equations with respect to the state variables,

89 $$F_{V_{1}}=\frac{dF}{dV_{1}}=-\frac{1}{C_{1}}\left(\frac{1}{R_{1}}+\frac{1}{R_{2}}\right)$$

90 $$F_{V_{2}}=\frac{dF}{dV_{2}}=\frac{1}{C_{1}R_{2}}$$

91 $$G_{V_{1}}=\frac{dG}{dV_{1}}=\frac{1}{C_{2}R_{2}}-\frac{A}{C_{1}R_{1}}-\frac{A}{C_{1}R_{2}}$$

92 $$G_{V_{2}}=\frac{dG}{dV_{2}}=\frac{A}{C_{1}R_{2}}-\frac{1}{C_{2}R_{2}}$$

the trace *T*_*λ*_ and determinant Δ_λ_ obtained from this Jacobian are given by

93 $$T_{\lambda }=F_{V_{1}}+G_{V_{2}}=\frac{A}{C_{1}R_{2}}-\frac{1}{C_{1}R_{2}}-\frac{1}{C_{1}R_{1}}-\frac{1}{C_{2}R_{2}}$$

94 $$\Delta_{\lambda }=F_{V_{1}}G_{V_{2}}-F_{V_{2}}G_{V_{1}}=\frac{1}{R_{1}C_{1}R_{2}C_{2}}$$

We observe that the Jacobian matrix always exhibits a positive determinant. The trace, however, can take either positive or negative values depending on the gain A and the values of the circuit elements, all of which are assumed to be positive.

Assuming the most general symmetric case, where *C*_1_ = *C*_2_ = *C* and *R*_1_ = *R*_2_ = *R*, the trace becomes zero when *A* = 3. This leads directly to the condition

95 $$A=3\to R_{4}=2R_{3}$$

and, when this condition is satisfied, the system reaches the bifurcation point, and sustained sinusoidal oscillations emerge with a well-defined characteristic frequency

96 $$\omega_{0}={\left(\Delta_{\lambda }\right)}^{1/2}=\frac{1}{RC}$$

If the trace takes negative values, i.e., when *R*_4_ < 2R_3_, the system remains in a stable regime. In this case, small initial oscillations may appear but will decay over time, and the system will eventually settle to its equilibrium point. On the other hand, if the trace is positive, i.e., R_4_ > 2*R*_3_, the system enters an unstable regime. For an ideal Op-Amp, this would lead to oscillations with amplitudes that grow indefinitely over time. However, in a more realistic scenario, where the Op-Amp has saturation limits on its output voltage, the system evolves into a limit cycle regime. In this case, the amplitude of the sustained oscillations is constrained by the saturation levels. All these different oscillatory behaviors are shown in Figure 30a.

**Figure 30:**
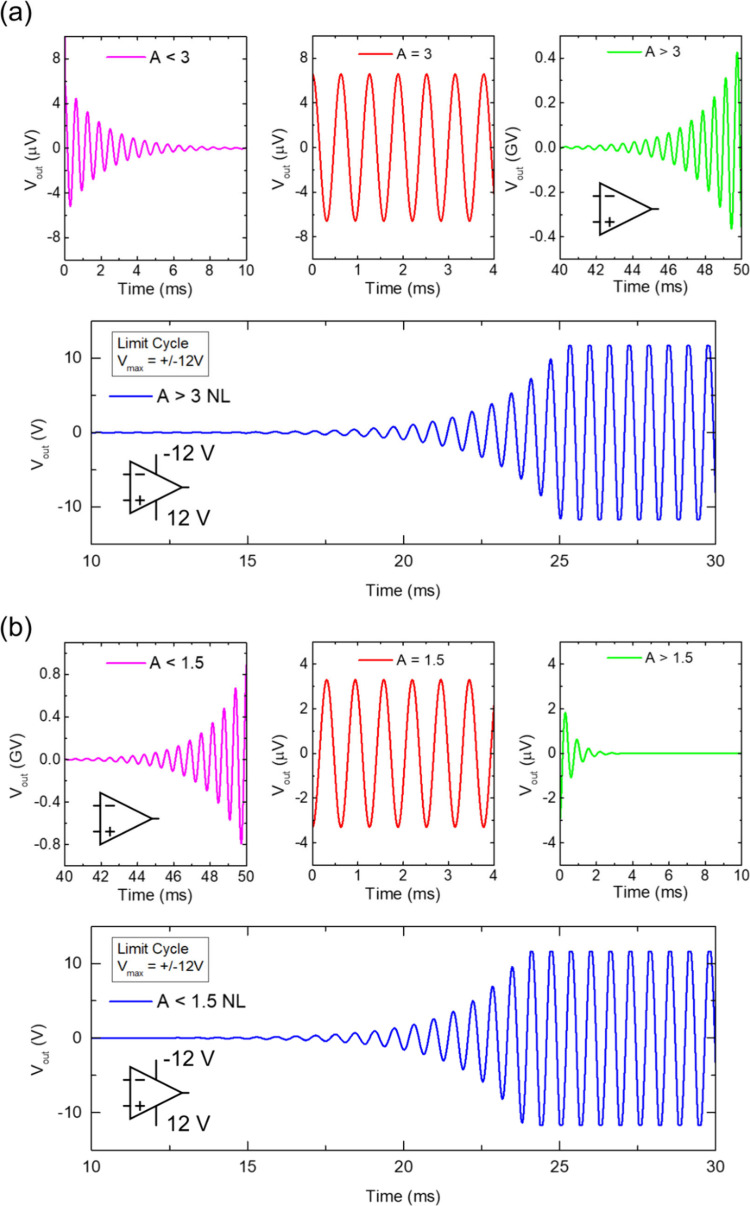
(a) Simulated dynamical responses of the Wien-Bridge oscillator for the circuit of Figure 29a for different gain values *A*, adjusted via the resistive network at the op-amp input. (Top) Representative voltage traces corresponding to subcritical gain (*A* < *A*_*biff*_), critical gain (*A* = *A*_*biff*_) and supercritical gain (*A* > *A*_*biff*_) simulated by assuming an ideal op-amp model (unbounded output). (Bottom) Voltage trace for the last case but considering imposed saturation limits (*V*_*sat*_ = ± 12 *V*), illustrating the emergence of a limit cycle over the bifurcation constrained by amplifier nonlinearities. (b) Same as (a) but for the alternative configuration of Figure 29b.

For the configuration shown in Figure 29b, following the same procedure, the trace of the Jacobian is given by

97 $$T_{\lambda }=\frac{1}{\left(A-1\right)R_{1}C_{2}}-\frac{1}{R_{1}C_{1}}-\frac{1}{R_{2}C_{2}}$$

Assuming again the classical case where *C*_1_ = *C*_2_ = *C* and *R*_1_ = *R*_2_ = *R*, the bifurcation point is reached when

98 $$A=\frac{3}{2}\to R_{4}=\frac{R_{3}}{2}$$

In this case, the oscillation conditions obtained for the configuration of Figure 29a are reversed. Specifically, the trace becomes negative when *R*_4_ > *R*_3_/2, corresponding to a stable equilibrium state. Conversely, when *R*_4_ < *R*_3_/2, the trace is positive, and the system exhibits the same oscillatory behavior described earlier, but under opposite conditions. This is shown in Figure 30b.

All the results obtained here are in full agreement with previous studies on the stability and oscillatory behavior of this type of oscillator, whether derived from a differential equation analysis or from classical criteria such as those of Barkhausen and Nyquist.^310,320,321^

After completing the dynamic analysis and establishing the oscillation and stability conditions for one of the most fundamental oscillator circuits, we now briefly introduce other feedback-based sinusoidal oscillator configurations of interest and highlight their key differences.

As previously explained, the essential components required for a feedback-based oscillator are a frequency selector network and an amplification stage that provides the necessary feedback to this network. By modifying the structure of the frequency selector part, a wide variety of oscillator topologies can be implemented. These configurations can be further optimized by adapting the amplification stage for example, by using transistors for automatic gain control, different types of amplifiers and buffers^322^ or simplified analog circuits,^323^ in order to better control the conditions for oscillation and improve overall stability. Figure 31 shows the basic structures of several representative oscillator circuits.

**Figure 31:**
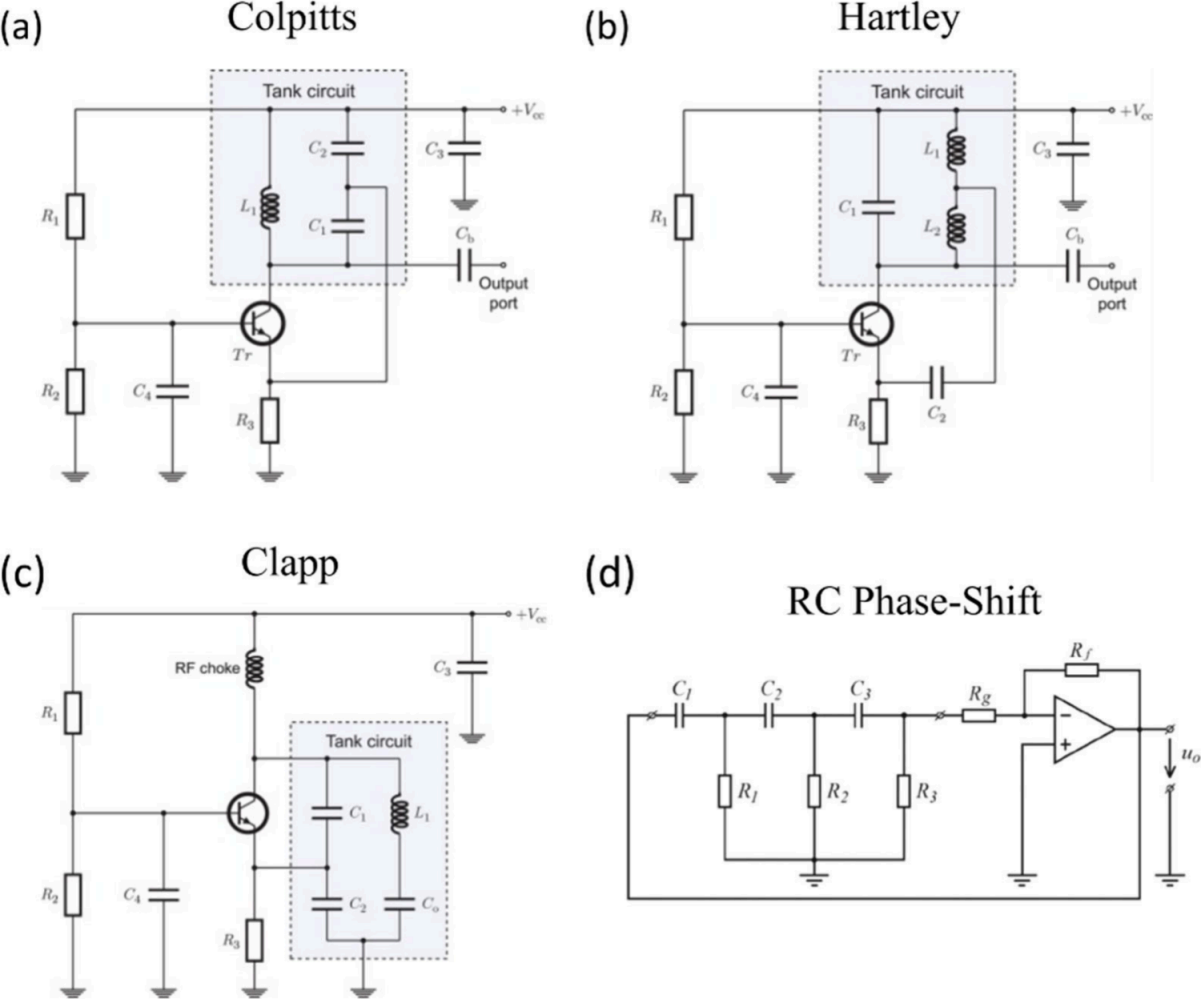
Basic schematic representations of four fundamental sinusoidal feedback-based oscillators. (a) Colpitts oscillator, (b) Hartley oscillator and (c) Clapp oscillator. Reproduced with permission from ref 324. Copyright 2016 Elsevier. (d) RC Phase-Shift oscillator. Reproduced with permission from ref 311. Copyright 2023 Wiley.

One of the most basic Colpitts type oscillators is shown in Figure 31a. This circuit typically employs a bipolar junction transistor (BJT) as the active amplification element, along with resistors and capacitors to ensure the required gain and phase shift. The frequency selector network is based on a classic LC tank circuit, as previously discussed in Figure 10a, but with the inclusion of an additional capacitor connected between two terminals of the amplifying element (in this case, the BJT).

The oscillation frequency of the Colpitts oscillator is determined by the inductance *L* and the two capacitors *C*_1_ and *C*_2_ building the tank circuit, and is given by

99 $$\omega_{0}=\frac{1}{\sqrt{L\frac{C_{1}C_{2}}{C_{1}+C_{2}}}}$$

This oscillator is generally used for higher frequency applications than the Wien-Bridge oscillator, typically operating in the radio frequency (RF) while the Wien-Bride operates in the audio frequency range.

Moving now on to Figure 31b, we consider the Hartley oscillator, which is structurally similar to the Colpitts oscillator. However, instead of introducing the feedback through a capacitor connected in series with the original tank circuit capacitor, the Hartley configuration achieves feedback by adding an additional inductor in series with the original tank circuit inductor. These two inductors are often implemented as a single coil winding with a center tap, simplifying the physical construction of the circuit.

The oscillation frequency is determined in a manner analogous to the Colpitts oscillator and is given by

100 $$\omega_{0}=\frac{1}{\sqrt{\left(L_{1}+L_{2}\right)C}}$$

where the series capacitors of the Colpitts circuit are now replaced by series inductors. The Hartley oscillator is typically used in the same range of applications as the Colpitts, particularly in RF circuits. One practical advantage of this configuration is that frequency tuning can be achieved using a single capacitor, which simplifies control in certain situations.^325^

Figure 31c shows the Clapp oscillator, which follows the same general design approach as the previously described Colpitts and Hartley oscillators. Similar to the Colpitts configuration, a capacitor is placed in series with the original tank circuit capacitors. However, the Clapp design also introduces an additional capacitor in series with the inductor of the tank circuit. As a result, the new resonant network consists of two capacitors in series, effectively acting in parallel with a series combination of the inductor and the third capacitor.

The oscillation frequency for this circuit is given by

101 $$\omega_{0}=\frac{1}{\sqrt{\frac{1}{L}\left(\frac{1}{C_{1}}+\frac{1}{C_{2}}+\frac{1}{C_{0}}\right)}}$$

where *C*_0_ is the additional capacitor connected in series with the inductor *L*. This configuration offers improved frequency stability under gain variations, making the Clapp architecture particularly suitable for implementation in voltage-controlled oscillator (VCO) circuits.^326,327^

For a more detailed analysis of the oscillation and stability conditions of these three oscillator types, one can refer to classical circuit theory textbooks, where the oscillation frequencies are derived explicitly. From an impedance-based analysis of the tank circuit, the required gain conditions for oscillation can also be determined as a function of the amplifier characteristics.^324,328^

Finally, in Figure 31d, we return to a design more closely related to the Wien-Bridge oscillator, as it also relies solely on resistors, capacitors and an Op-Amp. This oscillator consists of an inverting Op-Amp connected to a cascade of typically three RC stages, although many variations of this circuit exist.^311^ The configuration shown represents the most basic version.

In this circuit, the oscillation frequency is determined by the values of the resistors and capacitors in the RC network. Assuming the symmetric case where *R*_1_ = *R*_2_ = *R*_3_ = *R* and *C*_1_ = *C*_2_ = *C*_3_ = *C*, the oscillation frequency is

102 $$\omega_{0}=\frac{1}{RC\sqrt{6}}$$

The analysis of the stability and oscillation conditions for different RC phase-shift oscillator configurations follows a similar approach to that used previously for the Wien-Bridge oscillator.^311^ The operating frequency range is also comparable, typically in the audio frequency domain.

Although not shown in Figure 31, it is worth noting the existence of additional sinusoidal oscillator topologies that are particularly relevant in multisignal generation and multisignal analysis applications. Those are the so-called quadrature oscillators,^311^ which are modified versions of the RC phase-shift oscillators designed to produce two output signals with a phase difference of 90 deg, generating cosine- and sine-like waveforms simultaneously. Within this class of oscillators, there are also variants specifically designed to generate multiple signals with precise phase differences. One notable example is the Bubba oscillator,^317^ which produces four output signals, each phase-shifted by 45 deg relative to the next. This configuration is particularly useful in applications requiring quadrature and polyphase signal generation.

### 7.2 Nonsinusoidal Feedback-Based Oscillators

We now briefly turn to the other category of feedback-based oscillators, in which the output is no longer sinusoidal but rather consists of nonharmonic waveforms, typically triangular, square or sawtooth-shaped. These are known as relaxation oscillators, already introduced in Sec. [Sec sec4.6]. These circuits are historically among the first electronic oscillators developed and are widely used in basic applications due to their simplicity and ease of implementation.^329^

The simplest electronic relaxation oscillator is based on an Op-Amp, as shown in Figure 32a. Its structure and operation are straightforward and commonly taught in undergraduate electronics courses. The circuit operates through mutual feedback, with the Op-Amp acting alternately as a comparator (Schmitt trigger), generating a square wave, and as integrator, producing a triangular wave. This basic structure is widely used in simple signal generators, waveform synthesis and low-complexity VCOs.^216^

**Figure 32:**
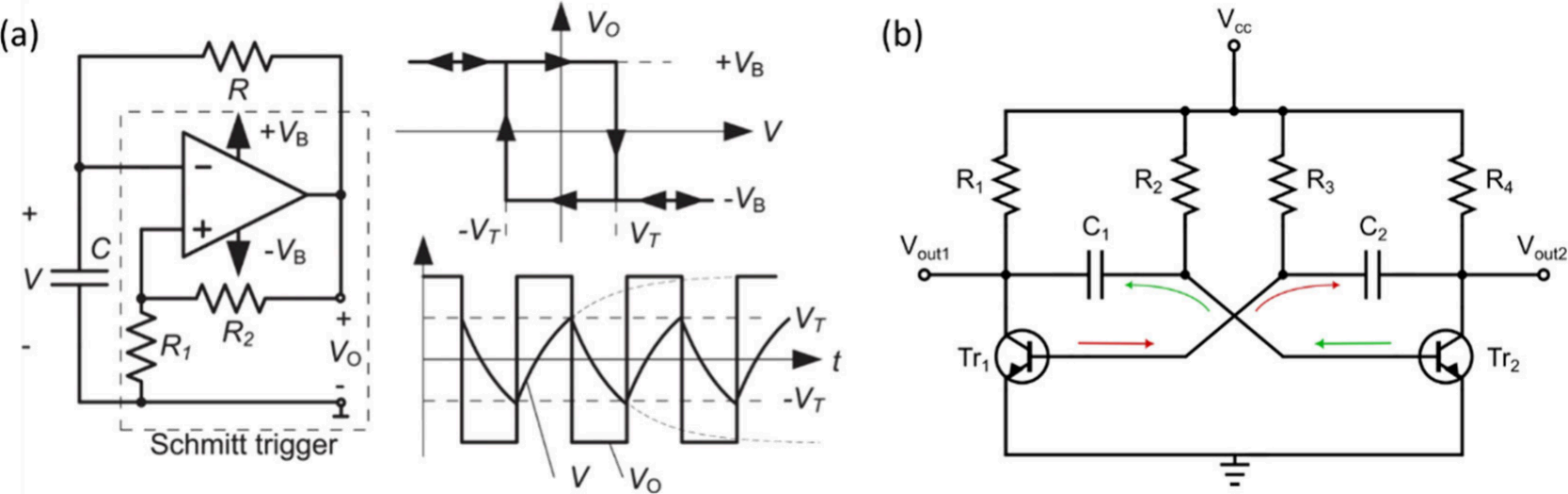
Basic topologies and waveforms of representative nonsinusoidal feedback-based oscillators. (a) Op-Amp relaxation oscillator. Which produces a triangular waveform at the integrator output and a corresponding square wave at the comparator output. Reproduced from ref 216. Available under a CC-BY 4.0 license. Copyright 2023 The Author(s). Published by the Institute of Electrical and Electronics Engineers (IEEE). (b) Astable multivibrator. This generates two antiphase square wave outputs at nodes *V*_*out*__1_ and *V*_*out*__2_. The output levels alternate between the supply voltage *V*_*cc*_ and ground.

Building on the principles of this elementary relaxation oscillator, more complex architectures have been developed to meet higher-level application needs.^309,330–332^ One such evolution is the astable multivibrator, which in its simplest form consists of two cross-coupled transistors and two capacitors. The circuit alternates between ON and OFF states of both transistors, driven by the charging and discharging dynamics of the cross-coupled capacitors, thereby producing square waves, the architecture is shown in Figure 32b. This basic multivibrator is still used in common devices such as digital clocks, blinking lights and simple logic circuits.^333^

More advanced implementations lead to the well-known 555 timer-based oscillator, which incorporates, to the simpler multivibrator, additional components like comparators, discharge transistors or integrated circuits like Flip-Flop RS. These allow for more refined control over the waveform and timing, enabling its use in a wider range of applications, including alarm systems, motor control, and more complex timing functions.

### 7.3 Hybrid Oscillators: Feedback Loops in Emerging Devices

Now that we have established a solid understanding of classical feedback-based oscillators, we are in a position to explore new and emerging electronic architectures that integrate feedback mechanisms with devices exhibiting novel chemical-physical properties. These hybrid systems are attracting increasing attention across various domains, as they enable the realization of application-specific oscillators that surpass the limitations of conventional designs.^334^ Examples include electrochemical devices for mimicking biological neurons,^72,335–337^ mechanical resonators for sound detection and frequency adaptation,^308,338^ and magnetic systems for threshold-based magnetic sensing.^339^

All of these systems share a common architectural principle: the integration of functional materials or components, engineered for a specific response, with an amplification stage that introduces feedback to induce or control oscillatory behavior under certain conditions. While various device types are currently being investigated as emerging resonant elements, in this work we focus on the biologically relevant case: the use of OECTs as the core element in biologically inspired spiking oscillators. This strategy represents a key step toward replicating neuronal functionality in perception-responsive electronic systems,^340,341^ operating through gate-controlled electrochemical doping and dedoping of organic mixed ionic-electronic conductors (OMIECs).^342–344^

A fundamental requirement for achieving spiking behavior in these systems is the reduction of carrier mobility at high ionic concentrations, an intrinsic property of many OMIEC materials. Under this condition, the system exhibits a region of negative transconductance, either through antiambipolar characteristics, achieved by partially stacked p-n heterojunctions in the transistor channel, or via the saturation of available electronic states in the density of states. These devices, already discussed in Figure 18 above, are particularly suitable for emulating the signal dynamics of natural neurons, using similar physical-chemical principles to generate biocompatible oscillations.^75,78^ Previous implementations of OECT-based spiking neuron elements have relied on combinations of multiple transistors and feedback-driven amplification stages to reproduce essential features of neural activity.^72^

These hybrid oscillator circuits combining OECTs and electronic feedback stages have been investigated as prototypical examples of emergent neuromorphic architectures, where feedback and material nonlinearities coexist to generate tunable self-sustained oscillations. In our recent work,^345^ based on experimental results of Harikesh et al.,^72^ we developed a comprehensive modeling framework for these amplifier-assisted organic electrochemical neuron circuits. By formulating the hybrid system as a set of coupled nonlinear differential equations describing the membrane potential and internal ionic variables, we identified the onset of self-sustained oscillations through a Hopf bifurcation and analyzed their evolution via nullclines and phase-space trajectories. This dynamic systems perspective complements classical circuit-theoretic approaches, revealing how feedback gain, transconductance nonlinearity, and ionic relaxation times jointly determine the oscillation frequency and stability. The methodology provides a quantitative route to tune oscillatory behavior by modifying circuit parameters or material properties, and establishes a general framework for designing compact, biologically inspired hybrid oscillators based on OECTs.

A general overview of the study presented in that work is illustrated in Figure 33.^345^ The figure shows the equivalent circuit of the model employed (Figure 33a), which consists of three main branches. The first branch contains the capacitor associated with the fast variable of the oscillation. The second branch corresponds to an activation-like variable that can be modeled either through an activation function or, equivalently, by a resistor and an inductor; biologically, this branch represents the K^+^ channel and is replicated using an OECT that conducts K^+^ ions. Finally, the third branch includes both the element exhibiting NDR (negative transconductance) and the feedback component that closes the loop of the system, the inverting Op-Amp. Biologically, this corresponds to the Na^+^ channel, with its activation and deactivation dynamics, while in the artificial circuit it can be implemented by an OECT based on a MIEC operating in a negative-transconductance regime combined with an inverting amplifier.

**Figure 33:**
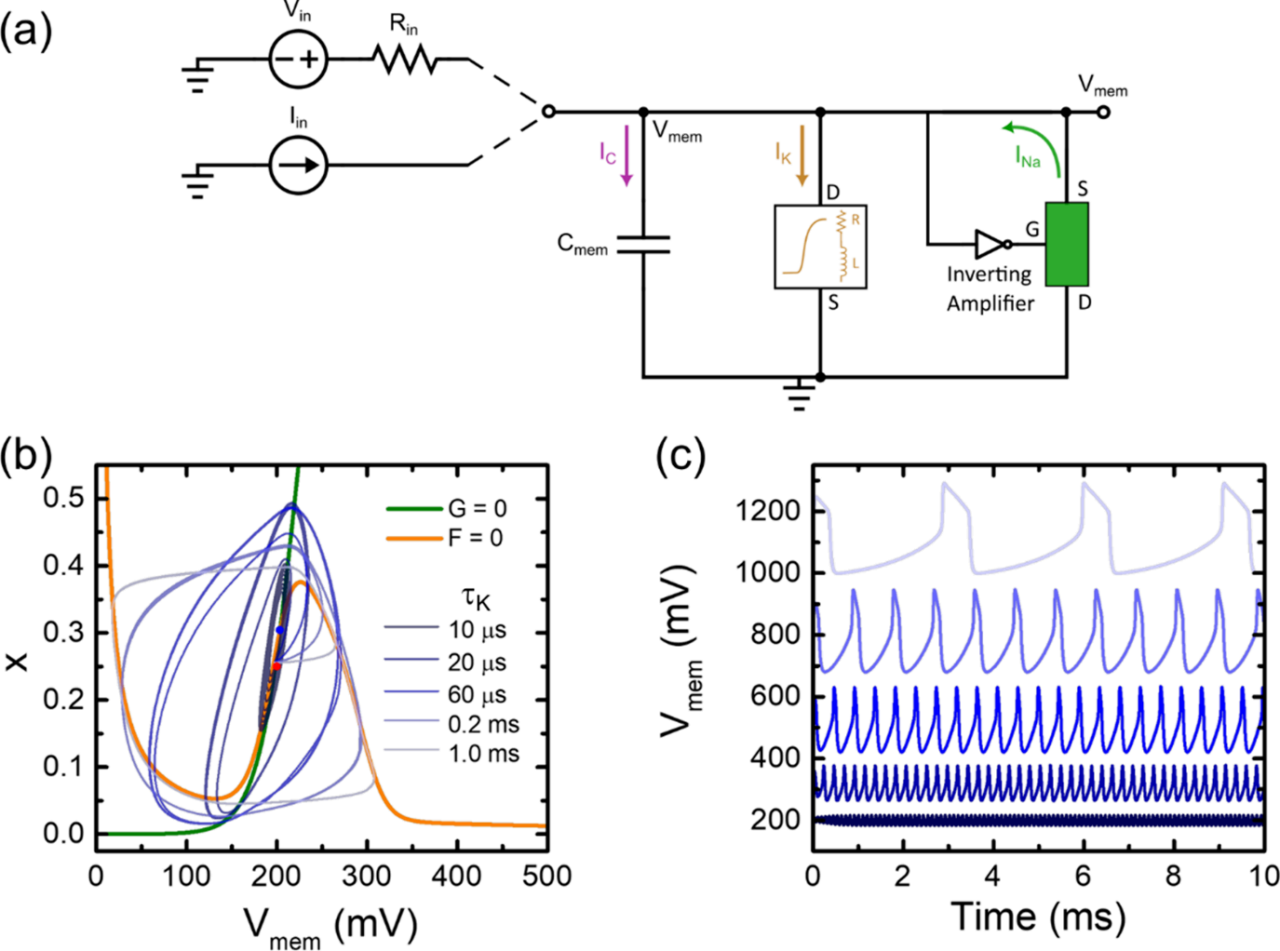
(a) Schematic circuit diagram of the hybrid oscillator model combining a feedback loop with a NDR device, as presented in.^345^ The green block represents the transistor exhibiting negative-transconductance, connected to an inverting amplifier, while the middle branch corresponds to the activation-type transistor that emulates the K^+^ channel. (b) Phase-space representation of the system, showing both nullclines *F* = 0 and *G* = 0 and the oscillatory trajectories for different relaxation times of the potassium channel activation variable. The red dot indicates the initial values of the variables, whereas the blue dot marks the unstable equilibrium point of the system (the cross-point of the nullclines). (c) Time domain oscillations of the circuit’s membrane voltage variable *V*_*mem*_. The waveforms are vertically offset for clarity, from bottom to top: 0 mV, 140 mV, 360 mV, 650 mV, and 980 mV. The differential equations used in the model are identical to those reported in^345^ and the parameter values employed are as follows: *C_m_* = 10 *nF*; *g_K_* = 0.80 *mS*; *V_x_* = 0.22 *V*; *V_mx_* = 0.02 *V*; *R_f_* = 600 *k*Ω; *R_in_* = 200 *k*Ω; *V*_0_ = 0.35 *V*; *g_Na_* = 0.10 *mS*; *V_a_* = 0.50 *V*; *V_m_a__* = 0.04 *V*; *V_d_* = 0.80 *V*; *V_m_d__* = 0.04 *V*; *I_in_* = 5 μ*A*. Reproduced from Rivera-Sierra, G.; Fenollosa, R.; Bisquert, J. *ACS Applied Electronic Materials* **2025**, *7*, 10620–10630. Copyright 2025 American Chemical Society.

The presence of this Op-Amp is essential in this case to accurately reproduce the neuronal functionality while maintaining biocompatibility. As discussed previously, similar oscillations can also be reproduced without explicit feedback components when a NDR mechanism is inherently present. In Figure 33(b,c), the system phase space and oscillations are represented with parameters for which the equilibrium point is unstable, and the relaxation time of the K^+^ activation branch (the slow variable) is varied. This allows continuous tuning from small-amplitude and high-frequency oscillations around the limit cycle (*τ*_*k*_ < ) to relaxation-type, bigger amplitude and smaller frequency oscillations (τ_K_ > ), as observed in theoretical systems such as the FHN model (see Figure 12).

This oscillator circuit has also been recently applied in other contexts, such as in simple logic circuits that emulate neuronal operations through oscillatory behavior modulated by the mimic of voltage-gated dendritic calcium dynamics.^346^ Furthermore, the negative-transconductance exhibited by the OECT has been successfully exploited to develop single-transistor neuron architectures.^335^ These advances highlight the critical importance of fully understanding, analyzing, and controlling the various types of responses that these emerging circuits can exhibit.

Altogether, this comprehensive analysis demonstrates that the emergence of self-sustained oscillations in any system must be grounded in the bifurcation theory analysis outlined in this review. Regardless of the specific implementation–whether based on feedback circuits, device-level dynamics, or hybrid architectures–such systems can be fully understood, controlled, and engineered by analyzing their phase space and nullcline structure. This enables precise control over, why, when and how oscillations appear, what device parameters must be modified to target a given function, and how degradation, noise, or amplifier variations might affect performance. Ultimately, this approach provides a robust foundation for the design of next-generation neuromorphic and bioelectronic systems.

## 8 Conclusion

The growing demand for adaptive, energy-efficient computing has intensified research in neuromorphic systems that emulate the brain’s architecture and functional principles. Unlike conventional von Neumann machines (powerful yet energy-intensive) neuromorphic and related unconventional systems aim to achieve high computational performance with ultralow power consumption. They are particularly suited for robotics, wearable electronics, distributed sensors, and other contexts where energy and footprint are constrained. By mimicking evolved biological strategies, biomimetic devices can achieve similar information-processing capabilities with remarkable efficiency.

At the core of both biological and artificial neuromorphic architectures lies the neuron, an inherently oscillatory element that communicates through spiking signals. Self-sustained electronic oscillators emulate this behavior by autonomously generating periodic signals from constant energy input, regulating their internal dynamics through nonlinear feedback. Unlike passive LC resonators, which require external excitation, these active systems maintain their rhythm through NDR instabilities or feedback-amplifier loops, enabling spontaneous oscillation and adaptive timing. Such principles are not merely analogies to biology; they underpin emerging computing paradigms where synchronization, phase dynamics, and self-organization become the basis of computation.

This review has unified concepts from nonlinear dynamics, electrochemistry, electronic engineering, and computational neuroscience into a common theoretical framework. By describing oscillators through differential equations and bifurcation theory, particularly Hopf and related transitions, it becomes possible to connect physical device behavior with the mathematical structure of limit cycles. Compact oscillators based on functional materials such as VO_2_ and NbO_*x*_ memristors demonstrate the feasibility of achieving neuron-like spiking in scalable, low-power platforms. Meanwhile, feedback-based oscillators using transistor circuits express the same principles through explicit amplification, establishing a direct bridge between materials physics and circuit design.

Two primary architectures are thus distinguished and connected: oscillators based on NDR and those sustained by active-feedback gain. Hybrid models combine both mechanisms, enabling controllable frequency, tunability, and coupling. When assembled into ONNs, these devices can compute in the phase domain, offering analog, low-power alternatives to digital AI systems. Applications such as edge detection and associative learning illustrate how synchronization and phase locking can replace numerical operations with physical dynamics. This framework supports the emerging paradigm of OBC, which leverages self-organization and phase coherence to solve complex problems in pattern recognition, learning, and decision-making.

Beyond electronics, oscillatory behavior also governs chemical, optical, and biological systems, emphasizing the universality of nonlinear feedback and limit cycles. Understanding these mechanisms within a unified dynamical perspective offers chemists, physicists, and engineers a roadmap toward devices that think and adapt through oscillation. Table 2 provides a list of acronyms used in this work.

**Table 2:** List of Acronyms

AI	Artificial Intelligence
ANN	Artificial Neural Network
BZ	Belousov–Zhabotinsky reaction
CALM	Conductance-activated quasi-linear memristor model
CMOS	Complementary metal-oxide semiconductor technology
FHN	FitzHugh-Nagumo neuron model
HH	Hodgkin-Huxley model
LAO	Large amplitude oscillations
MIT	Metal insulator transition
MMO	Mixed-mode oscillations
NDR	Negative differential resistance
OBC	Oscillator based computation
OECT	Organic Electrochemical Transistors
ONN	Oscillatory Neural Networks
PRC	Phase response curve
RRAM	Resistive random-access memory
SAO	Small amplitude oscillations
SN	Saddle-node bifurcation
SNIC	Saddle-node on invariant circle bifurcation
SNN	Spiking Neural Networks
TS	Threshold switching
VCO	Voltage-controlled oscillator

## 9 Scope and Outlook

Future progress in oscillatory neuromorphic systems, OBC and other unconventional computational schemes will depend on integrating three complementary directions. (i) Materials development–advancing controllable active media such as correlated oxides, halide perovskites, and mixed ionic–electronic conductors to achieve reproducible, low-voltage oscillations. (ii) Circuit integration–embedding these oscillators into scalable arrays with programmable coupling and synchronization control for analog signal processing. (iii) Modeling frameworks–linking device-level nonlinear dynamics with system-level computation through multiscale models that unify chemical, electronic, and biological oscillators. Progress along these axes will transform oscillatory devices from physical curiosities into practical computing elements, bridging the conceptual gap between neurons, circuits, and materials.

## Figures and Tables

**Figure 34:**
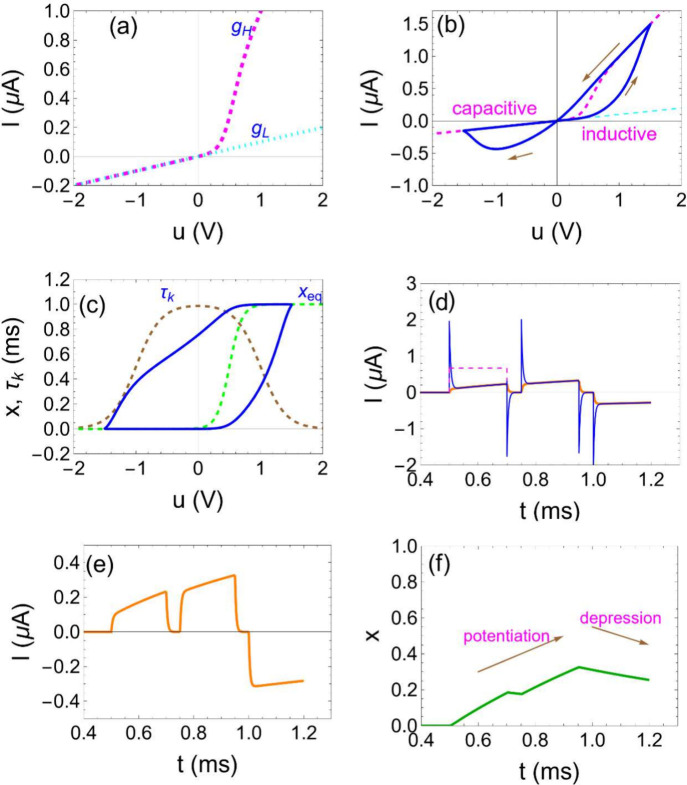
Hysteresis in the CALM model. (a) Current voltage curve (purple) indicating the low conductance branch (cyan). (b, c) Hysteresis in *IV* curves and memory variable *x* under sinusoidal stimulation *u* = *U*_0_ sin­(Ω_*st*_) at amplitude *U*_0_ = 1.5 *V* at frequency *f*_Ω_ = 0.2 Hz. (b) Current–voltage loops. The straight lines are the dc (purple) and low (cyan) conductance. (c) *x*-variable loops, indicating the *x*_*eq*_ (green) and relaxation time (brown). Initial condition *V*_0_ = 0, *x*_0_ = 0. (d-f) Current response to three voltage pulses applied to the CALM model: (Δ*V*, Δ*V*, −Δ*V*). Transient parameters *V*_0_ = 0 *V*, Δ*V* = 2 *V*, Δ*t*_*p*_ = 0.2 *ms*, Δ*t*_*r*_ = 0.05 *ms* initial condition *u*_0_ = 0, *x*_0_ = 0. (d) The blue line is the total current, and the dashed magenta line is the equilibrium current under the applied voltage. Orange line is the conduction current *I*_*cond*_ shown in (e). (f) Memory variable. Relaxation times: *τ*_*k*_ (*V*_0_) = 1 *ms*, *τ*_*k*_ (*V*_0_ + Δ*V*) = 0.016 *ms*. The dashed line is the final equilibrium value at *V*_0_ + Δ*V*. Model parameters *g*_*L*_ = 0.1 μS, *g*_*H*_ = 1 μS, V_m_ = 0.1 V, g_s_ = 5 μS, and C_m_ = 0.05 × 10^–9^ F, and for the relaxation time τ_0_ = 1 ms, τ_M_ = 1 ms, *V*_*Bx*_ = 0.5 V, *V*_*A*_ = 1 V, *n*_*A*_ = 2, V_Bτ_ = −1 *V*, and *n*_*D*_ = 2. Reproduced from ref 81. Available under a CC-BY 4.0 license. Copyright 2025 The Author(s). Published by Wiley-VCH (Wiley).^81^

**Figure 35:**
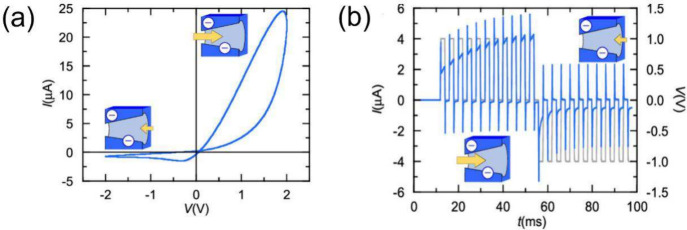
Ion conduction through multipore polyethylene terephthalate (PET) membranes. (a) *I–V* curve in 100 mM KCl solution at neutral pH. (b) Current response to a sequence of 2 ms positive voltage pulses) showing a gradual conductance potentiation followed by a sequence of negative voltage pulses, which reset the multipore membrane to the low conductance state for neutral pH. Reproduced with permission from Ramirez, P. et al., *J. Phys. Chem. Lett*. **2023**, 14, 10930–10934. Copyright 2023 American Chemical Society.^367^

**Figure 36:**
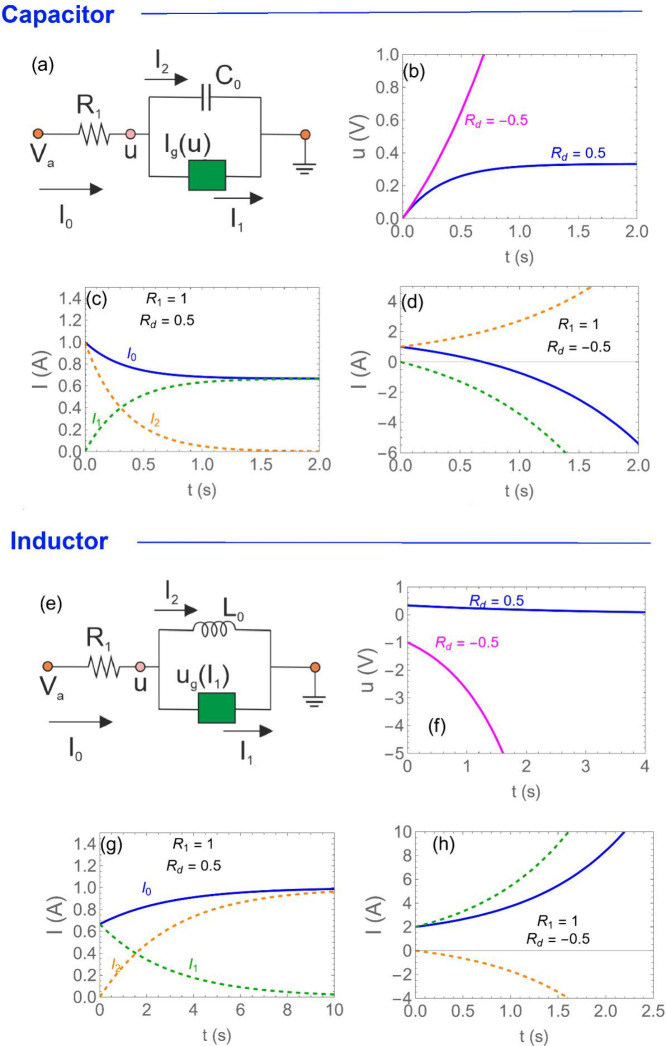
Transient behavior of positive and negative resistance under a voltage step Δ*V*. (a-d) Capacitive circuit. (e-h) Inductive circuit.

## Data Availability

The data presented here can be accessed at 10.5281/zenodo.17338256 (Zenodo) under the license CC BY 4.0 (Creative Commons Attribution 4.0 International).

## Appendix 1: Memristors for Synapse Applications

In artificial neuromorphic analog circuits, the communication between neuronic elements typically occurs through electrical signals in the form of voltages or currents. These signals can take on complex shapes, such as spike trains–repetitive pulses with constant amplitude–that emulate the action potentials observed in biological neurons. In such systems, synapses play a critical role by modulating the strength of the connection between neurons. The modulation is achieved through a memory state variable, *x*, which controls the synaptic conductance, *g*, effectively encoding the computational weight of the neural link, as shown in Figures 1 and 2. Memristor based synapses have been reviewed recently,^81,347^ and a brief summary is provided here for the sake of completeness, as many (though not all) dynamical features of oscillator neurons already exist in such systems.

To replicate the learning behavior observed in the brain–particularly the Hebbian learning^348^–artificial synapses must be capable of adapting their weights in response to incoming signals.^7,349^ This functionality is commonly implemented using two-terminal memristor devices,^2,176^ whose conductivity can be tuned by external stimuli to store and update synaptic weights. A wide variety of memristors with the resistive switching property have been studied for analog memory applications in neuromorphic systems.^71,350–363^

To summarize synaptic and memristor properties,^176,207,209^ we show a CALM model^81,364^

A1.1
$$I_{tot}\left(u\right)=\left[g_{L}+\left(g_{H}-g_{L}\right)x\right]u+C_{0}\frac{du}{dt}$$

The current *I*_*tot*_ in Eq [Disp-formula eqA1.1] depends on the voltage *u* and a state variable *x*. It contains two contributions, a capacitive current, and the conduction current, that switches between low (*g*_*L*_) and high (*g*_*H*_) conductance values when the activation function passes from *x* = 0 to *x* = 1. The adaptation equation has the form

A1.2
$$\tau_{k}\left(u\right)\frac{dx}{dt}=x_{eq}\left(u\right)-x$$

so that in equilibrium *x* → *x*_*eq*_, where the equilibrium function *x*_*ea*_ has typically a sigmoidal form such as

A1.3
$$x_{eq}=\frac{1}{1+e^{-\left(u-V_{B}\right)/V_{m}}}$$

*V*_*B*_ is a threshold potential for the activation of *x*, and the voltage *V*_*m*_ describes the steepness of the transition. This form is normal in many solid-state and biological systems.^365^ In Eq [Disp-formula eqA1.2] the *τ*_*k*_ is a relaxation time that is a unique function of the voltage *u*.^366^ As mentioned before, Eqs [Disp-formula eqA1.1] and [Disp-formula eqA1.2] are the essential form of a chemical inductor^177^ and also the typical constitutive equations of memristors. Figure 34 shows that the memory effect in the variable *x* produces the resistive switching and synaptic potentiation and depression.^81^ An experimental example for a fluidic nanochannel is shown in Figure 35.^367^

IF neurons are usually obtained combining a TS memristor with a nearly square cycling shape, with a capacitor.^67–70^

## Appendix 2: Dynamical Properties of the NDR

We analyze, first, an elementary dynamical model containing a positive or NDR in parallel with a capacitor as shown in Figure 36a. In this model the green element is nonlinear but it does not contain a dynamic delay (like the chemical inductor models). Therefore, the system can be described only by one differential equation.

Consider an N-type nonlinear element characterized by a conduction function *I*_*g*_ (*u*) with NDR, as shown in Figure 10c. We discuss the transient in a capacitive circuit with an auxiliary series resistance *R*_1_. The total current is given by

A2.1
$$I_{0}=I_{g}\left(u\right)+C_{0}\frac{du}{dt}$$

and the relationship of the voltages is

A2.2
$$V_{a}=R_{1}I_{0}+u$$

To obtain the dynamic behavior we imagine the system situated in a stationary point *u*_0_ as those shown in Figure 10c, and then we change the voltage by a small step value Δ*V*. The change of current is

A2.3
$$\Delta I_{0}=\frac{1}{R_{d}}\Delta u+C_{0}\frac{d\Delta u}{dt}$$

where the differential resistance is

A2.4
$$R_{d}={\left(\frac{dI_{g}}{du}\right)}^{-1}$$

Let us calculate the AC impedance. We transform the small signal Eq [Disp-formula eqA2.3] to the frequency domain by the Laplace transform, *d*/*dt* → *iω*

A2.5
$$\Delta I_{0}=\left(\frac{1}{R_{d}}+C_{0}i\omega \right)\Delta u$$

Combining with Eq [Disp-formula eqA2.2] we have the impedance

A2.6
$$Z\left(\omega \right)=\frac{\Delta V}{\Delta I_{0}}=R_{1}+\frac{R_{d}}{1+i\omega \tau_{C}}$$

where we have introduced the characteristic time

A2.7
$$\tau_{C}=R_{d}C_{0}$$

Note that the green element in Figure 36a becomes a resistance, *R*_*d*_, which can be positive or negative. The sign of the NDR determines the sign of *τ*_*C*_, since *C*_0_ is positive.

We now analyze the transient to a step voltage, to obtain the signature of stability by local analysis, following the criteria introduced by Lyapunov. We write Eq [Disp-formula eqA2.3] as

A2.8
$$C_{0}\frac{d\Delta u}{dt}=\frac{\Delta V}{R_{1}}-\frac{1}{R_{p}}\Delta u$$

where the parallel resistance is

A2.9
$$R_{p}=\frac{R_{1}R_{d}}{R_{1}+R_{d}}$$

The solution for Δ*u* = 0 at *t* = 0 is

A2.10
$$\Delta u\left(t\right)=\frac{R_{d}}{R_{1}+R_{d}}\Delta V\left(1-e^{-t/\tau_{1}}\right)$$

A2.11
$$\Delta I_{1}\left(t\right)=\frac{\Delta u}{R_{d}}$$

A2.12
$$\Delta I_{2}\left(t\right)=\frac{\Delta V}{R_{1}}e^{-t/\tau_{1}}$$

where

A2.13
$$\tau_{1}=R_{p}C_{0}$$

The transient currents for a positive resistance *R*_*d*_ are shown in Figure 36c. The total current decreases from the initial value Δ*V*/*R*_1_ to the final value Δ*V*/(*R*_1_ + *R*_*d*_). At the initial instant the whole current is capacitive, since Δ*u* = 0, Figure 36b. When the capacitor charges Δ*u* increases and the capacitive current vanishes, while the current becomes a full conduction current through *I*_1_.

For the NDR the result is very different, Figure 36d. Starting again at the value Δ*V*/*R*_1_, Δ*u* increases to charge the capacitor, Figure 36b. But then it creates a negative *I*_1_, which makes the capacitive current increase to compensate this rise. The more the negative *I*_1_ increases, the more *I*_2_ is needed, in a positive feedback process.

We make the same analysis for an S-type element as shown in Figure 10d, with nonlinear conduction function *u*_*g*_ (*I*), and a parallel inductor, Figure 36e. In this case, the transient equation is

A2.14
$$L_{0}\frac{dI_{2}}{dt}=u_{g}\left(I_{1}\right)$$

The impedance gives

A2.15
$$Z\left(\omega \right)=R_{1}+\frac{R_{d}}{1+\frac{1}{i\omega \tau_{L}}}$$

Here, the differential resistance is

A2.16
$$R_{d}=\frac{du_{g}}{dI}$$

and the characteristic time is

A2.17
$$\tau_{L}=\frac{L_{0}}{R_{d}}$$

For a small voltage step, using the circuit equations and Eq [Disp-formula eqA2.14], we obtain the transient equation

A2.18
$$\tau_{2}\frac{d\Delta I_{2}}{dt}=\frac{\Delta V}{R_{1}}-\Delta I_{2}$$

where

A2.19
$$\tau_{2}=\frac{L_{0}}{R_{p}}$$

and the transient is represented by the equations

A2.20
$$I_{2}\left(t\right)=\frac{\Delta V}{R_{1}}\left(1-e^{-t/\tau_{2}}\right)$$

A2.21
$$\Delta u\left(t\right)=R_{p}\frac{\Delta V}{R_{1}}e^{-t/\tau_{2}}$$

The ordinary transient current of an inductor is an increase at long time shown in Figure 36g. The impedance of the inductor decreases at low frequency and finally vanishes, so that Δ*u*(0) = Δ*V R*_p_/*R*_1_ while Δ*u* = 0 when *t* ≫ τ_2_, and the total resistance decreases, Figure 36f. For NDR the initial Δ*u* is negative. For a larger external current, Δ*u* becomes more negative, causing positive feedback, but with the total current increasing, Figure 36h, opposite to the situation of the capacitor.

The analysis of linearized systems that has been explained is a main tool of the methods of study of larger dynamical systems described in Secs. [Sec sec4]–[Sec sec7]. The basic capacitor and inductor, when combined with a NDR element, produce exponential responses with time, and the value of the characteristic time, τ, positive or negative, implies decay to equilibrium or unbound increase. However, the previous analysis refers only to the initial part of the trajectory. We have not solved a full nonlinear model, indicated in Eqs ([Disp-formula eqA2.1]) and ([Disp-formula eqA2.14]). In fact it is possible to obtain one-dimensional systems that show oscillations, although the resistance function must include some switching effect, see the chapter 4 of the book by Andronow and Chaikin.^17^ This is the case of relaxation oscillations that we have discussed throughout the main text.

Finally, we comment briefly on Liapunov stability theory, which is a powerful method for analyzing the behavior of dynamical systems near an equilibrium point without having to solve the system explicitly. Consider a system described by *ẋ* = *f* (*x*) where *x* = 0 is an equilibrium point (i.e., *f*(0) = 0). To study the stability of this point, we introduce a scalar function *U*(*x*) called a Liapunov function, which plays a role similar to energy. If *U*(*x*) is positive definite for all *x* ≠ 0, and its time derivative along system trajectories, is negative semidefinite (i.e., *U̇*(*x*) ≤ 0), the equilibrium is stable in the sense of Liapunov: small perturbations stay small. If *U̇*(*x*) < 0 (negative definite), the equilibrium is asymptotically stable, meaning the system returns to equilibrium over time. However, if U̇(x) > 0 in some region, the equilibrium is unstable. An important note is that the Liapunov method is sufficient but not necessary: finding such a function guarantees stability, but not finding one does not mean the system is unstable–it may simply require a different choice of function or approach. This makes Liapunov’s method especially useful for nonlinear systems where linear approximations may fail, such as in systems with zero or purely imaginary eigenvalues where classical linear stability analysis is inconclusive.

## Appendix 3: Characteristic of the Hopf Bifurcation

This Appendix is a very brief survey of a complex subject. For a detailed analysis of the properties of the limit cycles we refer the reader to the standard books.^22–25^

The eigenvalues λ of the matrix *J*_*X*_ satisfy the equation

A3.1
$$\det\left\{J_{X}-\lambda I\right\}=0$$

where **I** is the identity matrix. We arrive at the quadratic equation

A3.2
$$\lambda^{2}-T_{\lambda }\left(Y_{0}\right)\lambda +\Delta_{\lambda }\left(Y_{0}\right)=0$$

The solution of Eq. [Disp-formula eqA3.2] is

A3.3
$$\lambda_{\pm }=\frac{1}{2}\left[T_{\lambda }\left(Y_{0}\right)\pm {\left({T_{\lambda }}^{2}\left(Y_{0}\right)-4\Delta_{\lambda }\left(Y_{0}\right)\right)}^{1/2}\right]$$

A stationary point ***Y***_0_ for which **Δ**_λ_ (***Y***_0_) > 0 is said to be (asymptotically) stable if ***T***_λ_ (***Y***_0_) < 0; in this case, nearby solutions are attracted to this point. Conversely, if T_λ_ (Y_0_) > 0 the point is unstable, and nearby solutions are repelled from it. In both scenarios, the stationary point is referred to as hyperbolic.

To clarify the interpretation, let us define a characteristic time constant

A3.4
$$\tau_{\lambda }\left(y_{0}\right)=-\frac{1}{\alpha }=-\frac{2}{T_{\lambda }\left(Y_{0}\right)}$$

and a characteristic frequency

A3.5
$$\omega_{\lambda }\left(Y_{0}\right)={\left[\Delta_{\lambda }\left(Y_{0}\right)-\frac{1}{{\tau_{\lambda }}^{2}\left(Y_{0}\right)}\right]}^{1/2}$$

We can write the eigenvalues (Eq. [Disp-formula eqA3.3]) as

A3.6
$$\lambda_{\pm }=-\frac{1}{\tau_{\lambda }\left(Y_{0}\right)}\pm i\omega_{\lambda }\left(Y_{0}\right)$$

A new set of coordinates ***V*** = *P* ^–1^ ***Y*** can be found by a matrix *P* such that the system

A3.7
$$\frac{d}{dt}\left[\begin{array}{l} \Delta v_{1}\\ \Delta v_{2} \end{array}\right]=J_{V}\left[\begin{array}{l} \Delta v_{1}\\ \Delta v_{2} \end{array}\right]$$

provides a diagonal form

A3.8
$$J_{V}=\left[\begin{array}{ll} -\frac{1}{\tau_{\lambda }\left(y_{0}\right)}+i\omega_{\lambda }\left(Y_{0}\right) & 0\\ 0 & -\frac{1}{\tau_{\lambda }\left(y_{0}\right)}-i\omega_{\lambda }\left(Y_{0}\right) \end{array}\right]$$

According to the analysis of Appendix 2, circuits respond with time according to the respective characteristic times, so that τ > 0 leads to a stable situation while τ < 0 produces an exponential explosion. As the integral of Eq. [Disp-formula eqA3.7] gives an exponential function with λ ± multiplied by time as exponents, we observe that the linear analysis of the two-variable system produces a generalization Eq [Disp-formula eqA3.5], in which T_λ_< 0 → *τ*_*λ*_> 0 is a stable situation and conversely Tλ > 0 → *τ*_*λ*_ < 0 is unstable. As we pointed out in Figure 10, when the circuit contains both *L* and *C* a back-and-forth process occurs in which the energy is exchanged between both elements. This is represented by the additional contribution with the imaginary component­(*i ω*_*λ*_) in Eq [Disp-formula eqA3.6], that produces oscillatory behavior. For *τ*_*λ*_ > 0 the oscillations are damped, and for *τ*_*λ*_ < 0 a limit cycle oscillation appears.

Right at the Hopf bifurcation, when ***Y***_0_ = ***Y***_*B*_, and *T*_*λ*_(***Y***_*B*_) = 0, we have

A3.9
$$J_{V}=\left[\begin{array}{ll} +i\omega_{0B}\left(Y_{B}\right) & 0\\ 0 & -i\omega_{0B}\left(Y_{B}\right) \end{array}\right]$$

where

A3.10
$$\omega_{0B}\left(Y_{B}\right)={\left[\Delta_{\lambda }\left(Y_{B}\right)\right]}^{1/2}$$

In combination with Eq [Disp-formula eqA3.4] this is a linear system that produces sinusoidal oscillations of frequency ω_0*B*_. However, the form of the limit cycle is determined by nonlinear terms of the expansion of Eqs ([Disp-formula eq6], 7).
